# Giant tunable barocaloric effects using guest-molecule adsorption in a porous metal-organic framework

**DOI:** 10.1038/s41467-026-74616-6

**Published:** 2026-06-26

**Authors:** Melony Dilshad, Giulio I. Lampronti, Thomas D. Bennett, Xavier Moya

**Affiliations:** 1 Department of Materials Science & Metallurgy, University of Cambridge, Cambridge, UK; 2 MacDiarmid Institute for Advanced Materials and Nanotechnology, School of Physical and Chemical Sciences, University of Canterbury, Christchurch, New Zealand

**Keywords:** Metal-organic frameworks, Materials for devices

## Abstract

Metal-organic frameworks (MOFs) that exhibit first-order structural phase transitions with significant volume changes are promising candidates for barocaloric cooling and heating applications. Here we report giant barocaloric effects in a zeolitic-imidazolate framework [ZIF-4(Zn)] that can be reversibly driven over a wide range of temperatures with pressure, near a starting temperature that is highly tunable using gas adsorption. For nitrogen-loaded frameworks of ZIF-4(Zn), we report isothermal entropy changes |∆*S*_it_| > 38 J K^-1^ kg^-1^ and adiabatic temperature changes |∆*T*_ad_| > 10 K driven reversibly by |∆*p*| = |*p* - *p*_atm_| ~ |*p*| ~ 1 kbar (*p*_atm _~ 1 bar) near *T*_0_ ~ 190 K. For desolvated frameworks of ZIF-4(Zn), we estimate larger barocaloric effects up to |∆*S*_it_| ~ 210 J K^-1^ kg^-1^ and |∆*T*_ad_| ~ 60 K near *T*_0_ ~ 137 K driven using the same pressure change. Due to the dynamic behaviour of this porous MOF, a combination of quasi-direct, indirect and direct methods was used to determine the caloric performance. This combination of methods underlines the cooling and heating potential of porous MOFs, while highlighting the necessity of quantifying the presence of any inactive mass. Our findings should establish a benchmark for MOFs as environmentally benign solid-state refrigerants that can be tuned using guest molecules as a caloric vector.

## Abstract (web summary)

Giant barocaloric effects are demonstrated in a porous metal-organic framework, the operating temperature of which can be tuned via nitrogen adsorption as a caloric vector, thus demonstrating good potential for environmentally benign solid-state cooling and heating applications.

## Introduction

Barocaloric materials show nominally reversible thermal changes when driven by changes in applied hydrostatic pressure^1,2^. They are currently considered potential solid-state alternatives to conventional gas refrigerants used in vapour-compression cooling and heating systems, thus offering environmentally friendly solutions for thermal management^3^. Over the past decade, barocaloric effects in different classes of materials have been reported in a growing number of publications. These include spin-crossover compounds^4–9^, hybrid perovskites^10–14^, superionic conductors^15–18^, and plastic crystals^19–24^.

Metal-organic frameworks (MOFs) are a class of organic-inorganic hybrid materials composed of metal ions or clusters interconnected by organic ligands, with most structures reported to be porous crystalline three-dimensional networks^25^. MOFs that undergo reversible crystallographic phase transitions involving large changes in unit cell volume are potential barocaloric candidates. Here we report on the barocaloric effect in a porous MOF from the zeolitic imidazolate framework (ZIF) family, namely ZIF-4(Zn) [Zn(im)_2_; im = imidazolate], that has been predicted to display large barocaloric effects^26^. However, unlike traditional barocaloric materials, ZIF-4(Zn) displays certain unexpected dynamic barocaloric characteristics based on its ability to adsorb guest molecules due to its porosity. While the effect of adsorbed guest molecules into pores has been widely studied in this material and in other similar MOFs^27–33^, here we show that adsorption of nitrogen into ZIF-4(Zn) modifies its giant barocaloric properties. We experimentally demonstrate the impact of nitrogen adsorption on the transition temperature of its first-order crystallographic phase transition, and the reversibility of the barocaloric effect over a wide range of temperatures at relatively low applied pressures below 1 kbar, which are readily available using standard hydraulic components for applications. We use a combination of methods to probe the dynamic aspects of the caloric effect in ZIF-4(Zn) that are not typically observed in traditional caloric materials.

## Results

### First-order phase transition in desolvated ZIF-4(Zn) at ambient pressure

**Fig. 1:**
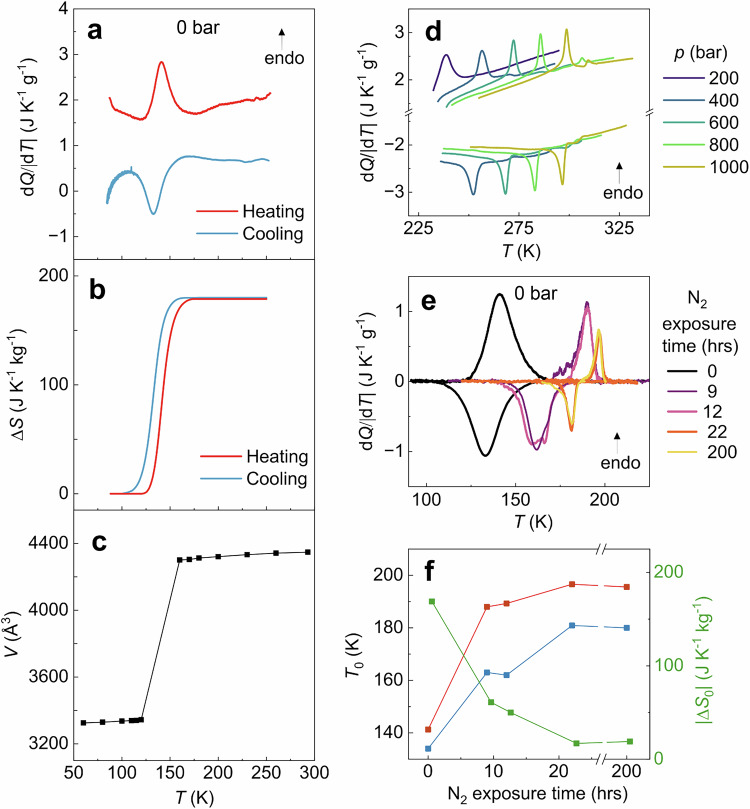
Thermodynamic analysis of the first-order crystallographic phase transition in ZIF-4(Zn). **a** Calorimetric peaks on heating and cooling desolvated ZIF-4(Zn) near *T*_0_ ~ 137 K at ambient pressure (*p* = 0 bar). **b** Transition-related entropy change Δ*S* as a function of temperature *T* with respect to the LT phase of desolvated ZIF-4(Zn). **c** Evolution of unit cell volume *V* of desolvated ZIF-4(Zn) with temperature *T* showing volume change associated with the phase transition. Data points were obtained from LT-XRD patterns (Supplementary Fig. 3). **d** Isobaric calorimetric data for ZIF-4(Zn) on heating and cooling under applied pressure *p* using nitrogen gas. **e**, **f** Qualitative assessment of high-pressure nitrogen exposure time on the transition in ZIF-4(Zn) at ambient pressure. Background-subtracted calorimetric peaks at ambient pressure on heating and cooling in (**e**) show comparison between desolvated ZIF-4(Zn) (0 h exposure) and ZIF-4(Zn) exposed for 9, 12, 22 and 200 h to nitrogen under applied *p* up to 1000 bar, and **f** shows *T*_0_ and |∆*S*_0_| associated with the first-order transition as a function of nitrogen exposure time derived from calorimetric data in (**e**). Red and blue squares represent the *T*_0_ values on heating and cooling, respectively. The |∆*S*_0_| values (green squares) have been normalised by total sample mass. Solid lines represent linear interpolation between points as guide to the eye. Endothermic (endo) peaks are defined with upward arrows for the calorimetry data in (**a**, **d**, **e**).

ZIF-4(Zn) was synthesised and activated (solvent evacuated from pores) according to a reported^34^ solvothermal synthesis procedure (see “Methods” for details). Following activation, the crystallographic structure of the desolvated ZIF-4(Zn) powder obtained was confirmed using X-ray diffraction (XRD) (Supplementary Fig. 1). At ambient temperature and pressure, the framework adopts an orthorhombic structure (*Pbca*) with a unit cell volume *V* ~ 4348 Å^3^. Ambient-pressure calorimetry indicates a first-order phase transition on cooling near *T*_0_ ~ 137 K, with a latent heat |*Q*_0_| ~ 23 kJ kg^−1^ and corresponding entropy change |∆*S*_0_| ~ 180 J K^−1^ kg^−1^ (Fig. 1a, b). The low-temperature (LT) phase was confirmed using XRD to be a denser orthorhombic structure, in which the *Pbca* space group is retained, but with a contracted unit cell volume of *V* ~ 3325 Å^3^ at *T* = 60 K (Supplementary Figs. 1 and 2). Both LT phase and ambient temperature phase (which we will denote as HT phase for high-temperature), and the thermodynamic parameters of the phase transition evaluated here are in agreement with previously reported values^34^. From a series of LT-XRD patterns (Supplementary Fig. 3), a volume change |∆*V*_0_|/*V* ~ 22% associated with the first-order phase transition in our desolvated ZIF-4(Zn) sample was evaluated (Fig. 1c), which corresponds to a specific volume change |∆*v*_0_| ~ 1.83 × 10^−4^ m^3^ kg^−1^ across the transition, using density *ρ* = 1.22 g cm^−3^ for the HT phase^34^. However, quantitative phase analysis using the LT-XRD patterns (Supplementary Fig. 4) suggests that ~15% of the sample remains in the HT phase below *T*_0_, which is likely due to the presence of some large crystals that do not undergo the transition to the LT phase^34^. In order to estimate the pressure-induced shift in transition temperature, d*T*_0_/d*p*, of this volume transition using the temperature-pressure Clausius-Clapeyron relation, we normalised |∆*S*_0_| by active mass, which implies |∆*S*_0_| ~ 212 J K^−1^ kg^−1^ associated with the phase transition. This suggests d*T*_0_/d*p* ~ 86 K kbar^−1^ in desolvated ZIF-4(Zn).

### First-order phase transition in ZIF-4(Zn) under applied pressure

Calorimetry under applied hydrostatic pressure was subsequently performed on ZIF-4(Zn) using nitrogen gas as the pressure-transmitting fluid (PTF) (Fig. 1d). These results indicate an experimental d*T*_0_/d*p* ~ 76 K kbar^−1^. The instrument specifications of our high-pressure (HP) differential scanning calorimeter (DSC) do not permit measurements below 228 K, and therefore the calorimetric peaks on both heating and cooling at ambient pressure could not be measured using this instrument (similarly on cooling at *p* = 200 bar, as shown in Fig. 1d). The powder specimen was thereby retrieved from the HP-DSC to re-evaluate the ambient-pressure calorimetric response after high-pressure measurements using a TA Q2000 DSC and a bespoke DSC instrument, which permits accessing lower temperatures.

### First-order phase transition in ZIF-4(Zn) at ambient pressure after high-pressure measurements

Following high-pressure calorimetry, ambient-pressure calorimetric measurements of the ZIF-4(Zn) sample showed two distinctive features:

1. The transition temperature of the first-order phase transition in the sample was much higher after high-pressure calorimetric measurements (*T*_0_ ~ 190 K) than before (*T*_0_ ~ 137 K).
2. The entropy change of the first-order phase transition, |∆*S*_0_|, normalised by total (active plus inactive) sample mass, was much smaller after high-pressure calorimetric measurements (|∆*S*_0_| ~ 16 J K^−1^ kg^−1^) than before (|∆*S*_0_| ~ 180 J K^−1^ kg^−1^).

To test whether exposure to nitrogen gas in the HP-DSC had an effect on the first-order phase transition in ZIF-4(Zn), a qualitative study was performed by loading pristine desolvated powder specimens in the HP-DSC and exposing the samples to nitrogen gas for 9 and 12 h at set pressure *p* ~ 1000 bar and set temperature *T* ~ 233 K. The samples were then retrieved from the HP-DSC and run in the bespoke calorimeter for comparison of ambient-pressure calorimetric peaks with those obtained after longer nitrogen exposure following HP-DSC measurements (for 22 and 200 h), and the pristine desolvated sample (Fig. 1e, f). The results indicate that *T*_0_ and |∆*S*_0_| values of the sample exposed for 9 and 12 h lie in between those of the pristine sample and the samples exposed for 22 and 200 h, which suggest a correlation between high-pressure exposure to nitrogen and the thermodynamic properties of the first-order phase transition in ZIF-4(Zn).

The increase in *T*_0_ after nitrogen exposure suggests that the LT phase of ZIF-4(Zn) with adsorbed nitrogen is more stable than that of the desolvated ZIF-4(Zn) sample. This is likely due to the enthalpic stabilisation of the nitrogen-loaded framework at low temperature resulting from a lower framework enthalpy compared with the LT phase of the desolvated framework—a similar reduction in enthalpy has been reported from the adsorption of N,N-Dimethylformamide (DMF) into the ZIF-4(Zn) framework^35^, and different guest molecules into other porous MOFs^36,37^. The similarity of *T*_0_ for the 22 and 200 h exposure samples suggest a potential saturation of nitrogen adsorbed within the pores, and we will refer to this structure as nitrogen-loaded ZIF-4(Zn). In a similar context, the control of the operational temperature range in magnetocaloric materials by absorption of gas was proposed two decades ago^38^, when it was reported that absorption of hydrogen into the prototypical magnetocaloric material La(Fe_0.88_Si_0.12_)_13_ was able to increase its Curie temperature by up to ~130 K. However, it was later shown that these changes were unstable^39^.

To understand the reduction in |∆*S*_0_| in Fig. 1f, another series of LT-XRD patterns was obtained for the nitrogen-loaded ZIF-4(Zn) sample after HP-DSC measurements (Supplementary Fig. 8). This new series of XRD patterns indicated a large phase fraction (~43%) at 293 K of a different phase, ZIF-*zni*, which is reported to be the most thermodynamically stable ZIF structure^35^ for this composition (Supplementary Fig. 11). This suggests that after prolonged high-pressure exposure to nitrogen, a large fraction of crystals have transformed to ZIF-*zni*. The formation of ZIF-*zni* from ZIF-4 within this temperature and pressure regime (228 K ≤ *T* ≤ 343 K, 0 bar ≤ *p* ≤ 1200 bar) has not previously been reported—most reports^40,41^ show the irreversible transition of ZIF-4 to an amorphous form of ZIF (*a*-ZIF) on heating above 523 K, which then recrystallises on further heating above ~623 K to ZIF-*zni*. (It is worth highlighting that these studies were performed with different PTFs.) Once formed, this ZIF-*zni* is reportedly^40,42^ stable down to ambient conditions and is not reported to show any transitions below 343 K. Our LT-XRD patterns also show that the ZIF-*zni* phase fraction in our sample remains mostly constant throughout the temperature range the sample is subjected to (Supplementary Figs. 8 and 9). Furthermore, our LT-XRD patterns indicate that a phase transition occurs reversibly at *T*_0_ ~ 183 K on cooling and *T*_0_ ~ 193 K on heating, which corroborate well the experimental *T*_0_ values of our nitrogen-loaded ZIF-4(Zn) sample evaluated using ambient-pressure DSC after high-pressure measurements (Fig. 1f).

Below *T*_0_, it is clear that there are at least three phases present—ZIF-*zni*, ZIF-4(Zn) HT phase, and ZIF-4(Zn) LT phase (Supplementary Fig. 12). However, refining the XRD patterns below 183 K proved difficult: the LT structure of the ZIF-4(Zn) phase could not be confidently described because the signal was largely covered by peaks from ZIF-*zni* and the ZIF-4(Zn) HT phase. It is possible that the LT phase of our nitrogen-loaded ZIF-4(Zn) sample is a structure that has not been previously reported. Qualitatively, it can be deduced that this structure is a slightly distorted version of the LT structure of our desolvated sample, and is likely to be similar to the monoclinic structure previously reported^43,44^ as ZIF-4(Zn)-cp, which is the structure of desolvated ZIF-4(Zn) subjected to applied mechanical pressure, *p* ~ 750 bar. This structure is described as being similar to the LT structure of desolvated ZIF-4(Zn), with a unit cell volume that is ~20% lower than the orthorhombic HT phase, but with a slight shear element increasing the *β* angle. However, due to the necessary structural constraints used for the refinement of our LT structure, there cannot be full confidence in the values extracted for the unit cell volumes and for the quantification of the three phases in the XRD patterns below 183 K. In contrast, the patterns above 183 K were accurately refined, and data extracted from them can be confidently accepted, including the volumes and phase fractions of the HT phase of nitrogen-loaded ZIF-4(Zn) and ZIF-*zni*.

It is not uncommon for various structures of ZIFs (and MOFs in general) to exist or compete at a given value of temperature and pressure. Many porous framework structures of a given topology have very similar energy landscapes, and the predominant phase highly depends on path-specific details such as kinetics, occupancy of pores, type of PTF used and crystal size^41^. When using non-penetrating PTFs such as mercury^43^, Fluorinert FC-70^43^ or silicone oil^44^, no transition to ZIF-*zni* has been reported under the temperature and pressure conditions used in our work. Moreover, this structural phase transition in ZIF-4(Zn) is fully reversible when driven using silicone oil (AP 100), with its large transition |∆*V*_0_|/*V* ~ 20% and original ZIF-4(Zn) HT orthorhombic structure retained^44^ after having been exposed to much higher pressures of *p* = 4 kbar.

The presence of the HT phase of nitrogen-loaded ZIF-4(Zn) below *T*_0_ indicates that not all of our ZIF-4 specimen transforms to the LT phase, as observed in the sample before pressurisation under nitrogen. However, in addition to the large crystals which do not undergo the transition to the LT phase, there is likely to be further deactivation of ZIF-4(Zn) due to excessive nitrogen adsorption into the pores. The potential for adsorbed matter to prevent the transition of the porous structure of ZIF-4(Zn) to the contracted structure at low temperature has been previously reported^34^. Given that the nitrogen-loaded ZIF-4(Zn) sample showed only one calorimetric peak near *T*_0_ ~ 190 K that was not broad, and no discernible additional peaks were observed down to the original *T*_0_ ~ 140 K of the desolvated structure, it can be concluded that all the crystals in the sample specimen have adsorbed some nitrogen within their pores, and that there could be a threshold amount of adsorbed nitrogen above which the crystals would be ‘deactivated’, preventing the transition to the contracted phase. Nevertheless, the presence of ~43% ZIF-*zni* phase and some non-transforming ZIF-4 phase in the measured sample explains the reduced values of |∆*S*_0_| obtained from integral areas of calorimetric peaks after high-pressure calorimetry when normalised by total sample mass.

### Quasi-direct evaluation of the barocaloric performance of ZIF-4(Zn)

**Fig. 2:**
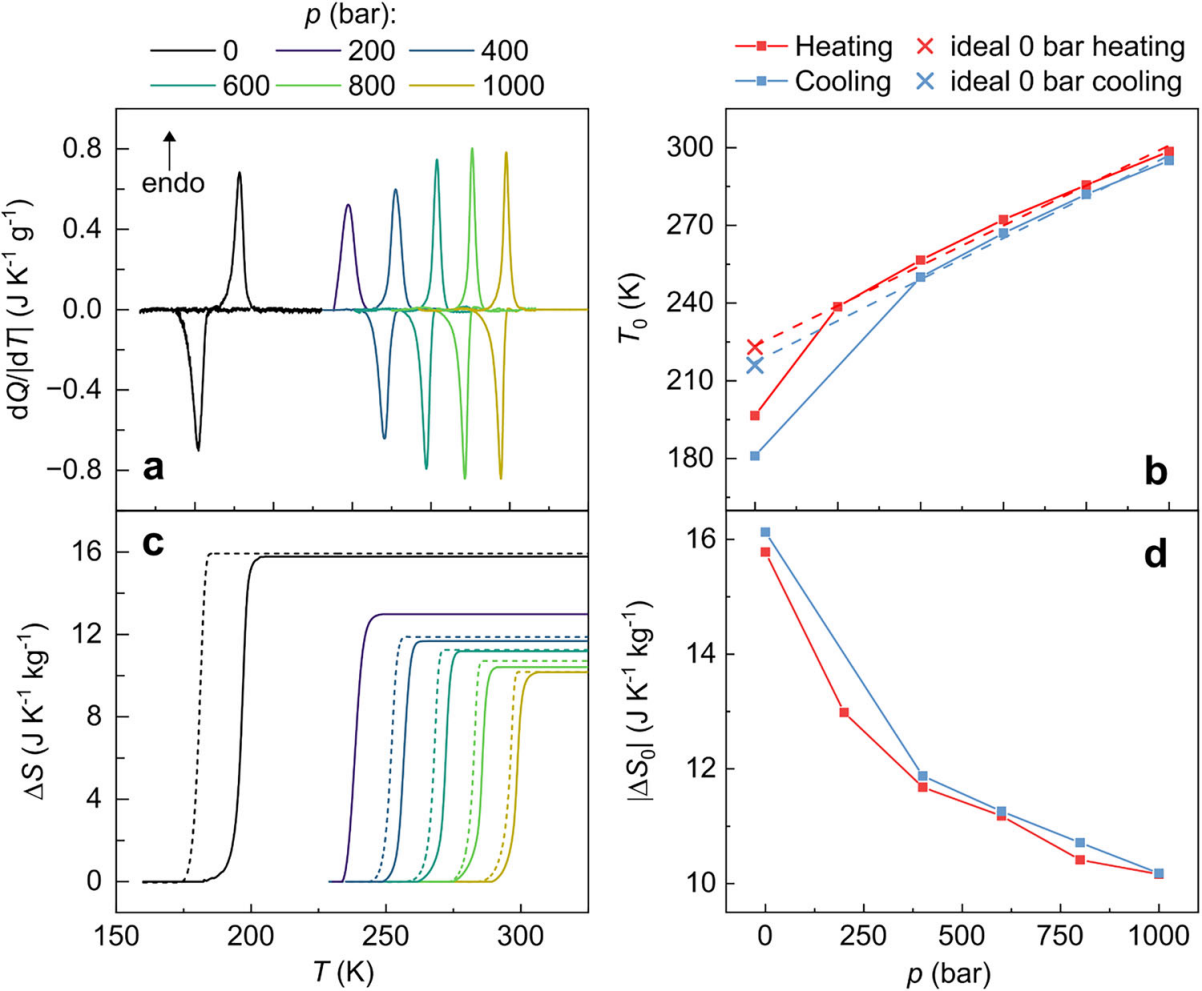
Analysis of the first-order crystallographic phase transition in nitrogen-loaded ZIF-4(Zn) from isobaric calorimetry after high-pressure exposure to nitrogen. **a** Background-subtracted isobaric d*Q*/|d*T*| as a function of *T* under different values of applied *p*. The upward arrow defines endothermic (endo) peaks. **b** Corresponding *T*_0_ values on heating and cooling as a function of applied *p*. Solid lines represent linear interpolation between all experimentally obtained data points. Dotted lines represent linear extrapolation of data points between 200 bar and 1000 bar to evaluate the ideal *T*_0_ values on heating (red cross) and cooling (blue cross) at ambient pressure (*p* = 0 bar) after high pressure measurements. **c** Isobaric functions of transition-related entropy change ∆*S*(*T*) at different applied *p* on heating (solid lines) and cooling (dotted lines), with respect to the LT phase, derived from calorimetric data in (**a**). **d** Transition |∆*S*_0_| as a function of applied *p* derived from (**c**). Values in **a**, **c**, **d** have been normalised by total sample mass.

The complete set of background-subtracted isobaric calorimetric peaks near the crystallographic phase transition in nitrogen-loaded ZIF-4(Zn) from *p* = 0 bar to 1000 bar is shown in Fig. 2, in which the ambient-pressure (*p* = 0 bar) measurement shown was obtained after high-pressure measurements (22 h sample in Fig. 1f). Figure 2b shows the dependence of transition temperature *T*_0_ on applied pressure *p*. The sample reproducibly showed an ambient-pressure *T*_0_ ~ 190 K when measured using both the TA Q2000 DSC and the bespoke DSC after removal from the HP-DSC. This experimentally measured ambient-pressure *T*_0_ value is lower than that suggested by the *T*_0_-*p* trend from the high-pressure calorimetric measurements. Figure 2b shows a projected linear extrapolation of *T*_0_ values from high-pressure calorimetric data which suggest an ideal ambient-pressure *T*_0_ ~ 218 K. While the derivative of the linear fit of the high-pressure data is d*T*_0_/d*p* ~ 76 K kbar^−1^, the derivative of the *T*_0_-*p* graph near *p* = 0 bar using our experimental *T*_0_ ~ 190 K suggests d*T*_0_/d*p* > 200 K kbar^−1^, which appears unrealistic. Given that all DSC instruments used in our experiments have been calibrated for accurate temperature measurements, this reduction in ambient-pressure *T*_0_ suggests that some adsorbed nitrogen within the ZIF-4(Zn) framework could be getting desorbed after depressurisation and subsequent sample transfer to the ambient-pressure DSC instruments. This is consistent with a number of reports of nitrogen adsorption in ZIF-4(Zn) in the literature^30,45–47^, as described below.

From previous experimental measurements, nitrogen gas adsorption–desorption isotherms of desolvated ZIF-4(Zn) have been reported^30,45,46^ starting from vacuum pressures (down to 10^−9 ^bar) up to ambient pressure at 77 K, 273 K and 293 K. On pressurizing to ~1 bar at 77 K, some studies^30,45^ report an adsorption volume of ~270 cm^3 ^g^−1^ under equilibrium conditions that took ~24 h for a full measurement^30^, whereas another study^46^ reports ~15 cm^3 ^g^−1^, which is much lower likely due to kinetics. All three studies show remnant adsorbed nitrogen in the pores after full depressurisation at 77 K. However, at 273 K and 293 K, the desorption isotherm from the study^46^ that reported lower adsorption volumes showed no remnant nitrogen after depressurisation. These reports indicate that prolonged exposure to nitrogen can result in some remnant adsorbed nitrogen volume, with a higher adsorption favourability at low temperature. A computational study^47^ using Monte Carlo simulations reported nitrogen adsorption isotherms at 298 K from vacuum pressures (0.01 bar) to exceeding ambient pressure up to 100 bar, showing a steady increase in volume of adsorbed nitrogen beyond ~1 bar up to ~ 50 bar, before dropping by ~25% on reaching 100 bar. Our sample was exposed to nitrogen in our HP-DSC between ambient pressure and ~1200 bar, over temperatures between 228 K and 343 K. Taking into account the general favourability for desorption at higher temperatures^46^, and data from simulations^47^ of nitrogen-loaded ZIF-4, some excess adsorbed nitrogen is likely desorbed at a certain pressure-threshold below *p* ~ 200 bar within this temperature range, and during sample transfer at ambient temperature and pressure. The evacuation of this excess adsorbed nitrogen below this threshold pressure could explain the lower experimental *T*_0_ value at ambient pressure than that obtained from extrapolating high-pressure data, thereby deviating from the *T*_0_*-p* trend. Therefore, the estimated ideal d*T*_0_/d*p* ~ 76 K kbar^−1^ in nitrogen-loaded ZIF-4(Zn) sets a minimum boundary for the transition d*T*_0_/d*p* shift achievable from this series of measurements for the crystallographic phase transition when driven by applied hydrostatic pressure using nitrogen gas.

Figure 2c shows the isobaric functions of ∆*S*(*T*) associated with the first-order transition under applied *p* on heating and cooling through the transition with respect to the LT phase. Figure 2d shows the dependence of the transition |∆*S*_0_| on *p*. The decrease in transition |∆*S*_0_| with increasing *p* can be attributed to the additional entropy changes associated with volumetric thermal expansion of the HT and LT phases above and below the phase transition^20^. However, it has also been established from LT-XRD patterns that these |∆*S*_0_| values evaluated from integral areas of the calorimetric peaks from (and after) high-pressure measurements (Fig. 2a) are highly reduced due to mass-normalisation that includes inactive sample mass from ZIF-*zni* and large ZIF-4 crystals. Therefore, evaluation of the barocaloric effect in nitrogen-loaded ZIF-4(Zn) alone, associated with the crystallographic phase transition, requires a correction for the active sample mass. We note that coexistence of active and inactive material (mass) is common in magnetocalorics^48^ and electrocalorics^49^, and correction for active mass is standard practice.

**Fig. 3:**
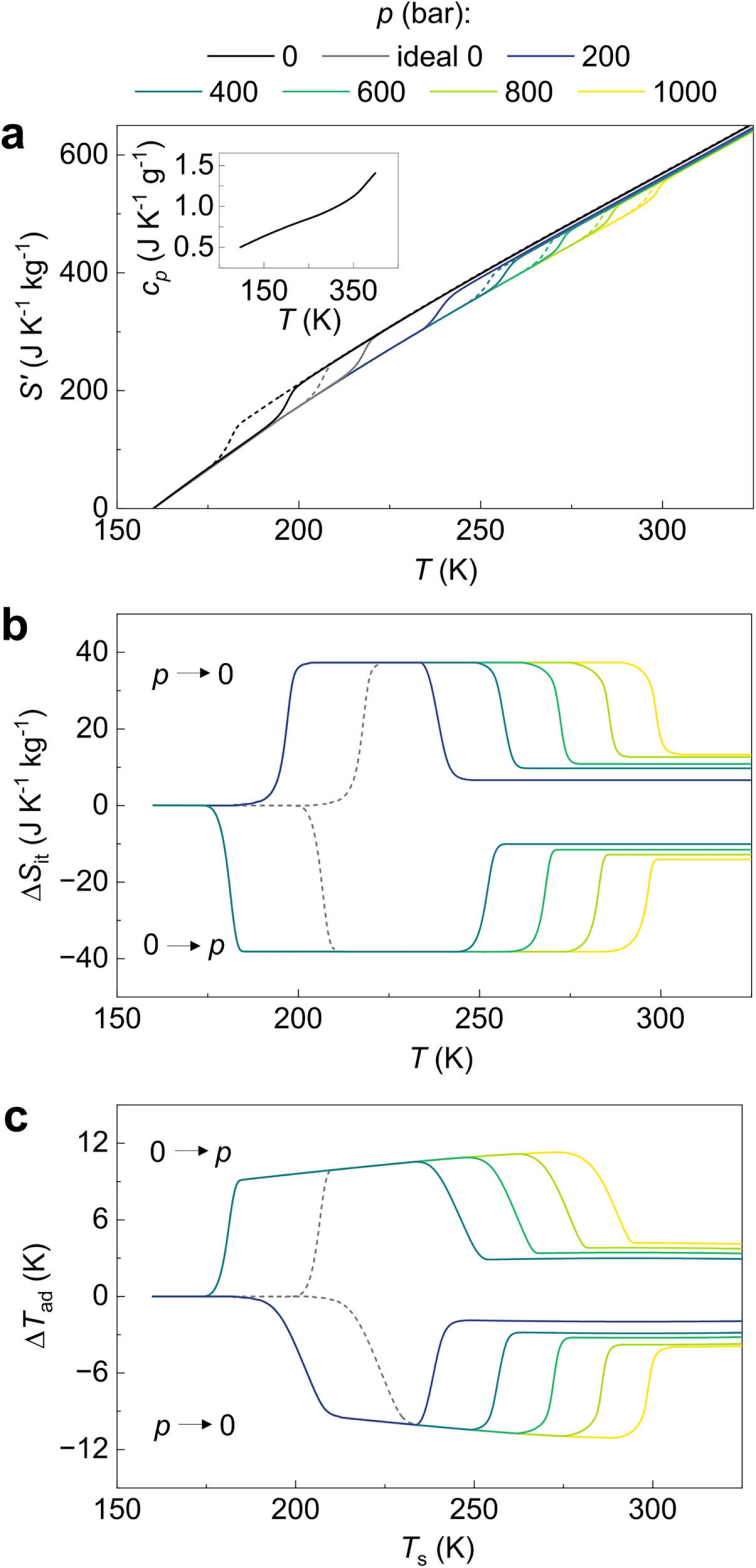
Quasi-direct evaluation of the minimum barocaloric effect in nitrogen-loaded ZIF-4(Zn) associated with the first-order phase transition. **a** Isobars in entropy-temperature space with respect to the entropy *S*(*T*) at *T* = 160 K on heating (solid lines) and cooling (dotted lines). The inset shows the baseline specific heat capacity *c*_*p*_ of ZIF-4(Zn). **b** Isothermal entropy change ∆*S*_it_ as a function of temperature *T* and **c** adiabatic temperature change ∆*T*_ad_ as a function of starting temperature *T*_s_ driven on applying (0 → *p*) and removing (*p* → 0) pressure near the phase transition. Additional entropy changes from volumetric thermal expansion of the LT and HT phases are not included. Grey dotted lines indicate the upper boundary of the *T*_0_ onset from projected ideal 0 bar conditions.

As no useful phase fraction or volume data could be extracted from our LT-XRD measurements for nitrogen-loaded ZIF-4 below *T*_0_, the active sample mass could not be accurately determined. However, using data that we can confidently extract from LT-XRD patterns of the sample before high-pressure measurements, and patterns above *T*_0_ of the sample after high-pressure measurements, we can conservatively evaluate the minimum barocaloric effect in nitrogen-loaded ZIF-4(Zn). The patterns before high-pressure measurements indicate that at least ~15% of the sample mass did not transform to the LT phase. Patterns after HP-DSC measurements indicate a phase fraction of ~43% of ZIF-*zni* in the evaluated sample. Therefore, it can be estimated that a minimum of ~58% of the nitrogen-loaded sample specimen is deactivated. Given that an ambient-pressure |Δ*S*_0_| ~ 16 J K^−1^ kg^−1^ was analysed when normalised by total mass following high-pressure calorimetry, we evaluate a minimum |Δ*S*_0_| ~ 38 J K^−1^ kg^−1^ when corrected for active mass. Thereby, the isobaric ∆*S*(*T*) functions (Fig. 2c) are renormalised by this active mass. The barocaloric effect in nitrogen-loaded ZIF-4(Zn) can then be evaluated quasi-directly by constructing isobars in entropy-temperature space using specific heat capacity measurements and the corrected isobaric ∆*S*(*T*) functions as shown in Fig. 3a. Figure 3b shows isothermal entropy changes (∆*S*_it_) as a function of temperature and Fig. 3c shows adiabatic temperature changes (∆*T*_ad_) as a function of starting temperature in nitrogen-loaded ZIF-4(Zn). This yields barocaloric parameters of |∆*S*_it_| ~ 38 J K^−1^ kg^−1^ and |∆*T*_ad_| ~ 10 K driven by |∆*p*| = 1 kbar near the crystallographic phase transition at *T*_0_ ~ 190 K. The release of excess nitrogen from ZIF-4 pores on depressurising down to *p* = 0 bar has positive implications on the temperature span for the reversible caloric effect: using the ideal *T*_0_ onset (shown in grey in Fig. 3), these barocaloric effects can be driven reversibly over a wide temperature span of ~70 K using |∆*p*| = 1 kbar, however this reversible temperature span could be increased up to ~90 K due to gas desorption (Fig. 3).

**Fig. 4:**
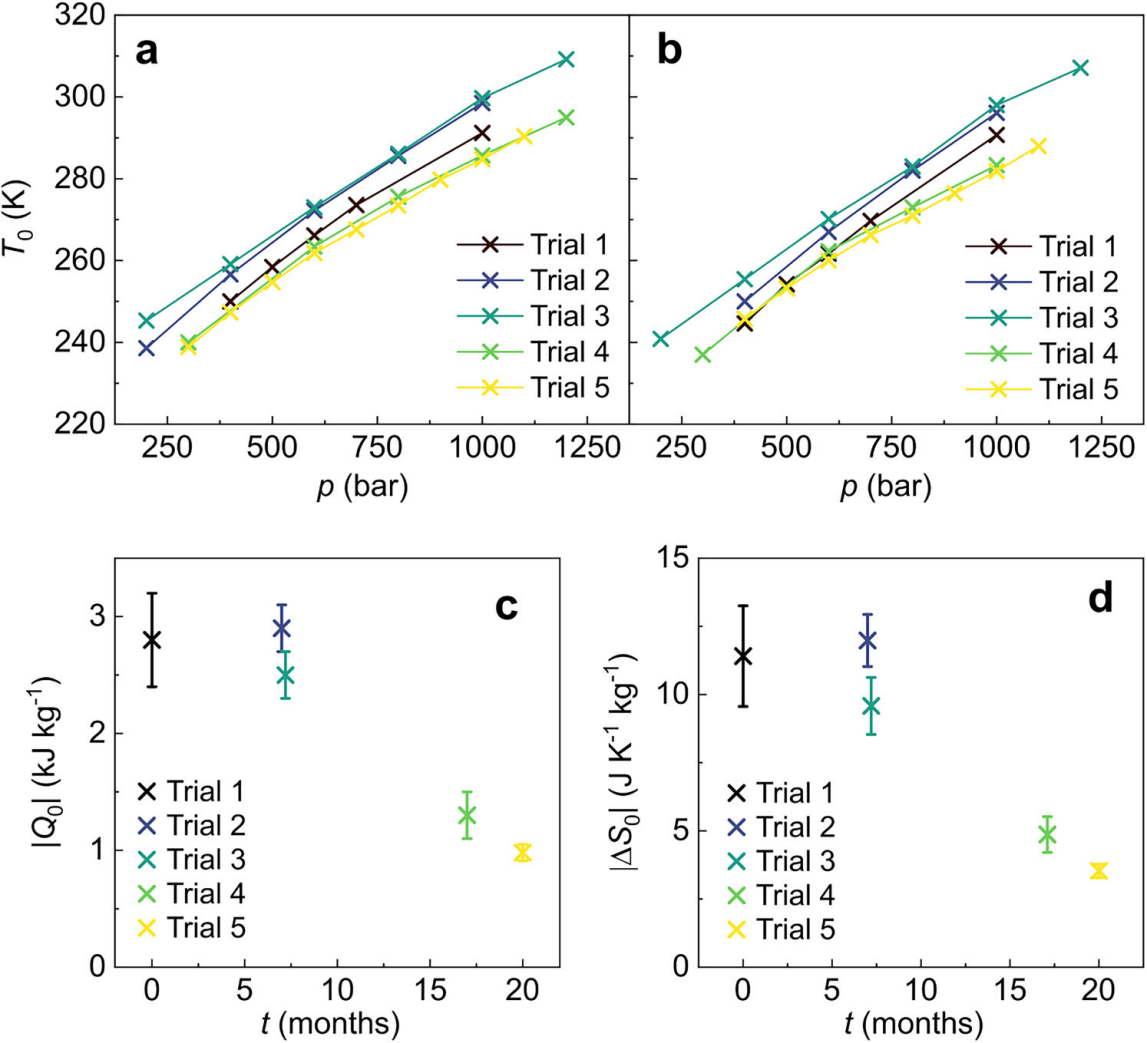
Thermodynamic parameters of the first-order phase transition in nitrogen-loaded ZIF-4(Zn) under applied *p* from five repeated trials (Trial 1–Trial 5) of isobaric calorimetry performed over time since the first isobaric measurement under high-pressure nitrogen (Trial 1). **a**, **b** Transition temperatures *T*_0_ of nitrogen-loaded ZIF-4(Zn) on (**a**) heating and (**b**) cooling under different values of applied *p* from repeated calorimetry trials. Solid lines represent linear interpolation between points as guide to the eye. Variation in **c** latent heat |*Q*_0_| and **d** transition entropy change |∆*S*_0_| of nitrogen-loaded ZIF-4(Zn) over the different trials. Error bars represent one standard deviation for values obtained isobarically at each applied *p* on heating and cooling for each trial, with a minimum sampling number *n* = 8.

To further probe this dynamic behaviour of ZIF-4(Zn), we performed repeated calorimetric trials under applied pressure on the same sample specimen after prolonged periods of time. Supplementary Table 1 indicates the time elapsed for each calorimetric trial since the first performed measurement, along with different thermodynamic parameters related to the crystallographic phase transition evaluated from the five trials (Fig. 4). (N.B. the preceding results from high-pressure d*Q*/|d*T*| measurements and caloric effects evaluated are from Trial 2.) Calorimetry indicates that |*Q*_0_| and |∆*S*_0_| (normalised by total sample mass now for simplicity) associated with the first-order phase transition are nominally similar over Trials 1−3 (up to ~7 months). These values decrease by ~70% after 17 months and we note that our samples were stored at ambient temperature and pressure in vials that were not perfectly sealed during that period. The large decrease in |*Q*_0_| and |∆*S*_0_|, which are extensive quantities, after 17 months could be attributed to further adsorption of atmospheric nitrogen, which has been shown to occur even at ambient conditions^46^, and to further conversion of nitrogen-loaded ZIF-4 to ZIF-*zni* after repeated exposure to nitrogen at high pressures. Nevertheless, d*T*_0_/d*p*, which is an intensive thermodynamic quantity, decreased rather less by ~10% (from ~76 to 67 K kbar^−1^) after 20 months. The small decrease in d*T*_0_/d*p* could be an indication that the transition Δ*V*_0_ also slightly decreases with increased nitrogen loading into ZIF-4 pores, which may hinder the rotation of its imidazole linkers^34^. Thus, while our sample specimen appears to show a reduction in |∆*S*_0_| normalised by total mass, this is consistent with an increase in inactive mass over time.

Also, it can be noted from Fig. 4a,b that the *T*_0_ of nitrogen-loaded ZIF-4(Zn) at a given value of applied *p* varies up to ~20 K between different trials in an apparently stochastic manner, but the d*T*_0_/d*p* of each individual trial is monotonic. Such stochasticity is a thermodynamic feature common for first-order phase transitions, which is observed to less extent in other barocaloric materials, and is likely a consequence of different amounts of adsorbed nitrogen stabilising the LT phase to varying degrees. This is supported by previous works that have reported that the penetration of PTF into MOF pores influences the transition energy barrier when driving crystallographic phase transitions with applied hydrostatic pressure^43,50,51^.

**Fig. 5:**
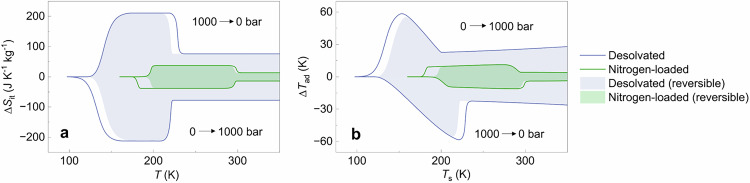
Barocaloric effects in desolvated and nitrogen-loaded ZIF-4(Zn). **a** Isothermal entropy changes ∆*S*_it_ and (**b**) adiabatic temperature changes ∆*T*_ad_ estimated for desolvated ZIF-4(Zn) and evaluated for nitrogen-loaded ZIF-4(Zn) driven on applying and removing pressure *p* = 1000 bar near the phase transition. Shaded regions represent the reversible barocaloric effects.

We can also estimate the barocaloric effect in desolvated ZIF-4(Zn) using our calorimetric data from initial ambient-pressure measurements, volumetric data from LT-XRD on desolvated ZIF-4(Zn), the relevant Clausius-Clapeyron relation, and isobaric ∆*S*(*T*) functions of the nitrogen-loaded sample with a similar mass-normalisation correction performed as before (details in Methods section). This yields maximum barocaloric parameters of |∆*S*_it_| ~ 210 J K^−1^ kg^−1^ and |∆*T*_ad_| ~ 60 K driven by |∆*p*| = 1 kbar near *T*_0_ ~137 K over a wide temperature range, reversibly spanning >80 K, when all crystals of a pure sample mass of desolvated ZIF-4(Zn) transform between the LT and HT phases (Fig. 5). This could be achieved by using an appropriate large-molecule PTF like silicone oil (AP 100) to drive the transition in desolvated ZIF-4(Zn), which has been shown to not transform to ZIF-*zni* when subjected to pressures up to 4 kbar^44^. This barocaloric effect could potentially be tuned to higher operating temperatures by using small amounts of adsorbed nitrogen as a tuning guest molecule in ZIF-4(Zn), which can then be driven in cooling systems using a large-molecule PTF, such as the hydraulic oils used in barocaloric prototypes.

### Direct measurement of the barocaloric effect

**Fig. 6:**
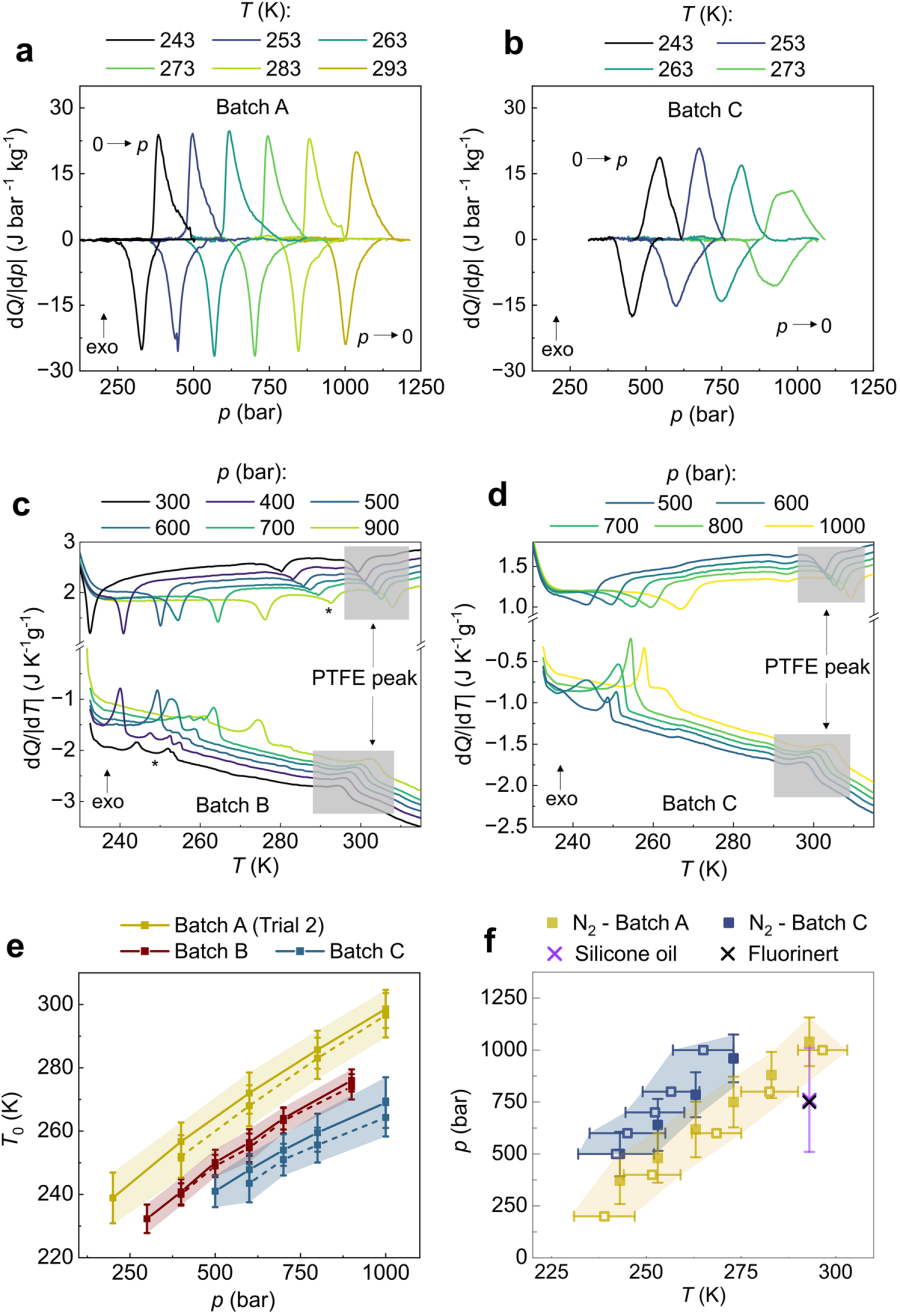
Comparison of the first-order phase transition driven isothermally and isobarically in nitrogen-loaded ZIF-4(Zn) from different synthesized batches. **a**, **b** Isothermal calorimetry on applying (0 → *p*) and removing (*p* → 0) pressure *p* using nitrogen gas at different values of temperature *T*, showing direct measurement of the barocaloric effect in nitrogen-loaded ZIF-4(Zn) in **a** Batch A and **b** Batch C, normalised by total sample mass. **c**, **d** Isobaric calorimetry on nitrogen-loaded ZIF-4(Zn) from (**c**) Batch B and (**d**) Batch C at different values of *p*. Grey shaded areas represent peaks from a polytetrafluoroethylene (PTFE) sealant used within the calorimeter sample cell. **e** Comparison of *T*_0_ values as a function of *p* from isobaric calorimetry on heating (solid lines) and cooling (dotted lines) between Batch A (Trial 2), Batch B and Batch C. The multiple peaks in Batch B denoted by asterisks in (**d**) are unaccounted for, and the *T*_0_ at different values of *p* reported in (**e**) are *T* values of the main peak apex. For Batches A and C, *T*_0_ values reported in (**e**) are the midpoint temperatures of the ∆*S*(*T*) functions associated with the transitions reported in Fig. 1d and Fig. 6d, respectively. **f** Transition regimes in *T-p* space for nitrogen-loaded ZIF-4(Zn) (squares) from Batch A Trial 2 (yellow) and Batch C (blue) evaluated from isothermal (solid squares) and isobaric (empty squares) calorimetry. Literature values of *p*_0_ reported for driving the transition in desolvated ZIF-4(Zn) (crosses) using Fluorinert FC-70 (black) and silicone oil (purple) are included for comparison. All bars in (**e**) and (**f**) indicate the widths of the transition from onset to completion. Shaded yellow, red and blue regions in (**e**) and (**f**) clarify transition regimes in *T-p* space for Batch A, Batch B and Batch C, respectively. Exothermic (exo) peaks are defined with upward arrows for the calorimetry data in (**a**–**d**).

Isothermal calorimetry was performed under changes in applied hydrostatic pressure using nitrogen gas to verify and directly probe the barocaloric effect in nitrogen-loaded ZIF-4(Zn) following Trial 4 of isobaric measurements (Fig. 6a). Integration of isothermal calorimetric peaks yields the isothermal heat *Q*_it_ associated with the first-order transition driven by pressure, given by *Q*_it_(*T*, 0→*p*) = ${\int }_{0}^{p}{({{\rm{d}}}Q / |{{\rm{d}}}p{\hbox{'}}{|})}_{T}{{\rm{d}}}p{\hbox{'}}$. The values of isothermal heat |*Q*_it_| ~ 1.4 ± 0.1 kJ kg^−1^ obtained from these direct measurements are in good agreement with the heat of transition evaluated during the preceding isobaric calorimetric measurements (*|Q*_0_| ~ 1.3 ± 0.2 kJ kg^−1^) from the quasi-direct method when normalised by total sample mass. These direct measurements indicate a shift d*T*_0_/d*p* ~ 71 K kbar^−1^, which is again in good agreement with the d*T*_0_/d*p* ~ 69 K kbar^−1^ evaluated from Trial 4 of quasi-direct measurements, thereby confirming reproducibility from direct changes in pressure.

### Effect of particle size on the first-order transition in ZIF-4(Zn)

Particle size is known to influence MOF structural transitions, adsorption properties and phase stability^32,52–54^. The first-order crystallographic transition in desolvated ZIF-4(Zn) was reported to be observed only in powdered ZIF-4(Zn), whereas larger single crystals (with dimensions >260 μm) showed no such transition^34^. Another report confirmed^30^ no such transition in ZIF-4(Zn) crystals with an average particle size of 625 ± 137 μm. To test for reproducibility of the barocaloric effect in ZIF-4(Zn), multiple batches of desolvated ZIF-4(Zn) powder samples were synthesised (Batches A–E) using the same solvothermal method with slight differences in the synthesis protocols (Supplementary Table 2) and imaged using scanning electron microscopy (SEM) to evaluate particle sizes (Supplementary Fig. 13). (N.B. All calorimetric results reported in preceding subsections are from Batch A). These images indicate agglomeration of a vast majority of particles with dimensions <10 μm in Batches A–C. In contrast, Batches D and E contain less agglomerated particles with much larger particle size on average, well above 20 μm.

When the first-order phase transition and barocaloric effects were probed in all batches, only Batches A–C showed response. From calorimetry at ambient pressure, entropy changes associated with the phase transition evaluated in these batches ranged from |∆*S*_0_| ~ 125 to 225 J K^−1^ kg^−1^ when normalised by total sample mass (Supplementary Fig. 14). The barocaloric effect in Batch C was evaluated directly using isothermal calorimetry (Fig. 6b), and isobaric calorimetric data for Batches B and C also showed *T*_0_ shifting under applied pressure (Fig. 6c, d). The barocaloric entropy changes evaluated for Batches B and C were |∆*S*_it_| ~ 25 J K^−1^ kg^−1^ (when normalised by total sample mass), once again signifying deactivation after pressurisation under nitrogen, whilst d*T*_0_/d*p* ranged from 60 to 76 K kbar^−1^ (Fig. 6e). As with the d*T*_0_/d*p* curves from the different trials on Batch A, the d*T*_0_/d*p* curves for the different batches are monotonic, while *T*_0_ at a given value of applied *p* varies up to ~35 K between different batches, further highlighting the dynamic nature of the transition in ZIF-4(Zn). Figure 6f shows transition regimes in *T-p* space for nitrogen-loaded ZIF-4(Zn) from Batch A and Batch C evaluated from isothermal and isobaric calorimetry. From correlation of transition temperatures *T*_0_ and pressures *p*_0_ in *T*-*p* space (Fig. 6f), and latent heats evaluated using isobaric and isothermal calorimetry, it can be established that the transitions observed from our direct and quasi-direct analysis of Batch C are identical. Therefore, the splitting transition peaks on cooling in each isobar in Fig. 6d likely indicate different degrees of nitrogen adsorption within the crystals. For comparison, Fig. 6f shows the reported literature values of *p*_0_ ~ 750 bar when driving this crystallographic transition in desolvated ZIF-4(Zn) using different PTFs [silicone oil^44^ (AP 100) and Fluorinert^43^ FC-70]. The transition onset using silicone oil as the PTF was reported at ~500 bar, with completion occurring just above ~1000 bar. Our measurements of *p*_0_(*T*) for nitrogen-loaded ZIF-4(Zn) from isothermal measurements are in agreement with these reported values for desolvated ZIF-4(Zn), which indicates that it is the same crystallographic transition being driven.

Conversely, Batches D and E showed no first-order phase transition within the temperature range of 80 K ≤ *T* ≤ 343 K, and thereby no large barocaloric effect. From our SEM images (Supplementary Fig. 13), it can be qualitatively evaluated that the particle sizes of Batches D and E are much larger on average when compared with Batches A–C. While no particle size threshold has been reported for the observation of the first-order phase transition in ZIF-4(Zn), it can be estimated from this work that the particle dimensions should likely be at least <10 μm in order to undergo the transition, a finding that is in agreement with literature reports^30,34^.

## Discussion

The tunability of operational temperatures using sorption of guest molecules has not hitherto been demonstrated in barocalorics, and should be generalisable to a myriad of porous MOF and fluid combinations, given the reports of varying transition energy barriers with partial penetration of different PTFs into MOF pores^43,50,51^. Precision loading of small amounts of nitrogen adsorbent in to ZIF-4 pores, along with careful tailored synthesis protocols to produce small homogenous particle sizes would be important for applications. Such controlled incorporation of nitrogen into ZIF-4(Zn) along with the use of an appropriate large-molecule PTF should tailor the transition temperature between 137 K < *T*_0_ < 190 K to yield barocaloric entropy changes up to |∆*S*_it_| ~ 210 J K^−1^ kg^−1^ that can be driven over wide temperature spans larger than 80 K using |∆*p*| = 1 kbar, which is beneficial to cooling systems that require operational temperatures below room temperature. For instance, such tuneable thermodynamic properties can be exploited in a barocaloric cooling prototype, wherein increasingly guest-loaded active material is successively packed along the length of a column. Adiabatic application and removal of hydrostatic pressure within this column will then generate a large, cascading temperature gradient across the column, that would maximise the difference between the hot (*T*_h_) and cold (*T*_c_) temperatures of the heat pump, which would thereby enhance device cooling performance. This cascading effect draws similarity in concept^38^ to the magnetocaloric effect in hydrogenated compounds of La(Fe_0.88_Si_0.12_)_13_H_*y*_, which is extended over a wide temperature span as a result of varying *y*, but with stable and larger thermal changes for our MOFs.

To achieve this, our findings should motivate focus on controlled synthesis and guest-loading in ZIF-4(Zn) to target specific adsorption volumes and hence finely control barocaloric performance using guest molecules as a caloric vector. This work should also encourage the search for other MOFs with large transition |Δ*S*_0_| and |Δ*V*_0_|/*V* values which could produce giant barocaloric effects at starting temperatures that can be tailored by the controlled incorporation of guest molecules and the use of different PTFs in the search for practical replacements for HFCs in conventional cooling and heating technologies.

## Methods

### Synthesis

Desolvated powdered ZIF-4(Zn) was synthesised according to a previously outlined procedure^34^ with slight variations in certain steps between different batches as outlined in Supplementary Table 2. In general, 1.2 g of powdered Zn(NO_3_)_2_·6H_2_O and 0.9 g of imidazole were dissolved in 90 ml of DMF and tightly sealed in a glass screw-top jar, and heated in an oven at 373 K over different lengths of time. The solution was then retrieved from the oven and cooled to room temperature. The powder was vacuum filtered from the solvent and washed first with few drops of DMF, followed by a few drops of dichloromethane (DCM). The powder was then stirred in ~100 ml DCM for different lengths of time between different batches (Supplementary Table 2), and vacuum filtered again before desolvating in a vacuum oven to obtain desolvated ZIF-4(Zn).

### Characterisation

Powder-XRD was performed using a Bruker D8 Advance diffractometer in Bragg-Brentano reflection geometry using Cu Kα radiation. For room temperature measurements, ZIF-4(Zn) powder was loaded on to a front-loading single crystal silicon sample holder. For LT-XRD measurements, a thin layer of powder was adhered to a flat chromium-plated copper sample mount using a little vacuum grease, and then placed on an Oxford Cryosystems PheniX low-temperature stage and brought under vacuum to about 10^−4^ mbar. A thermalisation time of 10 minutes was allowed after ramping temperatures at 0.05 K s^−1^ before obtaining diffraction patterns.

The synthesised ZIF-4(Zn) powder samples were imaged using a FEI Nova NanoSEM 450 microscope with a field emission gun operating at a 5 kV accelerating voltage and an Everhart-Thornley secondary electron detector. An electron beam diameter of 2.5 nm was used. Powdered ZIF-4(Zn) was adhered onto an aluminium stub using aluminium tape. The powdered specimen was sputter-coated with a thin layer of palladium (<10 nm thickness) using an Emitech K575 sputter coater prior to imaging to reduce charging. Particle sizes were manually analysed by measuring projected area diameters using a length measuring tool on ImageJ.

### Calorimetry

Ambient-pressure calorimetry was performed using a Q2000 DSC from TA Instruments and a bespoke calorimeter designed and built within our group, based on a previously reported system^55^, to obtain calorimetric measurements down to ~90 K using a liquid nitrogen thermal bath.

Specific heat capacity (*c*_*p*_) of ZIF-4(Zn) was measured at atmospheric pressure using the Q2000 DSC between 97 K < *T* < 360 K at a temperature-ramping rate of |d*T*|/d*t* = 5 K min^−1^ using a sample mass ~7 mg. Calorimetric measurements (d*Q*/|d*T*|) for specific heat capacity were calibrated using the calorimetric response of an empty sample pan and a sapphire reference mass run under identical conditions.

High-pressure calorimetry was performed using a Setaram μDSC7 Evo, with high-purity nitrogen gas (99.9999%) as the hydrostatic pressure transmitting fluid. Isobaric measurements of (d*Q*/d*t*)_*p*_ were performed within a temperature range 228 K ≤ *T* ≤ 343 K (228 K being the low-temperature limit of the instrument) at |d*T*|/d*t* = 1.2 K min^−1^. For direct measurements of caloric effects from isothermal calorimetry, (d*Q*/d*t*)_*T*_ measurements were performed using a pressure-ramping rate of |d*p*|/d*t* = 13 bar min^−1^, which was slow enough to ensure isothermal conditions. The final d*Q*/|d*p*|_*T*_ measurements reported were obtained by normalising (d*Q*/d*t*)_*T*_ by pressure-ramping rate |d*p*|/d*t* and total sample mass.

To determine the effect of nitrogen exposure on ZIF-4(Zn), two separate ~15 mg sample specimens of pristine, desolvated ZIF-4(Zn) from Batch A were loaded into the sample cell of the Setaram μDSC7 Evo, brought down to *T* = 233 K and exposed to nitrogen gas at set applied pressure *p* ~ 1000 bar for 9 and 12 h. After the exposure, the temperature was brought back up to 293 K, the applied pressure released and the samples transferred out of the HP-DSC to small aluminium crucibles to be run in the bespoke calorimeter to evaluate the ambient-pressure calorimetric signal after nitrogen exposure.

### Evaluation of barocaloric effects

For nitrogen-loaded ZIF-4(Zn), plots of entropy as a function of temperature and pressure were constructed with respect to the entropy at *p* = 0 bar and *T* = 160 K (a temperature below the crystallographic transition of nitrogen-loaded ZIF-4(Zn) selected arbitrarily) using *S*′(*T*, *p*)  =  *S*(*T*, *p*) − *S*(160 K,0) according to:

1 $$S^{\prime} (T,\, p)=\left\{\begin{array}{c}{\int }_{160\;{{\rm{K}}}}^{{T}}\frac{{c}_{{{\rm{LT}}}}\left({T}^{{\prime} }\right)}{{T}^{{\prime} }}{{{\rm{d}}}}{T}^{{\prime} }\hfill\,T\le {T}_{1}\\ S\left({T}_{1},\, p\right)+{\int }_{{T}_{1}}^{T}\frac{1}{{T}^{{\prime} }}\left({c}_{{{\rm{LT}}}-{{\rm{HT}}}}\left({T}^{{\prime} }\right)+\left|\frac{{{{\rm{d}}}}Q\left({T}^{{\prime} },\, p\right)}{{{{\rm{d}}}}{T}^{{\prime} }}\right|\right){{{\rm{d}}}}{T}^{{\prime} }\,\,\,\,\,{T}_{1}\le T\le {T}_{2}\\ S\left({T}_{2},\, p\right)+{\int }_{{T}_{2}}^{T}\frac{{c}_{{{\rm{HT}}}}\left({T}^{{\prime} }\right)}{{T}^{{\prime} }}{{\rm{d}}}{T}^{{\prime} }\hfill\,\,\,T\ge {T}_{2}\,\end{array}\right.$$

where *c*_LT_ and *c*_HT_ are the specific heat capacities of the LT and HT phases, respectively, *c*_LT-HT_ is the specific heat capacity within the phase transition regime (analysed by fitting a sigmoidal curve below the calorimetric transition peak to *c*_LT-HT_ = (1 − *x*) *c*_LT_ + *x c*_HT_, *T*_1_ and *T*_2_ are the start and finish temperatures of the transition and *T’* is the dummy variable of integration. In the ${T}_{1}\le T\le {T}_{2}$ regime, the functions of $\frac{1}{T{\prime} }\left|\frac{{{{\rm{d}}}}Q\left({T}^{{\prime}},\, p\right)}{{{{\rm{d}}}}{T}^{{\prime} }}\right|$ within the integral (which represent isobaric |Δ*S*|(*T*) functions associated with the first-order transition) have been normalised by 42% of the total sample mass to account for the active mass. Isothermal entropy changes [Δ*S*_it_(*T, p*)] and adiabatic temperature changes [Δ*T*_ad_(*T, p*)] driven by changes in pressure, Δ*p* ~ *p*, were evaluated by following isothermal and adiabatic trajectories, respectively, on the *S*’(*T*, *p*) plots.

For desolvated ZIF-4(Zn), the barocaloric effect was estimated for |Δ*p*| = 1 kbar using isobars of *S*’-*T* curves constructed for *p* = 0 bar and *p* = 1000 bar. The *S*’-*T* isobar for *p* = 0 bar was constructed using the isobaric |Δ*S*|(*T*) function evaluated from calorimetric data obtained before high-pressure measurements (Fig. 1b) along with specific heat capacity data. The *S*’-*T* isobar for *p* = 1000 bar was constructed by using the isobaric |Δ*S*|(*T*) function of nitrogen-loaded ZIF-4(Zn) at *p* = 1000 bar as a template, but correcting for the magnitude of |Δ*S*_0_| and the onset of *T*_0_ using our LT-XRD data and the temperature-pressure Clausius-Clapeyron relation. By taking the ambient-pressure |Δ*S*_0_| ~ 212 J K^−1^ kg^−1^ normalised by active mass before high-pressure measurements, an equivalent active mass of ~8% of the total sample mass is evaluated from the ambient-pressure |Δ*S*_0_| ~ 16 J K^−1^ kg^−1^ of nitrogen-loaded ZIF-4(Zn), if we assume that the crystals have not adsorbed any nitrogen and the total sample mass transforms between the LT and HT phases with the full transition |∆*V*_0_|/*V* ~ 22% volume change, as has been previously reported^44^. This is used to renormalise the isobaric |Δ*S*|(*T*) function of nitrogen-loaded ZIF-4(Zn) at *p* = 1000 bar. From d*T*_0_/d*p* ~ 86 K kbar^−1^ evaluated using the temperature-pressure Clausius-Clapeyron relation for desolvated ZIF-4(Zn) with volume data from LT-XRD patterns and calorimetry data, we shift the |Δ*S*|(*T*) function at *p* = 1000 bar along the *T* axis to satisfy *T*_0(*p* = 1000 bar)_ ≈ *T*_0(*p* = 0 bar)_ + 86 K. The barocaloric Δ*S*_it_(*T*, *p*) and Δ*T*_ad_(*T*, *p*) driven by |Δ*p*| = 1000 bar are then estimated by following isothermal and adiabatic trajectories, respectively, on the final *S*’(*T*, *p*) plots.

## Supplementary information

Supplementary Information

Transparent Peer Review file

## Data Availability

The calorimetry, X-ray diffraction and scanning electron microscopy imaging data generated in this study have been deposited in the University of Cambridge Repository Apollo and are available at 10.17863/CAM.121982.2.
