# Supplementary material for: Giant tunable barocaloric effects using guest-molecule adsorption in a porous metal-organic framework
Source: Nat Commun. 2026 Jun 26;17:8077. doi: 10.1038/s41467-026-74616-6 (PMC13454139; doi:10.1038/s41467-026-74616-6)
Supplement: Supplementary file 1 [file 41467_2026_74616_MOESM1_ESM.pdf]

Supplementary Information

Giant tunable barocaloric effects using guest-molecule adsorption in a porous metal-organic framework

Melony Dilshad[a*], Giulio I. Lampronti[a], Thomas D. Bennett[b] & Xavier Moya[a*]

[a] Department of Materials Science & Metallurgy, University of Cambridge, Cambridge, CB3 0FS, UK.

[b] MacDiarmid Institute for Advanced Materials and Nanotechnology, School of Physical and Chemical Sciences, University of Canterbury, Private Bag 4800, Christchurch 8140, New Zealand.

*Corresponding authors: Melony Dilshad (md712@cam.ac.uk) and Xavier Moya (xm212@cam.ac.uk)

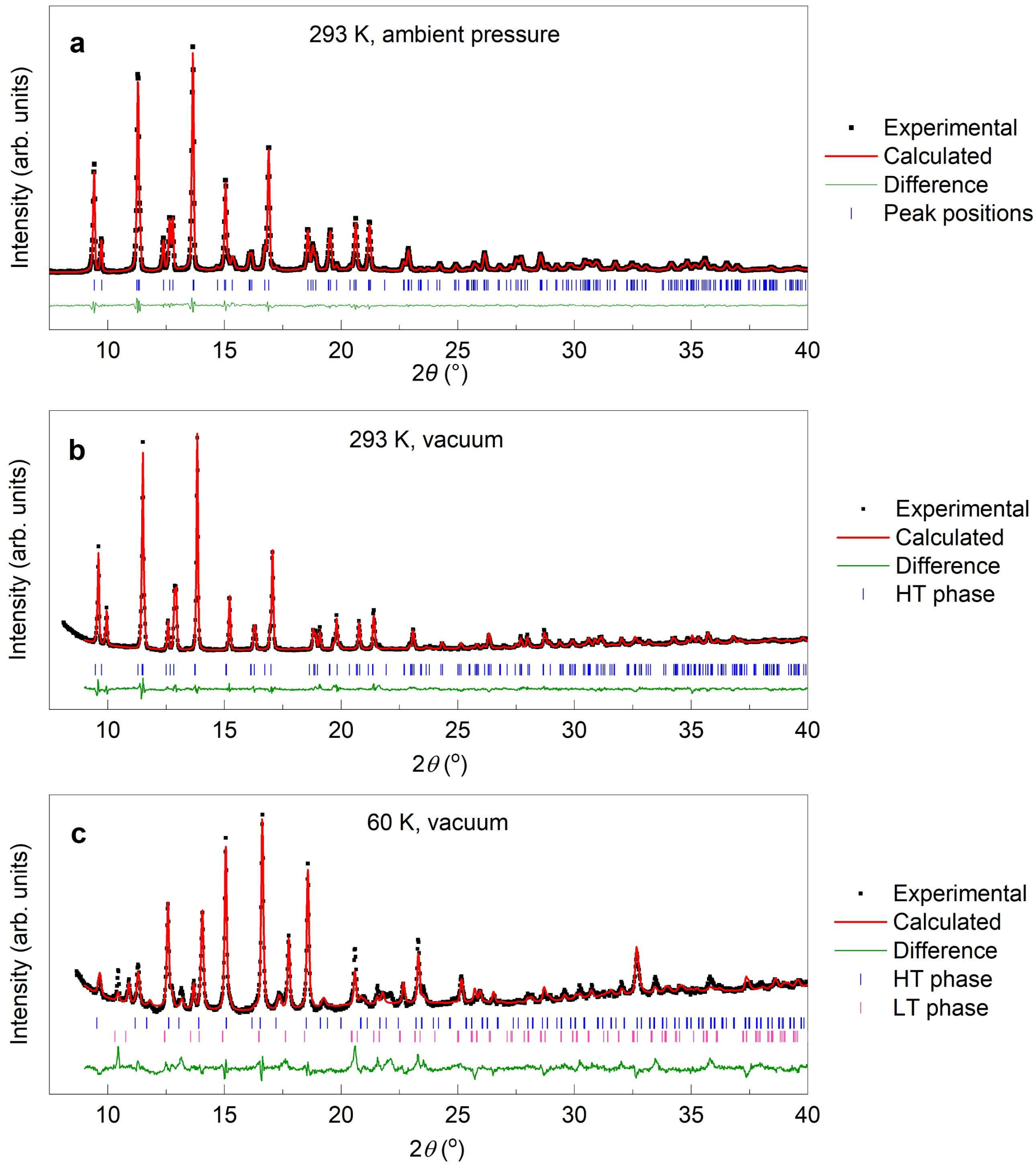

Supplementary Fig. 1. X-ray diffraction (XRD) patterns of desolvated ZIF-4(Zn) powder (*Batch A*) before high-pressure DSC measurements. **a** Pawley fit of the XRD pattern obtained at ambient temperature and pressure, fitted to an orthorhombic *Pbca* structure. **b,c** Rietveld refinements of the XRD patterns obtained using the LT-XRD stage under vacuum at **a** 293 K and **b** 60 K.

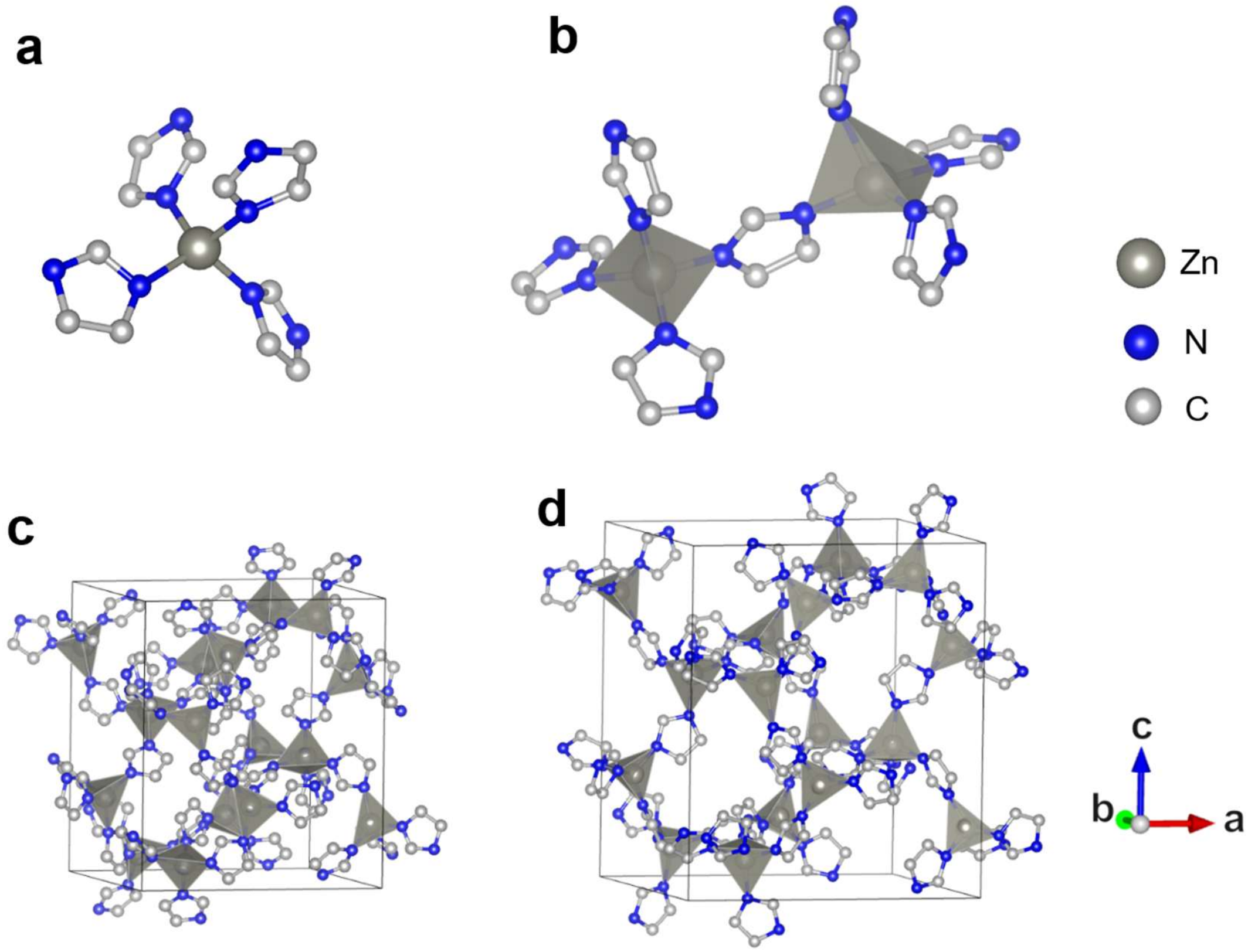

Supplementary Fig. 2. Crystal structures of desolvated ZIF-4(Zn) (hydrogen atoms are omitted for clarity). **a** Structure of a basic building unit of ZIF-4(Zn) showing a zinc (Zn^{2+}) center bound by imidazolate ions $(C_3H_3N_2)^-$. **b** Polyhedra showing tetrahedral coordination of four imidazolate ligands to a zinc metallic center, with two zinc centers bridged by an imidazolate ligand. Unit cells of the **c** LT phase and **d** HT phase of desolvated ZIF-4(Zn). The HT phase is 22% larger in volume than the LT phase, with larger pore space.

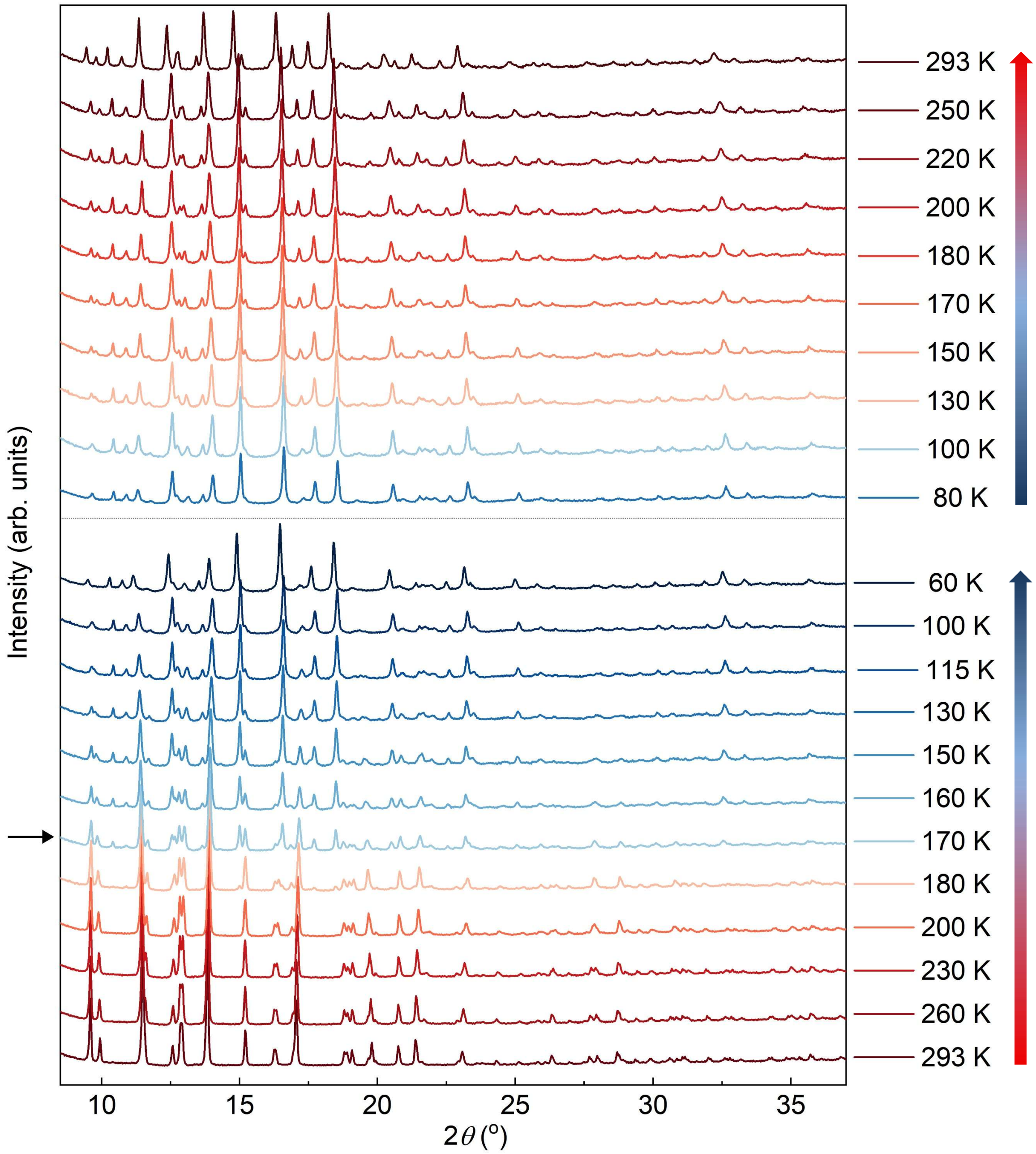

Supplementary Fig. 3. Stacked plot of LT-XRD patterns of desolvated ZIF-4(Zn) *Batch A* on cooling (lower plot) and heating (upper plot) before high-pressure DSC measurements, starting at 293 K (bottom-most pattern), cooled down to 60 K, and heated back up to 293 K (indicated by upward arrows on the right). The black arrow on the left shows the pattern at which the first peaks of the LT phase visibly begin to appear on cooling. Patterns were obtained for 20 values of temperature each on cooling and heating. However, a few intermediate patterns have been left out in this compilation to be concise. The full set of raw data on cooling are available in the University of Cambridge repository Apollo[1].

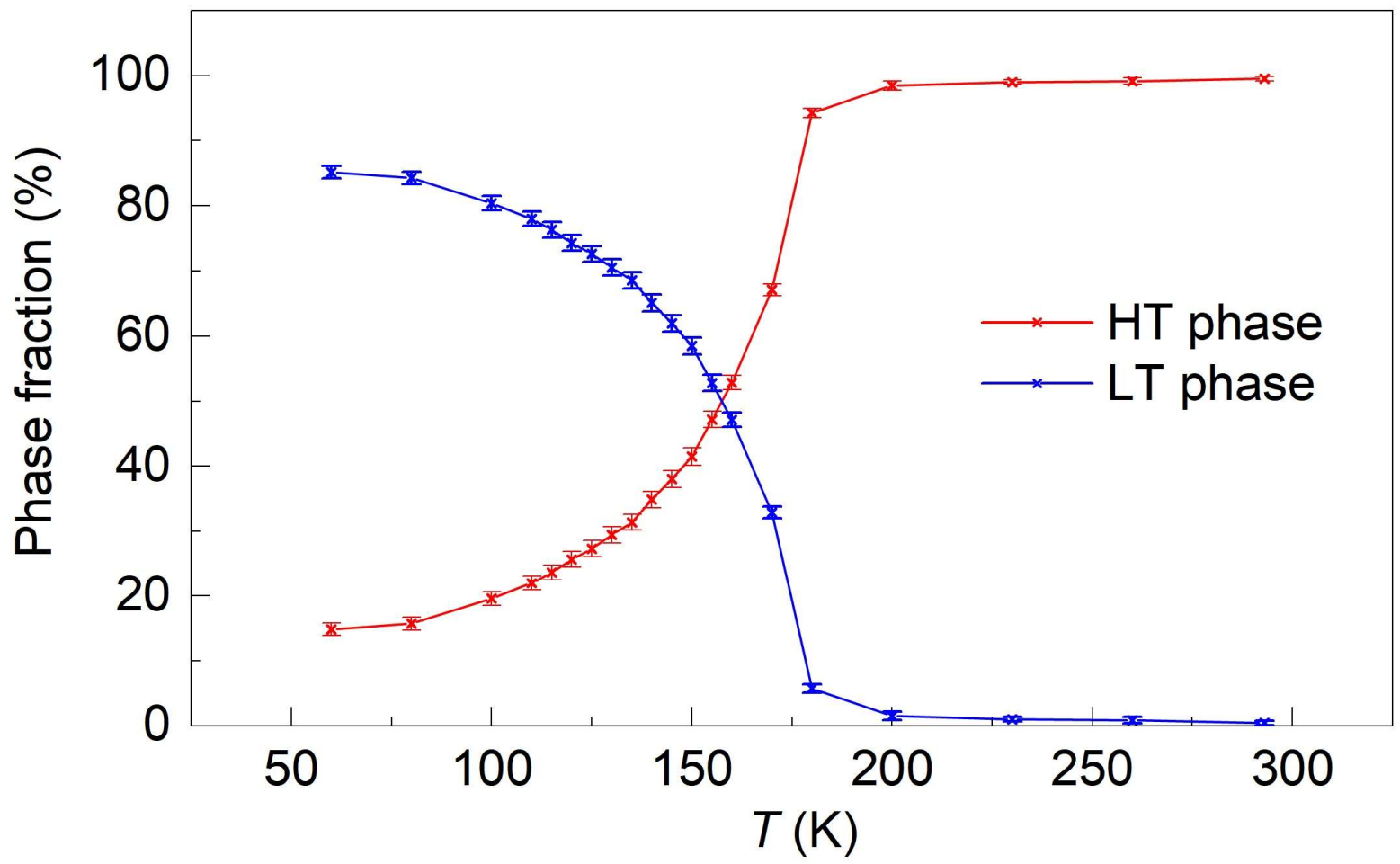

Supplementary Fig. 4. Phase fractions of the LT and HT phases of desolvated ZIF-4(Zn) as a function of temperature evaluated from Rietveld refinements of all XRD patterns obtained on cooling. The error bars denote one estimated standard deviation.

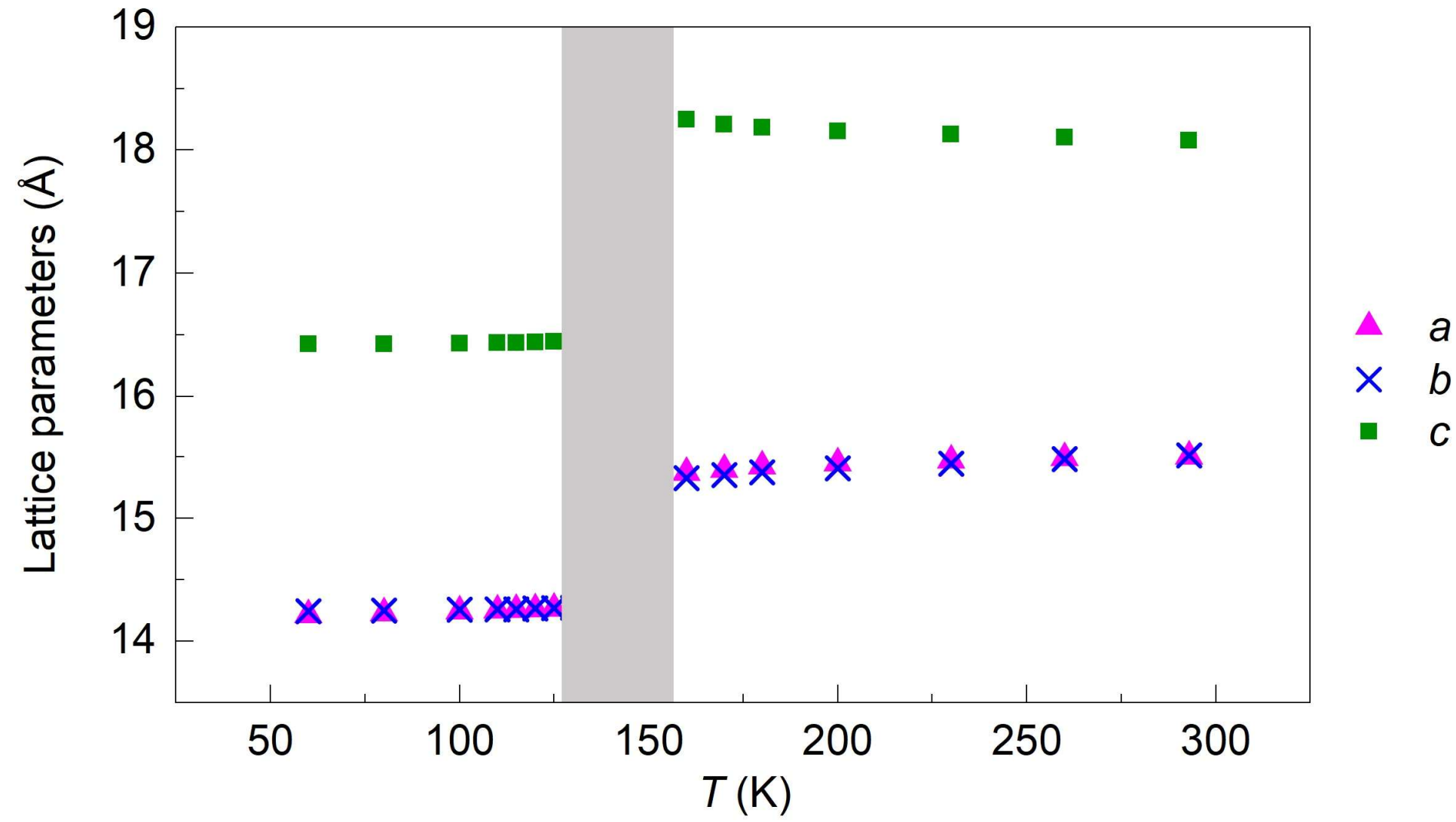

Supplementary Fig. 5. Lattice parameters of desolvated ZIF-4(Zn) analysed from Rietveld refinements of all XRD patterns obtained on cooling. The grey shaded area represents the phase transition regime indicated by calorimetry in Fig. 1a in the main text.

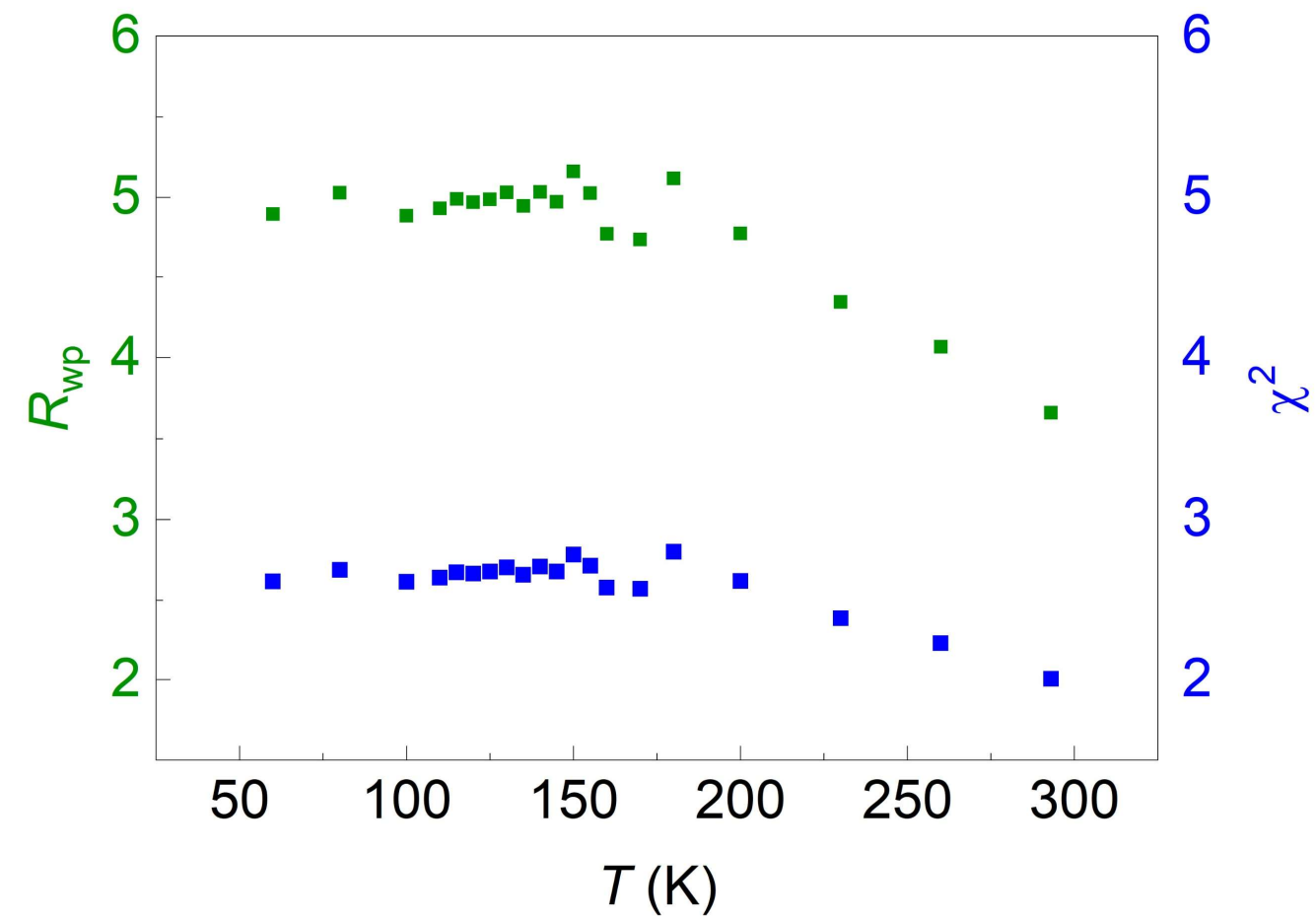

Supplementary Fig. 6. Goodness-of-fit indices (R_{wp} and χ^2) for the sequential Rietveld refinements of all XRD patterns obtained on cooling, showing that the refinement quality is largely consistent across the whole dataset.

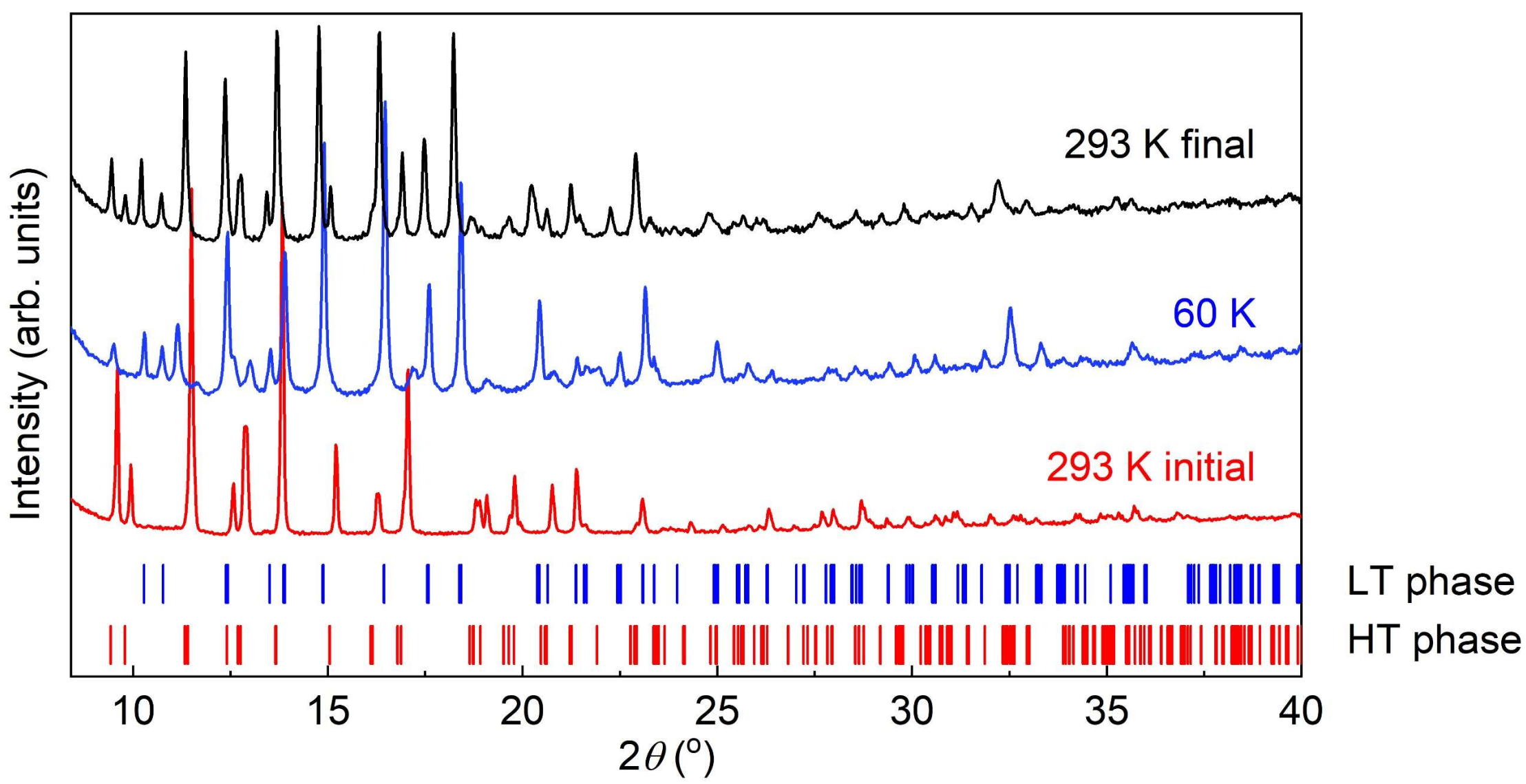

Supplementary Fig. 7. Comparison between XRD patterns of desolvated ZIF-4(Zn) at 293 K at the start (293 K initial) and end (293 K final) of the temperature sequence, and at 60 K. Peak positions of the LT (blue) and HT (red) phases are shown at the bottom.

Supplementary Note 1

XRD pattern refinements for desolvated ZIF-4(Zn)

Supplementary Fig. 3 shows the crystallographic structure evolution of desolvated ZIF-4(Zn) with temperature under vacuum, starting at 293 K. Rietveld refinements were performed on all patterns obtained on cooling. An initial model for the structures of ZIF-4(Zn) HT and ZIF-4(Zn) LT was optimised on a pure phase scan with a rigid body approach. All structural and preferred orientation parameters were then kept fixed during the sequential fit except for lattice parameters. An isotropic Lorentzian term was used to fit the phase contribution to peak broadening. A 12th order Chebyshev polynomial was used to fit the background.

The initial 293 K pattern shows peaks that were indexed purely to the HT orthorhombic phase (Supplementary Fig. 1b), and is identical to the pattern shown in Supplementary Fig. 1a, which was obtained using a different sample stage at ambient pressure. On cooling, new peaks emerge at ~170 K, which were indexed to the reported[2] dense orthorhombic LT structure of ZIF-4(Zn). Phase fraction analysis from Rietveld refinement of the pattern at 60 K indicates that ~15 % of the sample remains in the HT phase after completion of the transition. This is likely due to the presence of large crystals which do not undergo the transition to the LT phase.

On heating back up to room temperature, the patterns still maintain a mix of peaks from both HT and LT phases as shown in the final 293 K pattern, suggesting that not all the crystals that transformed to the LT phase have transformed back to the HT phase. Supplementary Fig. 7 shows a comparison between the initial 293 K pattern, the 60 K pattern and the final 293 K pattern. This partial irreversibility of the volumetric phase transition shown by our LT-XRD patterns is not observed in our DSC measurements (Fig. 1a in the main text), and could be attributed to the significant difference in kinetics between the two measurements. A similar discrepancy was previously reported[2] – a desolvated ZIF-4(Zn) sample was cooled *ex situ* to 77 K in a liquid nitrogen bath for three hours, and after bringing the sample back to room temperature, the XRD patterns consistently showed a mix of LT and HT phases at ambient temperature and pressure for up to 6 weeks after warming up from 77 K. This was in contrast to the DSC measurements reported[2], which showed a reversible transition similar to Fig. 1a in our manuscript. The reason behind this stabilisation of the LT phase at high temperature after remaining at 77 K for three hours is not known, but the difference in transition reversibility was attributed to the difference in measurement kinetics. Nevertheless, the volume change associated with the HT-LT transition in our sample can be evaluated from the XRD patterns of the particles that do transform.

N.B. While our LT-XRD measurements were obtained under vacuum conditions which should stabilise the HT phase, this is less likely to explain the presence of HT phase at 60 K given the partial irreversibility observed on heating during the LT-XRD measurement sequence. A similar series of LT-XRD patterns previously reported[2] showed no trace of HT phase at 80 K when performed under vacuum.

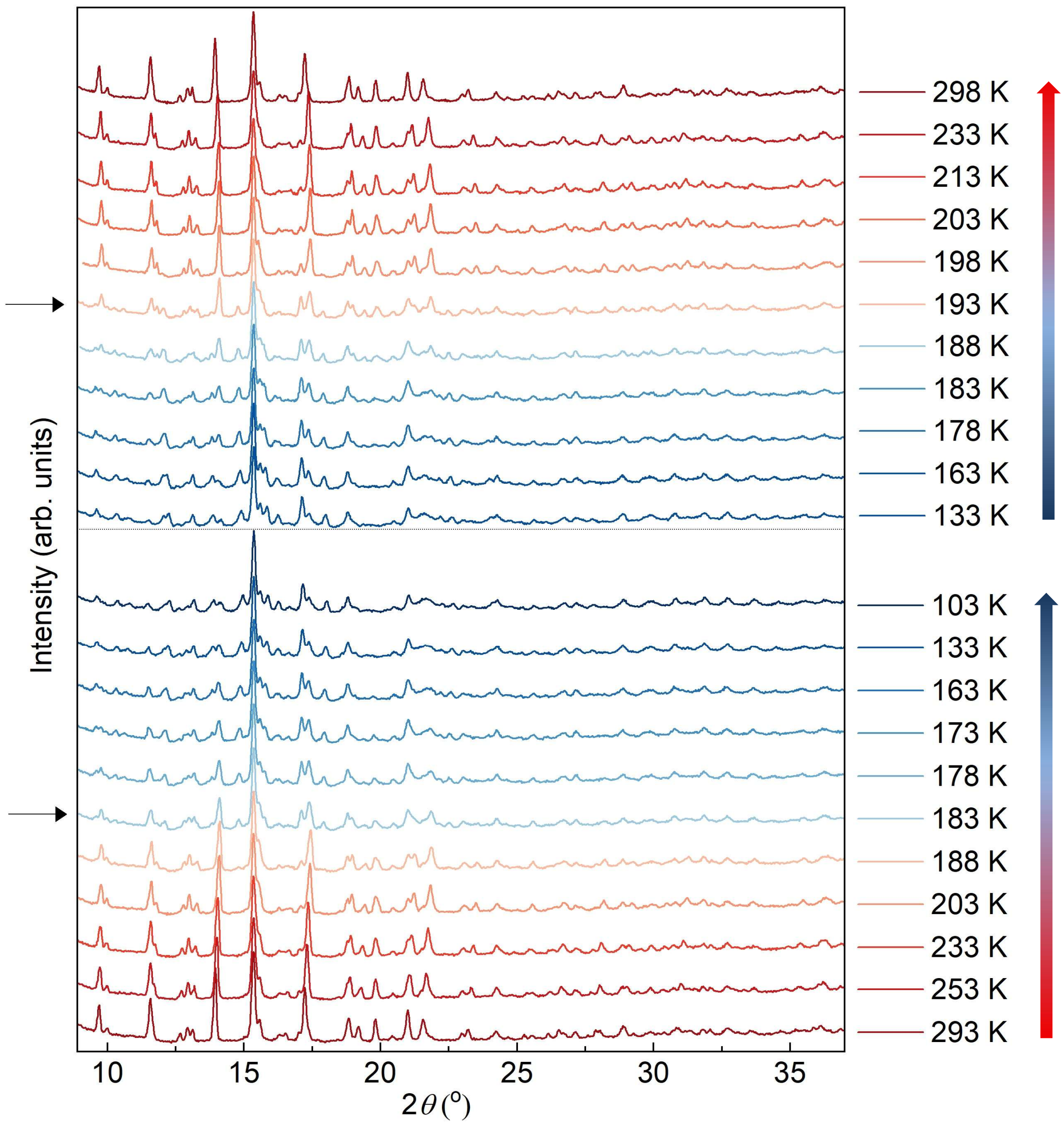

Supplementary Fig. 8. Stacked plot of LT-XRD patterns of nitrogen-loaded ZIF-4(Zn) *Batch A* on cooling (lower plot) and heating (upper plot) after high-pressure DSC measurements using nitrogen gas as PTF, starting at 293 K (bottom-most pattern), cooled down to 103 K, and heated back up to 298 K (indicated by upward arrows on the right). The black arrows on the left indicate the patterns at which the first peaks of the LT phase begin to appear on cooling (bottom arrow), and the first peaks of the HT phase begin to appear on heating (top arrow). Patterns were obtained for 15 values of temperature each on cooling and heating. However, a few intermediate patterns have been left out in this compilation to be concise. The full set of raw data are available in the University of Cambridge repository Apollo[1].

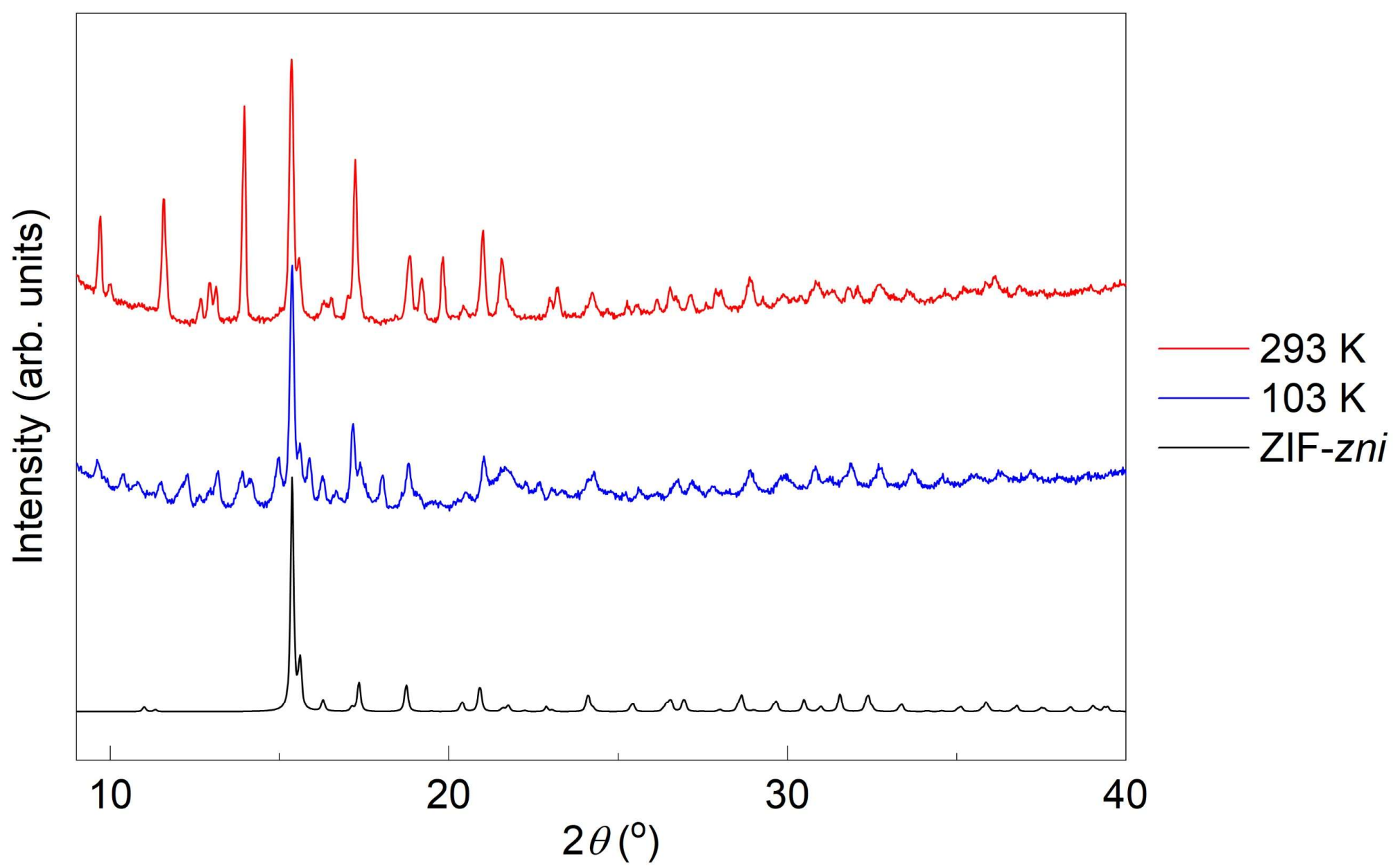

Supplementary Fig. 9. Comparison between XRD patterns of nitrogen-loaded ZIF-4(Zn) at 293 K at the start of the temperature sequence (red) and 103 K (blue). The simulated diffraction pattern of ZIF-*zni* is shown in black.

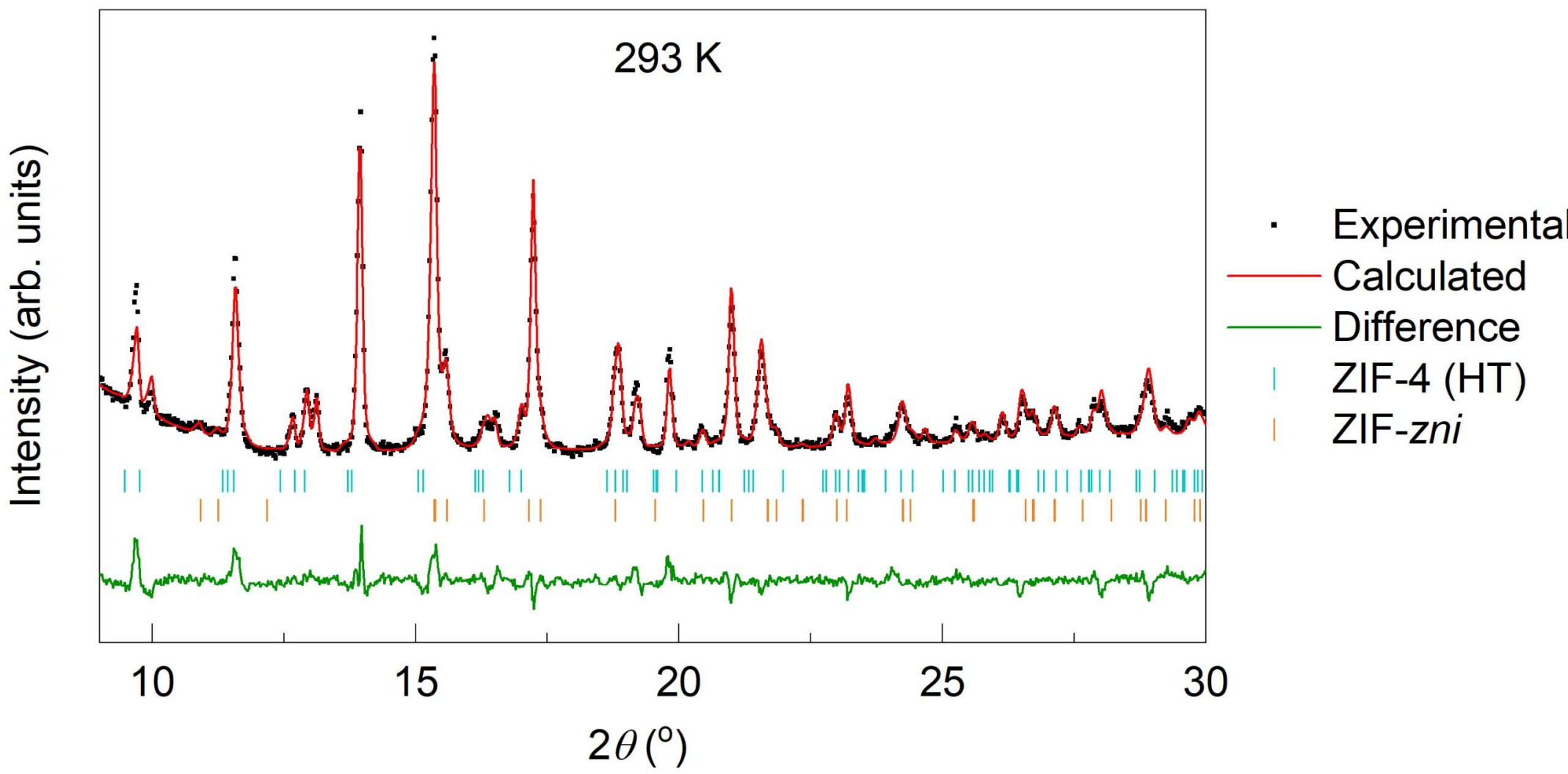

Supplementary Fig. 10. Rietveld refinement of the XRD pattern of nitrogen-loaded ZIF-4(Zn) at 293 K at the start of the temperature sequence.

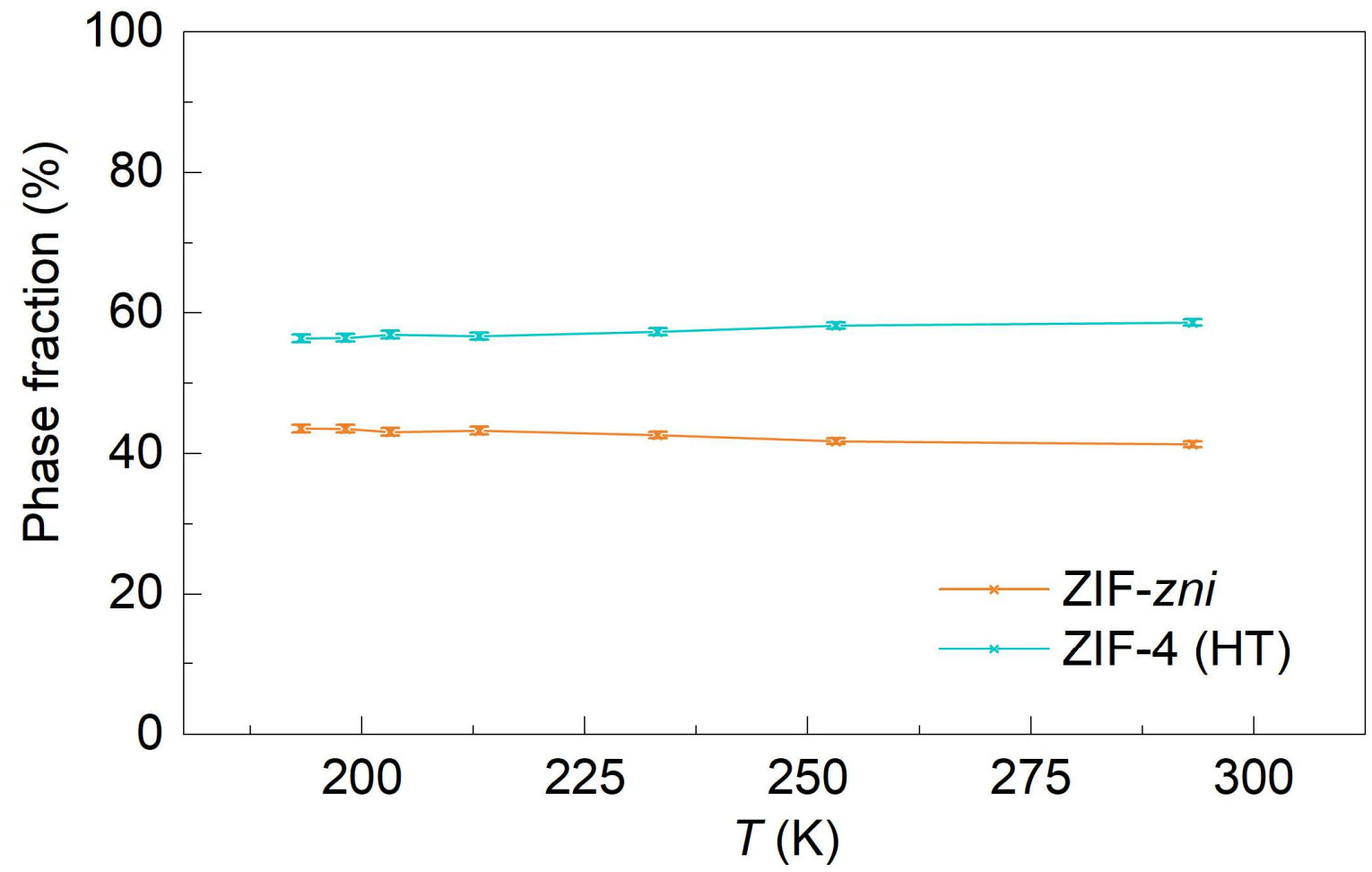

Supplementary Fig. 11. Phase fractions of ZIF-*zni* and the HT phase of (nitrogen-loaded) ZIF-4(Zn) with temperature, evaluated from Rietveld refinements of the XRD patterns obtained on cooling above T_0 from Supplementary Fig. 8 for the ZIF-4(Zn) sample after high-pressure calorimetric measurements using nitrogen gas. The error bars denote one estimated standard deviation.

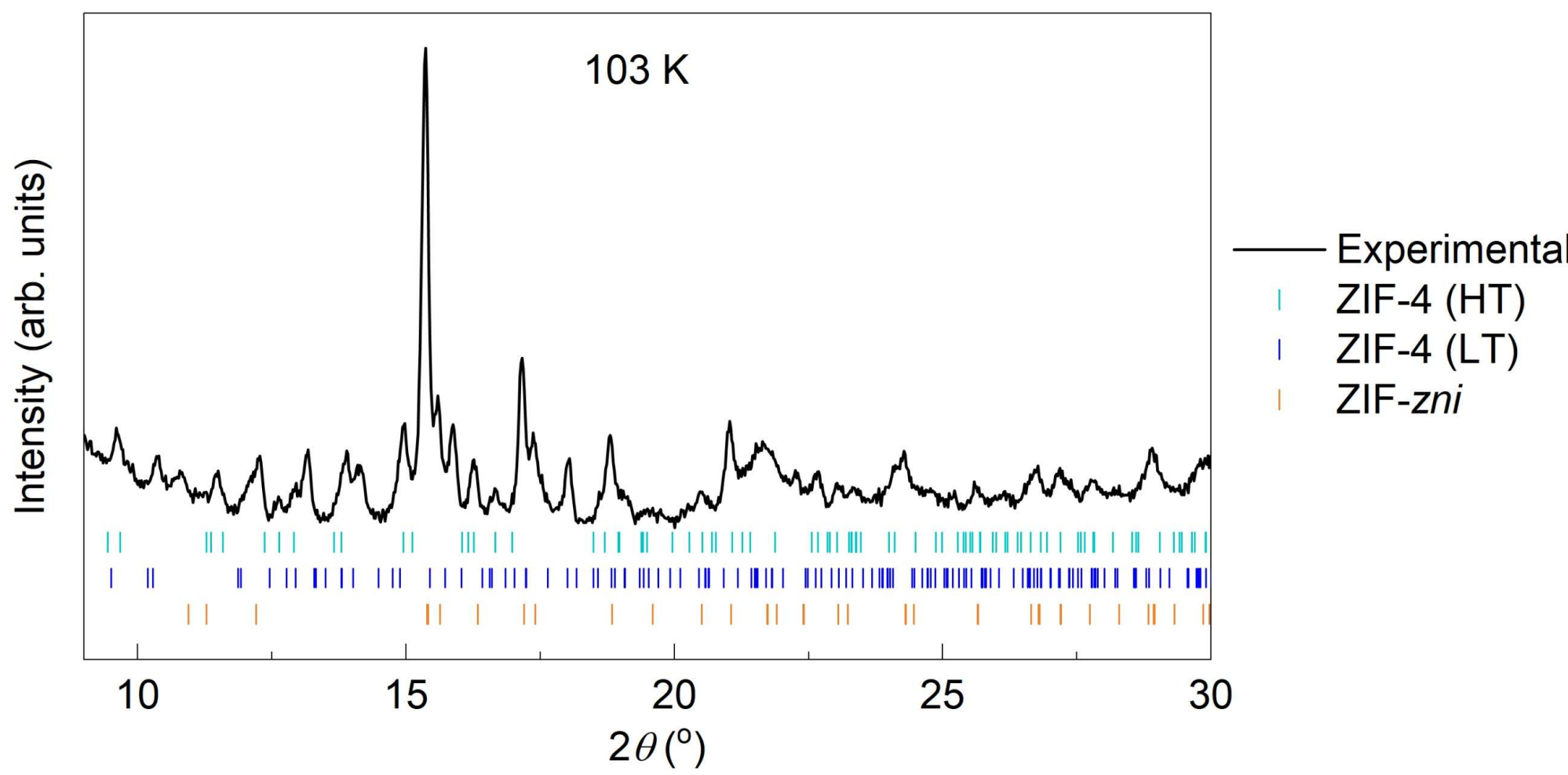

Supplementary Fig. 12. Phase identification for the XRD pattern of nitrogen-loaded ZIF-4(Zn) at 103 K with indicators for Bragg positions of peaks from ZIF-*zni*, ZIF-4(Zn) HT and ZIF-4(Zn) LT structures.

Supplementary Note 2

XRD pattern refinements for nitrogen-loaded ZIF-4(Zn)

Supplementary Fig. 8 shows the crystallographic structure evolution of nitrogen-loaded ZIF-4(Zn) with temperature (under vacuum) from Batch A after high-pressure DSC measurements using nitrogen gas as PTF, starting at 293 K. Rietveld refinements were attempted on all patterns on cooling. An isotropic Lorentzian term was used to fit the phase contribution to peak broadening. A 12th order Chebyshev polynomial was used to fit the background. An initial model for the ZIF-4(Zn) HT and ZIF-*zni* structures was optimised on a pure phase scan with a rigid body approach. All structural and preferred orientation parameters were then kept fixed during the sequential fit except for lattice parameters. For ZIF-*zni* and ZIF-4(Zn) LT phases below 183 K, this task was further complicated by the lack of pure phase patterns. The constraints on the rigid body positions and orientation parameters were particularly hard to avoid in order to refine the model to chemically reasonable minima.

The onset transition temperatures for the phase transition in nitrogen-loaded ZIF-4(Zn) evaluated using these LT-XRD patterns (T_0 ~ 183 K on cooling and T_0 ~ 193 K on heating) corelates well with that evaluated using ambient-pressure DSC. Unlike the patterns for desolvated ZIF-4(Zn), the LT-XRD pattern sequence of the nitrogen-loaded sample on heating indicate that the LT phase has mostly transformed back to the HT phase above T_0. This difference could be a consequence of guest adsorption.

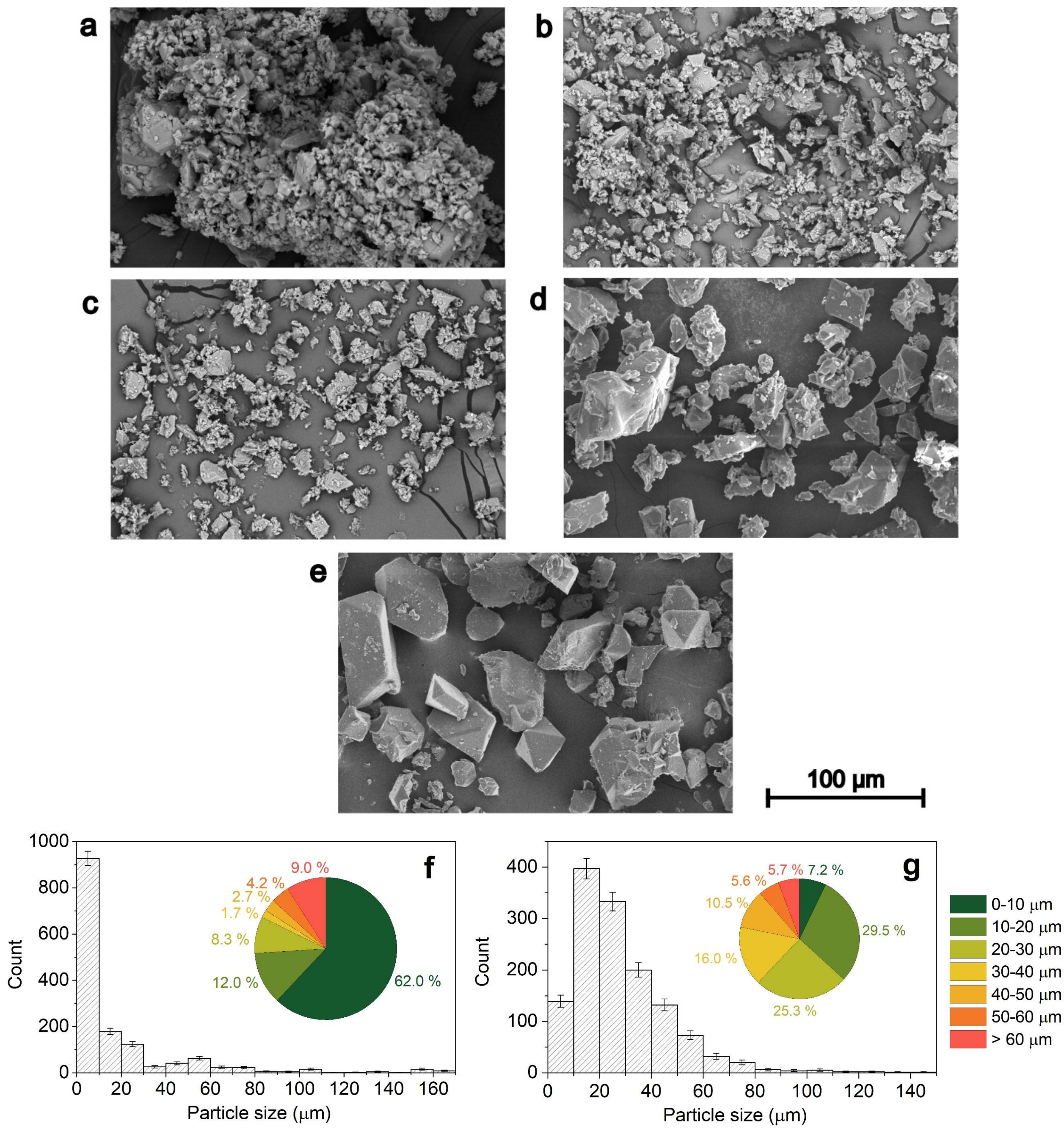

Supplementary Fig. 13. Analysis of particle sizes from SEM images. **a-e** SEM micrographs of ZIF-4(Zn) showing a typical representation of the particle population of different synthesised batches at identical magnification in **a** Batch A, **b** Batch B, **c** Batch C, **d** Batch D and **e** Batch E. **f,g** Particle size distribution of ZIF-4(Zn) **f** Batch A and **g** Batch E evaluated from SEM micrographs using projected area diameters from 12 micrographs of each sample using ImageJ. Error bars represent Poisson counting uncertainties as √N. Inset pie charts show the percentage distribution of particle sizes. N.B. This is a coarse semiquantitative estimation of the overall particle size distribution based on manual evaluation of particle size.

Supplementary Note 3

All micrographs showed agglomeration of particles which made accurate quantitative particle size analysis challenging, and required an arduous manual analysis due to lack of separation between particles. However, a coarse semiquantitative comparison can be made between different batches at similar magnification – Supplementary Fig. 13f,g shows a comparison between particle size distributions of Batches A and E based on 12 SEM micrographs of each batch, using fixed bin widths of 10 μm. The chosen bin size exceeds the estimated measurement uncertainty to minimize binning artefacts, and Poisson counting uncertainties are represented as $\sqrt{N}$ error bars. Owing to excessive agglomeration, undefined particle shapes and polydispersity, the following limitations in the manual particle size analysis are worth noting:

1. The number of particles that span <10 μm in Batch A is severely underrepresented. The particles taken into account are those that appear to be well-formed in images of up to × 10,000 magnification. Small specks of particles with undefined shapes visibly agglomerating to larger particles have not been accounted for, out of uncertainty as to whether they are ZIF-4(Zn) particles or unreacted imidazole crystals. Conversely, the count for larger particles is comparatively more accurate. Thereby, the percentage of particles spanning <10 μm is likely to be much higher than 62% in Batch A.

2. It is impossible to account for particles within agglomerates. Only particles visible on the surface have been accounted for. This also increases the potential particle count for smaller particles over bigger particles.

Regardless, this analysis was performed to obtain an overall idea of the rough particle size distributions of Batch A and E. While quantitatively the error is sizeable, qualitatively it is sufficient to suggest that the predominant particle size range in Batch A is <10 μm.

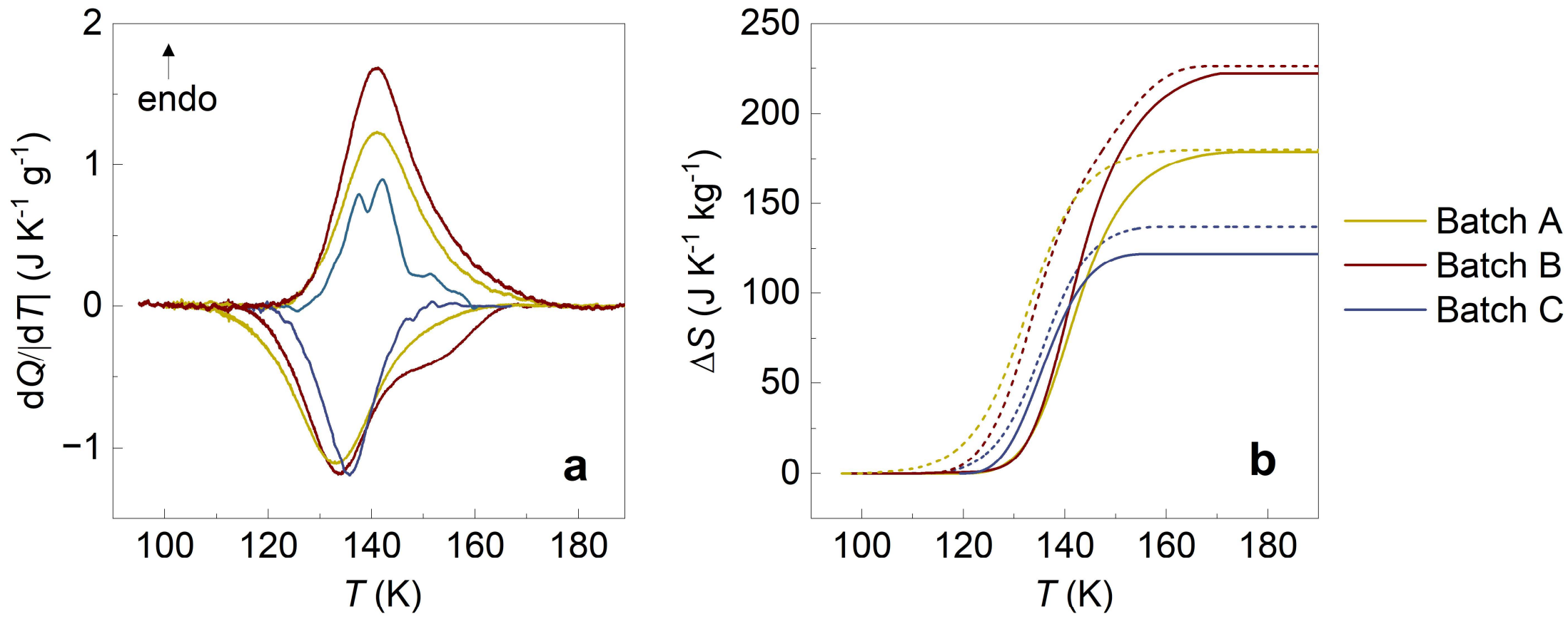

Supplementary Fig. 14. First-order crystallographic phase transition in desolvated ZIF-4 from Batches A, B and C. **a** Background-subtracted calorimetric peaks and **b** transition-related entropy changes with respect to the LT phase on heating (solid lines) and cooling (dotted lines) from Batches A, B and C before performing calorimetry under applied hydrostatic pressure using nitrogen gas.

Supplementary Note 4

The main text reports results from Batch A as this was the batch with the highest saved yield, which therefore permitted LT-XRD measurements and multiple calorimetric cycles over time to be performed on the same sample specimen. Batches B and C reproduced the caloric effect with diminished values of ΔS_{it} after pressurisation under nitrogen. However, due to lack of remaining sample after SEM and HP-DSC measurements, LT-XRD measurements were unable to be performed on these batches after high-pressure calorimetry.

Supplementary Table 1. Elapsed time (in months) of conducted trials of repeated isobaric calorimetric measurements in ZIF-4(Zn) since the first measurement (Trial 1). Values of $|Q_0|$ and $|\Delta S_0|$ have been averaged over all values obtained for the transition at different values of applied p from integral areas of calorimetric peaks, and have been normalised by total sample mass.

**Trial No.**	1	2	3	4	5
**Time (months)**	0	7	7.2	17	20
**$\|Q_0\|$ (kJ kg^{-1})**	2.8 ± 0.4	2.9 ± 0.2	2.5 ± 0.2	1.3 ± 0.2	0.98 ± 0.07
**$\|\Delta S_0\|$ (J K^{-1} kg^{-1})**	11.4 ± 1.8	12.0 ± 1.0	9.6 ± 1.0	3.9 ± 0.7	3.5 ± 0.3
**dT_0/dp (K kbar^{-1})**	75	76	73	69	67

Supplementary Table 2. Solvothermal synthesis conditions of the 5 batches of desolvated ZIF-4(Zn) samples. All oven synthesis were performed at 373 K and powders vacuum-filtered after cooling to RT. Filtered powder was then desolvated by stirring in DCM and finally heating in a vacuum oven.

Batch	Time in oven for synthesis	Cooling method	Time stirred in DCM	Temperature/Time in vacuum oven
A	72 hr	Immediately taken out of oven to cool at RT	28 hr	453 K/16 hr
B	96 hr	Immediately taken out of oven to cool at RT	20 hr	433 K/26 hr
C	84 hr	Slow natural cooling in oven	30 hr	410 K/16 hr
D	168 hr	Slow natural cooling in oven	23 hr	453 K/17 hr
E	72 hr	Slow natural cooling in oven	8 hr	423 K/16 hr

References

[1] Dilshad, M., Lampronti, G. I., Bennett, T. D. & Moya, X. Research Data supporting "Giant tunable barocaloric effects using guest-molecule adsorption in a porous metal-organic framework". Apollo - University of Cambridge Repository. https://doi.org/10.17863/CAM.121982.2 (2026).

[2] Wharmby, T. M. *et al*. Extreme flexibility in a zeolitic imidazolate framework: porous to dense phase transition in desolvated ZIF-4. *Angew. Chem., Int. Ed.* **54**, 6447–6451 (2015).
