# Supplementary material for: Giant tunable barocaloric effects using guest-molecule adsorption in a porous metal-organic framework
Source: Nat Commun. 2026 Jun 26;17:8077. doi: 10.1038/s41467-026-74616-6 (PMC13454139; doi:10.1038/s41467-026-74616-6)
Supplement: Supplementary file 2 [file 41467_2026_74616_MOESM2_ESM.pdf]

nature portfolio

Peer Review File

Giant tunable barocaloric effects using guest-molecule adsorption in a porous metal-organic framework

Corresponding Author: Dr Melony Dilshad

This file contains all reviewer reports in order by version, followed by all author rebuttals in order by version.

Version 0:

Reviewer comments:

Reviewer #1

(Remarks to the Author)
The manuscript from Dilshad et al. reports the thermodynamic behaviour of the zeolitic-imidazolate framework [ZIF-4(Zn)] as a candidate barocaloric material. The authors claim that this material exhibits giant reversible barocaloric effects engendered by a first-order phase transition. Using differential scanning calorimetry (DSC) under pressures ranging from ambient to 1 kbar, the authors report a phase transition temperature increase of 50 K, a transition entropy decrease of 180 J K−1 kg−1 to 16 J K−1 kg−1, and a pressure sensitivity of 76 K kbar−1 for the 16 J K−1 kg−1 phase transition. Owing to the increase in transition temperature, the authors claim ZIF-4(Zn) demonstrates 'tunable' barocaloric performance due to adsorption of the nitrogen gas used as the pressure transmitting fluid. While the authors present changing thermodynamic behaviour of ZIF-4(Zn) as a function of pressurization with nitrogen gas, the impacts of nitrogen exposure on the framework structure and consequently, phase-change properties, appear irreversible and uncontrollable. As a result, the opening statements and central claims in the abstract and manuscript are not fully supported, and at times seem to be contradicted, by the experimental data. Additional strategies to rationalize the data do not properly represent the decrease in thermodynamic performance and inability to cycle this material. From the experiments presented, the material appears incapable of surviving more than 5 total cycles, rendering any thermodynamic analysis of the phase transition performance misleading.

Major comments:
1. The material presented by the authors, [ZIF-4(Zn)], undergoes two seemingly irreversible phase transitions from the desolvated form: first the desolvated phase transforms to a 'nitrogenated' [ZIF-4(Zn)] phase upon exposure to nitrogen; this 'nitrogenated' [ZIF-4(Zn)] phase then seems to transition to the thermodynamically stable ZIF-zni phase upon repeated thermal or pressure cycling, as the authors state in lines 112–114. This ZIF-zni phase does not undergo any transition and creates 'inactive mass'. Given that the ZIF-zni phase is the most thermodynamically stable and does not undergo any reversible phase transitions, the suggested use of [ZIF-4(Zn)] in a barocaloric device or as a coolant generally is not well-supported by the data. The results here show a preponderance of evidence that this material would be rendered unusable very quickly after only a few cycles (see Figure 6c,d).
2. Central to the author's claim of 'tunable barocaloric performance' is the ability to control the phase transition temperature of ZIF-4(Zn). Although the authors demonstrate the ability to change the transition temperature upon application of pressure with nitrogen gas (Figure 4), this change appears stochastic and uncontrollable. For example, in Figure 6 they show transition temperature variations of nearly 20 K when the same pressure is applied (>400 bar, Trial 4 & 5 vs Trial 2 & 3). In Figures 10, S14, and S15 the authors show a large variability in all thermodynamic behaviour at increased pressures across five different samples of ZIF-4(Zn). These results do not illustrate control or predictability, with only a qualitative relationship to particle size offered as explanation without a full characterization of the five samples. This unpredictability of the adsorption is characterized as a 'dynamic' process, but no adsorption or structural data is provided to corroborate variations in nitrogen adsorbed across samples. Could the authors demonstrate a way to target specific transition temperatures with a known amount of nitrogen exposure? Further, in the context of a barocaloric cooling device, could the authors show how one could tune the transition temperature with the same stimulus used to drive the transition?
3. In its current form, the manuscript provides little data, mechanistic understanding, or evidence explaining how nitrogen uptake impacts ambient pressure transition temperature performance, key to the author's claim of nitrogen tunable ZIF-4(Zn). Figure 3 offers a qualitative study relating nitrogen exposure to transition temperature and entropy but the changes in entropy are an indirect measure of the nitrogen uptake. How did the authors reach these labels of partially nitrogenated and nitrogenated? To understand how nitrogen uptake impacts transition behaviour, the data presented here likely need more

than one point between the desolvated and fully nitrogenated form.
4. In several places, the authors offered a normalization of their measured entropy to 'active' mass (in lines 67–68 for the desolvated structure and again in Figure 4 for the post-nitrogen exposed sample). The authors justify this practice given that irreversible phase transitions of this material result in some fraction of 'inactive mass' during phase transition cycling. However, the 'inactive mass' does not appear capable of regeneration, so normalizing the transition entropy based on 'active mass' seems misleading. While this analysis may suggest that the entropy change is higher than measured, this practice makes it challenging for new readers to understand the actual performance of a given material.
5. Figure 1 appears to be the only DSC measurement provided for the desolvated ZIF. However, the authors then later provide isothermal entropy curves and adiabatic temperature changes for the desolvated ZIF in Figure 7. From the authors' discussion, this desolvated framework seems like it cannot be exposed to nitrogen without undergoing a transition to the much lower entropy-change material. In this case, how could the authors have constructed isothermal entropy curves that are determined from high-pressure DSC measurements? The underlying data for the red plots of isothermal entropy changes and adiabatic temperature changes in Figure 7 are essential to understand and support one of the central claims stated in the abstract: "For desolvated frameworks of ZIF-4(Zn), we report larger barocaloric effects of $|\Delta S_{it}|$ ~ 210 J K^{-1} kg^{-1} and $|\Delta T_{ad}|$ ~ 60 K near T_0 ~ 137 K driven using the same pressure change." If these plots are estimated based on other data sets (for example, the high-pressure DSC of the 'nitrogenated' ZIF), this should be clearly stated, and the relevance of such estimates would seem highly uncertain.
6. Beyond the technical issues described above, the manuscript formatting makes the text very challenging to follow. There are numerous times throughout the manuscript where the authors introduce a concept that they say they will explain later in the manuscript. While this occasionally is reasonable, the text here uses this tool much too often. The strategy of referencing future sections means that readers must jump around the manuscript frequently to understand non-standard concepts. The readability of the manuscript would need to be improved to be accessible to the broad readership of Nature Communications.

Minor Comments:
1. In multiple cases, the authors use the descriptor 'nitrogenated' to describe the framework with nitrogen gas present. This verbiage, nitrogenated, is often used to describe a covalent chemical transformation, whereas the effect here is adsorption and desorption of nitrogen gas. This wording should be amended in all cases to something related to nitrogen gas uptake.
2. Despite the comments for SEM particle sizing errors, could the author's include error bars within Figure 9?
3. Could the authors expand on their comments in lines 306-308 'Calorimetric data for Batches B and C under applied pressure showed multiple peaks shifting with pressure, indicating the dynamic impact of nitrogenation on the transition.' How does shifting of multiple peaks show the dynamic impact of the application of pressure?
4. Why does the DSC peak shape change at higher temperatures in Figure 8?
5. Could the authors provide a timescale for the pressurization and depressurization in Figure 8?
6. What are the thermodynamic parameters of Batch D and E? Particle sizes for E are used for comparison, but only the DSC traces are shown in Figures S14 and S15, with no analysis in the main text.
7. Line 42 – "low applied pressures below 1 kbar". The word low should be removed. Vapor-compression systems run in the 'low' pressure range 2-20 bar which is well below the ceiling of 1 kbar.

Reviewer #2

(Remarks to the Author)
Dr Dilshad and colleagues report giant but yet tunable barocaloric effects in the metal-organic framework (MOF) ZIF-4(Zn). Porosity is a key feature of MOF families. The authors noticed the effect of guest molecules in ZIF-4 and uncovered their function in response to pressure and the barocaloric effect. In detail, ZIF-4 presents a giant entropy change of 38 J K^{-1} kg^{-1} when N_2 is occupied in the pore. When the N_2 is removed from the pore, a colossal entropy change of 210 J K^{-1} kg^{-1} is obtained instead. This study highlights the potential of MOFs as promising barocaloric refrigerants and also a new cooling technology by tuning the amount of guest molecules.
In my opinion, this paper shows great novelty in presenting a colossal barocaloric effect in ZIF-4, while opening a way to manipulate the barocaloric effect in MOFs through their porous structural property. MOFs are a large family of materials with designable porosity. I believe that this work could potentially establish a playground for MOFs as environmentally benign solid-state refrigerants that can be tuned using guest molecules. Therefore, I would highly recommend this work for publishing in Nature Communications. Also, there are some minor corrections for your consideration:
1. It would be nice to present a simple crystal structure of ZIF-4(Zn) in Fig. 1 or in the figures in the Supplementary Materials for readers to better understand ZIF-4(Zn) and its porosity.
2. It is interesting to know the structural stability or durability of ZIF-4 with and without N_2 gas under the pressure used in this work.
3. In line 49, the authors prepare activated ZIF by removing solvent from pores. How about the N_2 and O_2 from air—are they also removed?
4. In line 261, the authors integrated the isothermal calorimetric peaks to get the isothermal heat. If I understand it correctly, one must have a reference value under ambient pressure and then normalize all the peak values from ambient pressure to high pressure. But this is not the case for a custom high-pressure DSC set-up. So it would be good to point out the set-up used here.
5. In the section "Evaluation of barocaloric effects" in Methods, the specific heats of LT and HT are essential for constructing the entropy change curves. Is there any difference in specific heat between nitrogenated and desolvated ZIF?
Suggestion for layout:
1. Please check the reference style in the main text. The authors may need to change "ref [xx]" or "[refs. xx]" directly to "[]".
2. There are too many figures in the main text; please make sure this is in line with the construction requirements of Nature Communications. The authors may consider merging some figures.

3. It is very interesting to see the influence of particle size on the barocaloric response. I would suggest merging Figures 8 and 10 to make the storyline clean, and putting Figure 9 into the Supplementary Materials.

Version 1:

Reviewer comments:

Reviewer #1

(Remarks to the Author)
The authors provide substantial improvements to the manuscript overall. In particular, the clarity in both text and figures are quite useful. The transparency about the different batches in the final section also provides a nice contextualization for the batch to batch variation that the authors (and likely others in the field) observe in thermal performance. I thank the authors for their careful consideration and revisions. The work is suitable for publication in Nature Communications.

Reviewer #2

(Remarks to the Author)
Dear Editor,

The authors have satisfactorily addressed the comments and questions. I have no further concerns and believe the current version is suitable for publication.Nice work!

Best wishes

Open Access This Peer Review File is licensed under a Creative Commons Attribution 4.0 International License, which permits use, sharing, adaptation, distribution and reproduction in any medium or format, as long as you give appropriate credit to the original author(s) and the source, provide a link to the Creative Commons license, and indicate if changes were made.
In cases where reviewers are anonymous, credit should be given to 'Anonymous Referee' and the source.
The images or other third party material in this Peer Review File are included in the article’s Creative Commons license, unless indicated otherwise in a credit line to the material. If material is not included in the article’s Creative Commons license and your intended use is not permitted by statutory regulation or exceeds the permitted use, you will need to obtain permission directly from the copyright holder.
To view a copy of this license, visit https://creativecommons.org/licenses/by/4.0/

Peer Review File

Giant tunable barocaloric effects using guest-molecule adsorption in a porous metal-organic framework

Corresponding Author: Dr Melony Dilshad

This file contains all reviewer reports in order by version, followed by all author rebuttals in order by version.

Version 0:

Reviewer comments:

Reviewer #1

(Remarks to the Author)
The manuscript from Dilshad et al. reports the thermodynamic behaviour of the zeolitic-imidazolate framework [ZIF-4(Zn)] as a candidate barocaloric material. The authors claim that this material exhibits giant reversible barocaloric effects engendered by a first-order phase transition. Using differential scanning calorimetry (DSC) under pressures ranging from ambient to 1 kbar, the authors report a phase transition temperature increase of 50 K, a transition entropy decrease of 180 J K−1 kg−1 to 16 J K−1 kg−1, and a pressure sensitivity of 76 K kbar−1 for the 16 J K−1 kg−1 phase transition. Owing to the increase in transition temperature, the authors claim ZIF-4(Zn) demonstrates 'tunable' barocaloric performance due to adsorption of the nitrogen gas used as the pressure transmitting fluid. While the authors present changing thermodynamic behaviour of ZIF-4(Zn) as a function of pressurization with nitrogen gas, the impacts of nitrogen exposure on the framework structure and consequently, phase-change properties, appear irreversible and uncontrollable. As a result, the opening statements and central claims in the abstract and manuscript are not fully supported, and at times seem to be contradicted, by the experimental data. Additional strategies to rationalize the data do not properly represent the decrease in thermodynamic performance and inability to cycle this material. From the experiments presented, the material appears incapable of surviving more than 5 total cycles, rendering any thermodynamic analysis of the phase transition performance misleading.

Major comments:
1. The material presented by the authors, [ZIF-4(Zn)], undergoes two seemingly irreversible phase transitions from the desolvated form: first the desolvated phase transforms to a 'nitrogenated' [ZIF-4(Zn)] phase upon exposure to nitrogen; this 'nitrogenated' [ZIF-4(Zn)] phase then seems to transition to the thermodynamically stable ZIF-zni phase upon repeated thermal or pressure cycling, as the authors state in lines 112–114. This ZIF-zni phase does not undergo any transition and creates 'inactive mass'. Given that the ZIF-zni phase is the most thermodynamically stable and does not undergo any reversible phase transitions, the suggested use of [ZIF-4(Zn)] in a barocaloric device or as a coolant generally is not well-supported by the data. The results here show a preponderance of evidence that this material would be rendered unusable very quickly after only a few cycles (see Figure 6c,d).
2. Central to the author's claim of 'tunable barocaloric performance' is the ability to control the phase transition temperature of ZIF-4(Zn). Although the authors demonstrate the ability to change the transition temperature upon application of pressure with nitrogen gas (Figure 4), this change appears stochastic and uncontrollable. For example, in Figure 6 they show transition temperature variations of nearly 20 K when the same pressure is applied (>400 bar, Trial 4 & 5 vs Trial 2 & 3). In Figures 10, S14, and S15 the authors show a large variability in all thermodynamic behaviour at increased pressures across five different samples of ZIF-4(Zn). These results do not illustrate control or predictability, with only a qualitative relationship to particle size offered as explanation without a full characterization of the five samples. This unpredictability of the adsorption is characterized as a 'dynamic' process, but no adsorption or structural data is provided to corroborate variations in nitrogen adsorbed across samples. Could the authors demonstrate a way to target specific transition temperatures with a known amount of nitrogen exposure? Further, in the context of a barocaloric cooling device, could the authors show how one could tune the transition temperature with the same stimulus used to drive the transition?
3. In its current form, the manuscript provides little data, mechanistic understanding, or evidence explaining how nitrogen uptake impacts ambient pressure transition temperature performance, key to the author's claim of nitrogen tunable ZIF-4(Zn). Figure 3 offers a qualitative study relating nitrogen exposure to transition temperature and entropy but the changes in entropy are an indirect measure of the nitrogen uptake. How did the authors reach these labels of partially nitrogenated and nitrogenated? To understand how nitrogen uptake impacts transition behaviour, the data presented here likely need more

than one point between the desolvated and fully nitrogenated form.
4. In several places, the authors offered a normalization of their measured entropy to ‘active’ mass (in lines 67–68 for the desolvated structure and again in Figure 4 for the post-nitrogen exposed sample). The authors justify this practice given that irreversible phase transitions of this material result in some fraction of ‘inactive mass’ during phase transition cycling. However, the ‘inactive mass’ does not appear capable of regeneration, so normalizing the transition entropy based on ‘active mass’ seems misleading. While this analysis may suggest that the entropy change is higher than measured, this practice makes it challenging for new readers to understand the actual performance of a given material.
5. Figure 1 appears to be the only DSC measurement provided for the desolvated ZIF. However, the authors then later provide isothermal entropy curves and adiabatic temperature changes for the desolvated ZIF in Figure 7. From the authors’ discussion, this desolvated framework seems like it cannot be exposed to nitrogen without undergoing a transition to the much lower entropy-change material. In this case, how could the authors have constructed isothermal entropy curves that are determined from high-pressure DSC measurements? The underlying data for the red plots of isothermal entropy changes and adiabatic temperature changes in Figure 7 are essential to understand and support one of the central claims stated in the abstract: “For desolvated frameworks of ZIF-4(Zn), we report larger barocaloric effects of $|\Delta S_{it}|$ ~ 210 J K−1 kg−1 and $|\Delta T_{ad}|$ ~ 60 K near T0 ~ 137 K driven using the same pressure change.” If these plots are estimated based on other data sets (for example, the high-pressure DSC of the ‘nitrogenated’ ZIF), this should be clearly stated, and the relevance of such estimates would seem highly uncertain.
6. Beyond the technical issues described above, the manuscript formatting makes the text very challenging to follow. There are numerous times throughout the manuscript where the authors introduce a concept that they say they will explain later in the manuscript. While this occasionally is reasonable, the text here uses this tool much too often. The strategy of referencing future sections means that readers must jump around the manuscript frequently to understand non-standard concepts. The readability of the manuscript would need to be improved to be accessible to the broad readership of Nature Communications.

Minor Comments:
1. In multiple cases, the authors use the descriptor ‘nitrogenated’ to describe the framework with nitrogen gas present. This verbiage, nitrogenated, is often used to describe a covalent chemical transformation, whereas the effect here is adsorption and desorption of nitrogen gas. This wording should be amended in all cases to something related to nitrogen gas uptake.
2. Despite the comments for SEM particle sizing errors, could the author’s include error bars within Figure 9?
3. Could the authors expand on their comments in lines 306-308 ‘Calorimetric data for Batches B and C under applied pressure showed multiple peaks shifting with pressure, indicating the dynamic impact of nitrogenation on the transition.’ How does shifting of multiple peaks show the dynamic impact of the application of pressure?
4. Why does the DSC peak shape change at higher temperatures in Figure 8?
5. Could the authors provide a timescale for the pressurization and depressurization in Figure 8?
6. What are the thermodynamic parameters of Batch D and E? Particle sizes for E are used for comparison, but only the DSC traces are shown in Figures S14 and S15, with no analysis in the main text.
7. Line 42 – “low applied pressures below 1 kbar”. The word low should be removed. Vapor-compression systems run in the ‘low’ pressure range 2-20 bar which is well below the ceiling of 1 kbar.

Reviewer #2

(Remarks to the Author)
Dr Dilshad and colleagues report giant but yet tunable barocaloric effects in the metal-organic framework (MOF) ZIF-4(Zn). Porosity is a key feature of MOF families. The authors noticed the effect of guest molecules in ZIF-4 and uncovered their function in response to pressure and the barocaloric effect. In detail, ZIF-4 presents a giant entropy change of 38 $J K^{-1} kg^{-1}$ when N_2 is occupied in the pore. When the N_2 is removed from the pore, a colossal entropy change of 210 $J K^{-1} kg^{-1}$ is obtained instead. This study highlights the potential of MOFs as promising barocaloric refrigerants and also a new cooling technology by tuning the amount of guest molecules.
In my opinion, this paper shows great novelty in presenting a colossal barocaloric effect in ZIF-4, while opening a way to manipulate the barocaloric effect in MOFs through their porous structural property. MOFs are a large family of materials with designable porosity. I believe that this work could potentially establish a playground for MOFs as environmentally benign solid-state refrigerants that can be tuned using guest molecules. Therefore, I would highly recommend this work for publishing in Nature Communications. Also, there are some minor corrections for your consideration:
1. It would be nice to present a simple crystal structure of ZIF-4(Zn) in Fig. 1 or in the figures in the Supplementary Materials for readers to better understand ZIF-4(Zn) and its porosity.
2. It is interesting to know the structural stability or durability of ZIF-4 with and without N_2 gas under the pressure used in this work.
3. In line 49, the authors prepare activated ZIF by removing solvent from pores. How about the N_2 and O_2 from air—are they also removed?
4. In line 261, the authors integrated the isothermal calorimetric peaks to get the isothermal heat. If I understand it correctly, one must have a reference value under ambient pressure and then normalize all the peak values from ambient pressure to high pressure. But this is not the case for a custom high-pressure DSC set-up. So it would be good to point out the set-up used here.
5. In the section “Evaluation of barocaloric effects” in Methods, the specific heats of LT and HT are essential for constructing the entropy change curves. Is there any difference in specific heat between nitrogenated and desolvated ZIF?
Suggestion for layout:
1. Please check the reference style in the main text. The authors may need to change “ref [xx]” or “[refs. xx]” directly to “[]”.
2. There are too many figures in the main text; please make sure this is in line with the construction requirements of Nature Communications. The authors may consider merging some figures.

3. It is very interesting to see the influence of particle size on the barocaloric response. I would suggest merging Figures 8 and 10 to make the storyline clean, and putting Figure 9 into the Supplementary Materials.

Version 1:

Reviewer comments:

Reviewer #1

(Remarks to the Author)
The authors provide substantial improvements to the manuscript overall. In particular, the clarity in both text and figures are quite useful. The transparency about the different batches in the final section also provides a nice contextualization for the batch to batch variation that the authors (and likely others in the field) observe in thermal performance. I thank the authors for their careful consideration and revisions. The work is suitable for publication in Nature Communications.

Reviewer #2

(Remarks to the Author)
Dear Editor,

The authors have satisfactorily addressed the comments and questions. I have no further concerns and believe the current version is suitable for publication.Nice work!

Best wishes

Open Access This Peer Review File is licensed under a Creative Commons Attribution 4.0 International License, which permits use, sharing, adaptation, distribution and reproduction in any medium or format, as long as you give appropriate credit to the original author(s) and the source, provide a link to the Creative Commons license, and indicate if changes were made.
In cases where reviewers are anonymous, credit should be given to 'Anonymous Referee' and the source.
The images or other third party material in this Peer Review File are included in the article’s Creative Commons license, unless indicated otherwise in a credit line to the material. If material is not included in the article’s Creative Commons license and your intended use is not permitted by statutory regulation or exceeds the permitted use, you will need to obtain permission directly from the copyright holder.
To view a copy of this license, visit https://creativecommons.org/licenses/by/4.0/

RESPONSE TO REVIEWERS' COMMENTS

Referee #1:

We thank the referee for the detailed feedback, which has inspired us to make major improvements. Purely for brevity, our response does not thank the referee for each of the individual technical comments, but the referee should rest assured that we really appreciate the input.

All changes (except figure changes) are shown using yellow highlights. These changes include changes due to feedback from all referees, changes where we found ways to improve the presentation, and changes that we made to comply with Editorial advice to e.g. include a Data Availability section after the Methods section but before the References.

Figure changes are as follows:

- In Fig. 1(e), using the feedback of Referee 1, we have added calorimetry curves at 0 bar for samples after nitrogen exposure of 12 hours and 22 hours.
- In Fig. 1(f), using the feedback of Referee 1, we now show the changes in the thermodynamic parameters of the first-order phase transition as a function of nitrogen exposure time.
- In Fig. 4, using the feedback of Referee 1, we have removed the linear guide to the eye.
- In Fig. 6(f), using the feedback of Referee 1, we have added a graph showing comparison between transition pressures required to drive our ZIF-4 samples with those reported in the literature.
- Using the feedback of Referee 2, we have merged Figs 1-3, and Figs 8 and 10, in the original manuscript, and moved Fig. 9 to Supplementary Information. Also, we now show Figs S15(a) and (b) in the main manuscript as Fig. 6(c,d).
- All figure colour schemes have been improved for better readability.

The revised abstract now has 200 words, and the revised main text now has 4823 words, which is below the specified word limit of 5000.

The source data for calorimetry and scanning electron microscopy are included in a zipped folder labelled Source Data, along with x-ray diffraction data in the University of Cambridge repository Apollo.

The manuscript from Dilshad et al. reports the thermodynamic behaviour of the zeolitic-imidazolate framework [ZIF-4(Zn)] as a candidate barocaloric material. The authors claim that this material exhibits giant reversible barocaloric effects engendered by a first-order phase transition.

The barocaloric effects that we present were determined experimentally using variable-pressure calorimetry in nitrogen-loaded ZIF-4(Zn) samples. The corresponding barocaloric entropy changes are giant ($|\Delta S_{it}| \sim 38$ J K^{-1} kg^{-1} when driven by $|\Delta p| = 1$ kbar) and reversible

(i.e. same values of $|\Delta S_{it}|$ when applying, $0 \rightarrow p$, and removing, $p \rightarrow 0$, pressure) over a wide range of temperatures.

Using differential scanning calorimetry (DSC) under pressures ranging from ambient to 1 kbar, the authors report a phase transition temperature increase of 50 K, a transition entropy decrease of 180 J K^{-1} kg^{-1} to 16 J K^{-1} kg^{-1}, and a pressure sensitivity of 76 K kbar^{-1} for the 16 J K^{-1} kg^{-1} phase transition.

The changes in entropy of 180 J K^{-1} kg^{-1} and 16 J K^{-1} kg^{-1} shown in the original Figure 3 were determined experimentally using calorimetry at atmospheric pressure only, and therefore they are not barocaloric entropy changes. These values characterise the thermodynamic properties of thermally driven phase transitions at zero applied pressure in desolvated and nitrogen-loaded ZIF4(Zn) samples, respectively.

Owing to the increase in transition temperature, the authors claim ZIF-4(Zn) demonstrates 'tunable' barocaloric performance due to adsorption of the nitrogen gas used as the pressure transmitting fluid.

The thermodynamic properties of the thermally driven phase transition in ZIF-4(Zn) at atmospheric pressure can be indeed tuned by adjusting the exposure time to nitrogen.

We are sorry to see that this information was not clearly presented in the manuscript, and for this we apologise. To rectify the matter, we have now:

- added additional calorimetry data at 0 bar for samples after nitrogen exposure of 12 hours and 22 hours [new Fig. 1e],
- shown the changes in the thermodynamic parameters of the first-order phase transition as a function of nitrogen exposure time [new Fig. 1f].

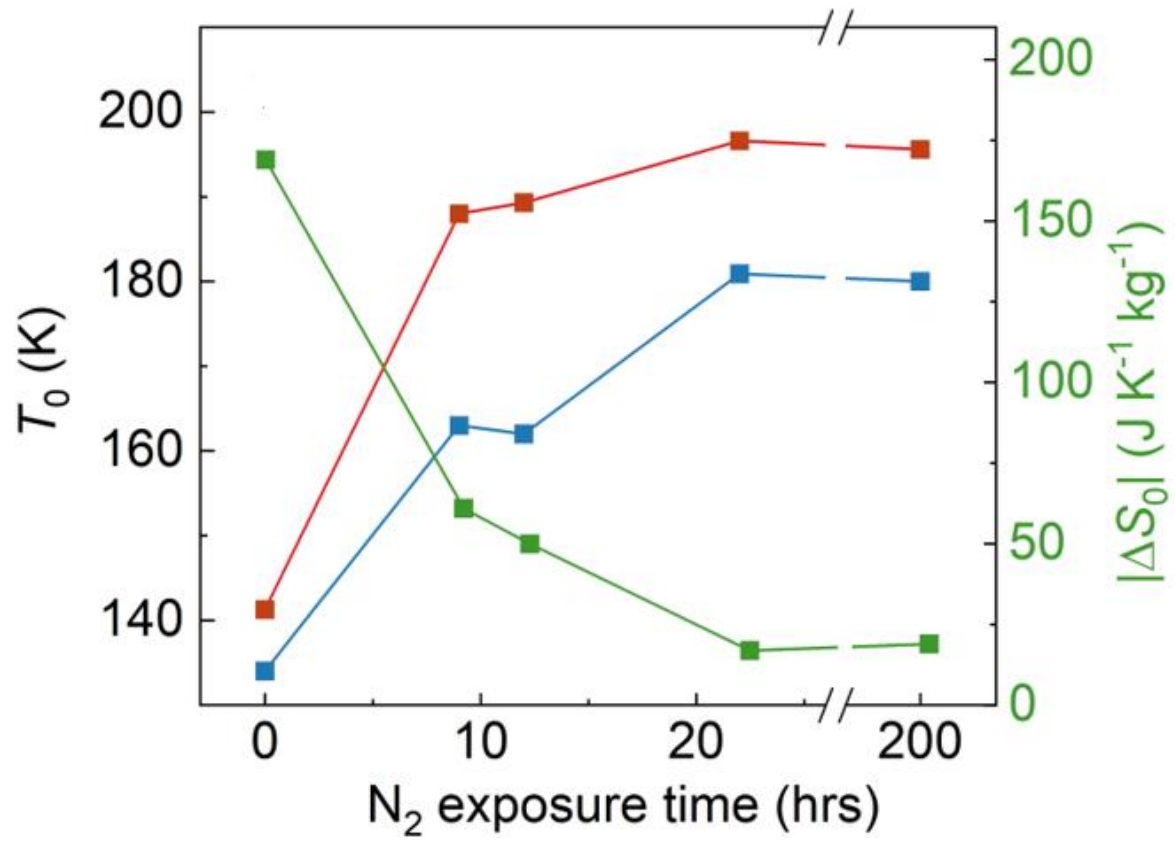

New Figure 1(f). Transition temperature and thermally driven entropy change for the first-order transition in ZIF-4(Zn) as a function of exposure time to N_2.

We have also added to the manuscript the text below to differentiate the use of nitrogen as a (i) tuning guest molecule, and (ii) pressure transmitting medium:

"This barocaloric effect could potentially be tuned to higher operating temperatures by using small amounts of adsorbed nitrogen as a tuning guest molecule in ZIF-4(Zn), which can then be driven in cooling systems using a large-molecule PTF, such as the hydraulic oils used in barocaloric prototypes."

While the authors present changing thermodynamic behaviour of ZIF-4(Zn) as a function of pressurization with nitrogen gas, the impacts of nitrogen exposure on the framework structure and consequently, phase-change properties, appear irreversible and uncontrollable. As a result, the opening statements and central claims in the abstract and manuscript are not fully supported, and at times seem to be contradicted, by the experimental data.

The tunability of transition temperature with nitrogen adsorption is irreversible but controllable [new Figure 1(f) above]. This is intended and desirable in order to produce for example a ZIF-4(Zn) barocaloric refrigerant with a certain transition temperature, or a cascaded composite based on ZIF-4(Zn) alone, which are both useful for applications.

We note that in prototypes, barocaloric materials are driven (for practical and safety reasons) using hydraulics with e.g. silicone oil as pressure transmitting fluid (www.barocal.com). The irreversibility of the transition to ZIF-*zni* here seems to have happened because we used nitrogen also as pressure transmitting fluid (due to the characteristics of our measurement setup), which is likely to have overloaded the framework pores after prolonged high-pressure exposure. The transition to ZIF-*zni* is not reported to occur when using non-penetrating, large-molecule pressure transmitting fluids such as silicone oil, fluorinert or mercury [Vervoorts *et al. Z. Anorg. Allg. Chem.* **645**, 970 – 974 (2019), Henke *et al. Chem. Sci*. **9**, 1654-1660 (2018)].

Additional strategies to rationalize the data do not properly represent the decrease in thermodynamic performance and inability to cycle this material. From the experiments presented, the material appears incapable of surviving more than 5 total cycles, rendering any thermodynamic analysis of the phase transition performance misleading.

We demonstrated giant reversible barocaloric effects in nitrogen-loaded ZIF-4(Zn) by performing quasi-direct and direct measurements in 3 separate samples, with >40 combined cycles in a single specimen.

Moreover, a recent study by Li *et al.* [Journal of Materials Science & Technology **264**, 82 (2026)] reports large barocaloric effects in unloaded ZIF-4(Zn) and shows reversibility over more than 100 heating and cooling cycles, before stopping testing.

Major comments:

1. The material presented by the authors, [ZIF-4(Zn)], undergoes two seemingly irreversible phase transitions from the desolvated form: first the desolvated phase transforms to a 'nitrogenated' [ZIF-4(Zn)] phase upon exposure to nitrogen; this 'nitrogenated' [ZIF-4(Zn)] phase then seems to transition to the thermodynamically stable ZIF-zni phase upon repeated thermal or pressure cycling, as the authors state in lines 112–114. This ZIF-zni phase does not undergo any transition and creates 'inactive mass'. Given that the ZIF-zni phase is the most thermodynamically stable and does not undergo any reversible phase transitions, the suggested use of [ZIF-4(Zn)] in a barocaloric device or as a coolant generally is not well-supported by the data. The results here show a preponderance of evidence that this material would be rendered unusable very quickly after only a few cycles (see Figure 6c,d).

The presence of active and inactive material (mass) is common in the field of calorics, and accounting for the presence of inactive mass is good practice, e.g. in magnetocalorics [Moya *et al.*, Nature Materials **12**, 52 (2013)] and in electrocalorics [Nair *et al.*, Nature **575**, 468 (2019)]. The presence of inactive mass does not stop caloric materials from being used in cooling systems. For example, the electrocaloric materials reported by Nair *et al.* [Nature **575**, 468 (2019)] have been successfully in several electrocaloric prototypes, e.g. Torelló *et al.*, Science **370**, 125 (2020), Wang *et al.*, Science **370**, 129 (2020) and Li *et al.*, Science **382**, 801 (2023).

The irreversible adsorption of nitrogen into ZIF-4(Zn) is desirable for tuning the transition temperature.

The irreversible transition to ZIF-*zni* seems to have happened because we used nitrogen also as the pressure-transmitting fluid (due to the characteristics of our measurement setup) which is likely to have overloaded the framework pores after prolonged high-pressure exposure. This can be prevented by using a non-penetrating, large-molecule pressure transmitting fluid, as it is the case in prototypes that are driven using safe hydraulic systems with silicone oil (www.barocal.com), or in the recent study by Li *et al.* [Journal of Materials Science & Technology **264**, 82 (2026)] using the same pressure transmitting fluid.

We have now added the relevant literature and clarified this in the revised manuscript:

When using non-penetrating PTFs such as mercury[43], fluorinert[43] or silicone oil[44], no transition to ZIF-*zni* has been reported under the temperature and pressure conditions used in our work. Moreover, this structural phase transition in ZIF-4(Zn) is fully reversible when driven using silicone oil AP 100, with its large transition $|\Delta V_0|/V \sim 20\%$ and original ZIF-4(Zn) HT orthorhombic structure retained[44] after having been exposed to much higher pressures of $p = 4$ kbar.

We note that Figure 6(c,d) in the original submission showed the evolution of the thermodynamic properties of the thermally driven phase transitions at each applied pressure (therefore no barocaloric heat or barocaloric entropy changes) in nitrogen-loaded samples over an extended 20-months period of time. Within error, the data show no significant variation in

the thermodynamic properties within 7 months after the initial nitrogen loading. The observed changes after 17 and 20 months may have been caused by the storing of samples at ambient conditions in vials that were not perfectly sealed. We note that Figure 6(a,b) in the original submission show that dT/dp, which is not an extensive quantity, is nominally the same after 20 months, indicating that the active ZIF-4(Zn) present retains its responsiveness to pressure and therefore barocaloric performance.

We now realised that the dotted guide-to-the-eye lines shown in the original Figure 6(c,d) were misleading, and for this we apologise. We have removed the guide-to-the-eye lines in the revised Figure 6(c,d) (now Fig. 4c,d) and we have added the explanation below to the manuscript:

"Calorimetry indicates that $|Q_0|$ and $|\Delta S_0|$ (normalised by total sample mass now for simplicity) associated with the first-order phase transition are nominally the same over trials 1-3 up to ~7 months. These values decrease by ~70 % after 17 months and we note that our samples were stored at ambient temperature and pressure in vials that were not perfectly sealed during that period. The large decrease in $|Q_0|$ and $|\Delta S_0|$, which are extensive quantities, after 17 months could be attributed to increased adsorption of atmospheric nitrogen, which has been shown to occur even at ambient conditions[46], and to further conversion of nitrogen-loaded ZIF-4 to ZIF-*zni* after repeated exposure to nitrogen at high pressures. Nevertheless, dT_0/dp, which is an intensive thermodynamic quantity, decreased rather less by ~10 % (from ~76 to 67 K kbar^{-1}) after 20 months. The small decrease in dT_0/dp could be an indication that the transition ΔV_0 also slightly decreases with increased nitrogen loading into ZIF-4 pores, which may hinder the rotation of its imidazole linkers[34]. Thus, while our sample specimen appears to show a reduction in $|\Delta S_0|$ normalised by total mass, this is consistent with an increase in inactive mass from ZIF-*zni*."

The new Fig. 4c,d after removing the linear guide to eye is shown below:

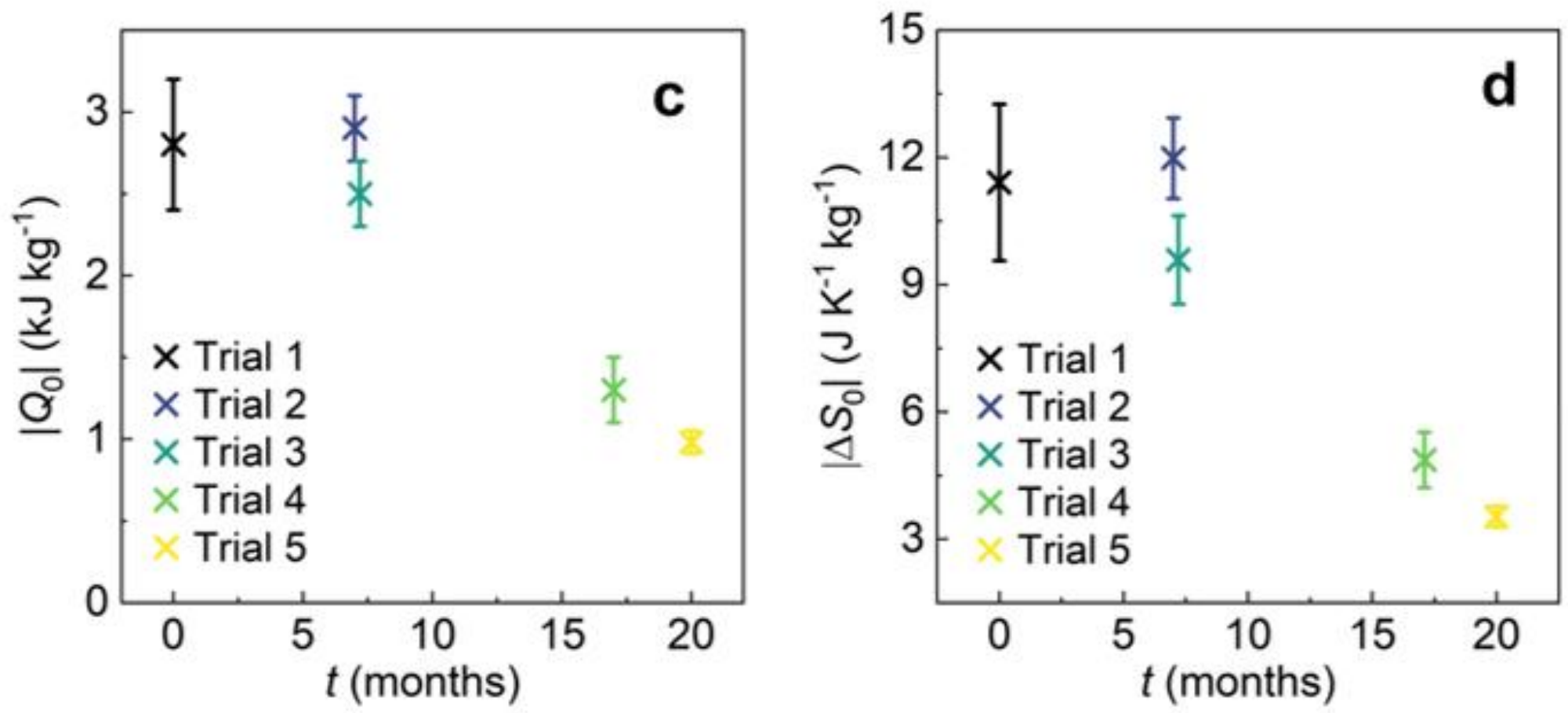

New Fig. 4c,d: Variation in **c** latent heat $|Q_0|$ and **d** transition entropy change $|\Delta S_0|$ of nitrogen-loaded ZIF 4(Zn) over the different trials.

2. Central to the author's claim of 'tunable barocaloric performance' is the ability to control the phase transition temperature of ZIF-4(Zn). Although the authors demonstrate the ability to change the transition temperature upon application of pressure with nitrogen gas (Figure 4), this change appears stochastic and uncontrollable. For example, in Figure 6 they show transition temperature variations of nearly 20 K when the same pressure is applied (>400 bar, Trial 4 & 5 vs Trial 2 & 3). In Figures 10, S14, and S15 the authors show a large variability in all thermodynamic behaviour at increased pressures across five different samples of ZIF-4(Zn). These results do not illustrate control or predictability, with only a qualitative relationship to particle size offered as explanation without a full characterization of the five samples. This unpredictability of the adsorption is characterized as a 'dynamic' process, but no adsorption or structural data is provided to corroborate variations in nitrogen adsorbed across samples. Could the authors demonstrate a way to target specific transition temperatures with a known amount of nitrogen exposure? Further, in the context of a barocaloric cooling device, could the authors show how one could tune the transition temperature with the same stimulus used to drive the transition?

Figure 4 shows peak transition temperatures on heating and cooling in multiple trials performed on *Batch A*. When transition widths are taken into account, most transitions at a given applied pressure tend to overlap, indicating an average shift and expected range in the transition temperature at that value of applied pressure. This is shown in the figure below, which is adapted from Figure 4 in the main manuscript. Colour bands have been added here to show transition widths from all cycles in the five Trials. These colour bands are not shown in the manuscript to maintain clarity for the individual points.

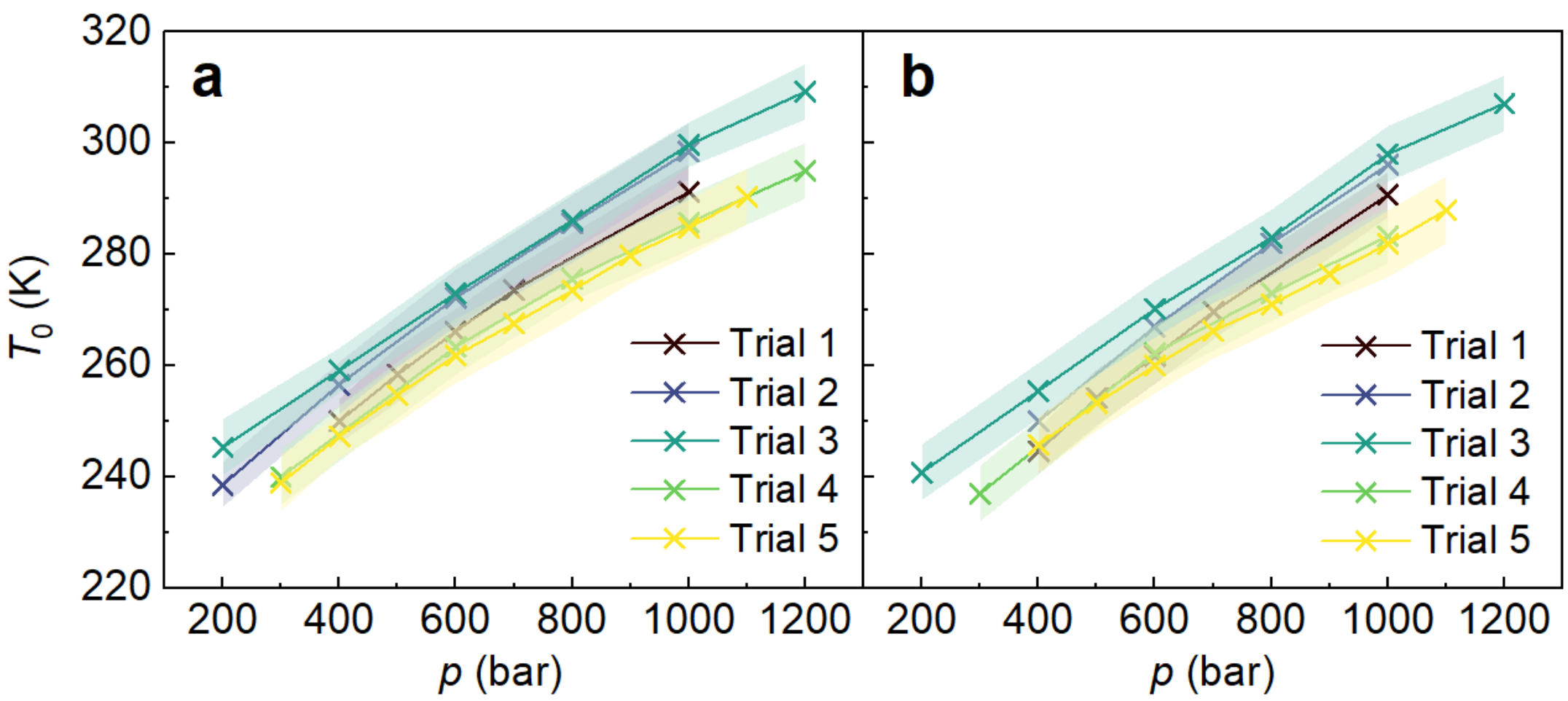

Adapted **Figure 4a,b**: Transition temperatures, T_0, of nitrogen-loaded ZIF-4(Zn) on **a** heating and **b** cooling under different values of applied p from five repeated isobaric calorimetry trials. Colour bands show transition widths. These bands are not shown in the our manuscript to maintain clarity for the points.

We note that the apparent 'stochasticity' is only between different experiments (Batches and Trials), and not within the experiment. For each given Batch and Trial, the shift in T_0 with pressure is not stochastic - $\mathrm{d}T_0/\mathrm{d}p$ is monotonic and controllable. This has been observed in other barocaloric materials, including the prototypical plastic crystal neopentyl glycol (Rendell-Bhatti *et al* 2024 *J. Phys. Energy* **6** 025020), to a lesser extent. We now clarify this in our revised manuscript:

"Also, it can be noted from Fig. 4a,b that the T_0 of nitrogen-loaded ZIF-4(Zn) at a given value of applied p varies up to ~20 K between different trials in an apparently stochastic manner, but the $\mathrm{d}T_0/\mathrm{d}p$ of each individual trial is monotonic. Such stochasticity is a thermodynamic feature common for first-order phase transitions, which is observed to less extent in other barocaloric materials, and is likely a consequence of different amounts of adsorbed nitrogen stabilising the LT phase to varying degrees. This is supported by previous works that have reported that the penetration of PTF into MOF pores influences the transition energy barrier when driving crystallographic phase transitions with applied hydrostatic pressure[43,50,51]."

"As with the $\mathrm{d}T_0/\mathrm{d}p$ curves from the different trials on *Batch A*, the $\mathrm{d}T_0/\mathrm{d}p$ curves for the different batches are monotonic".

The variation between trials and batches is likely due to small differences in adsorbed nitrogen in the frameworks of our ZIF-4 samples at the start of each trial or measurement of each batch. This feature is likely underpinned by the porosity and rich energy landscape of this porous MOF, which is discussed in the literature. Differences in nitrogen adsorption in ZIF-4 have been demonstrated along with structural data in a few studies [Gandara-Loe *et al*. *J. Mater. Chem. A* **7**, 14552–14558 (2019), Bennett *et al. J. Am. Chem. Soc.* **133**, 14546–14549 (2011), Jain *et al. Inorg. Chem.* **62**, 20, 7703–7715 (2023), Battisti *et al. Microporous Mesoporous Mater*. **143**, 46–53 (2011)]. Additionally, variation in transition pressures when using different pressure transmitting fluids has also been discussed [Vervoorts *et al. Z. Anorg. Allg. Chem.* **645**, 970 – 974 (2019), Henke *et al*. *Chem. Sci*. **9**, 1654-1660 (2018), Widmer *et al*. *J. Am. Chem. Soc.* **141**, 9330-9337 (2019)]. These studies discuss the effect of potential partial penetration of chain ends of the organic pressure transmitting fluids (silicone oil and Fluorinert) into the 'neck' of the ZIF-4 pores under high pressure (no pore 'overloading' unlike with nitrogen), resulting in higher transition pressures than when using mercury, highlighting the dynamic nature of the transition in ZIF-4, and its sensitivity to adsorbed matter.

To clarify this, we have now added these references and the following text in the manuscript when explaining the deviation from ideality of the 0 bar transition temperature after high-pressure measurements due to nitrogen desorption on removing the sample from the high-pressure calorimeter:

"From experimental measurements, nitrogen adsorption-desorption isotherms of desolvated ZIF-4(Zn) have been reported[30,45,46] starting from vacuum pressures (down to 10^{-9} bar) up to ambient pressure (~1 bar) at 77 K, 273 K and 293 K. On pressurizing to ~1 bar at 77 K, some studies[30,45] report an adsorption volume of ~270 $\mathrm{cm^3\ g^{-1}}$ under equilibrium conditions that took ~24 hr for a full measurement[30], whereas another study[46] reports ~15 $\mathrm{cm^3\ g^{-1}}$, which is much lower likely due to kinetics. All three studies show remnant adsorbed nitrogen in the

pores after full depressurisation at 77 K. However, at 273 K and 293 K, the desorption isotherm from the study[46] that reported lower adsorption volumes showed no remnant nitrogen after depressurisation. These reports indicate that prolonged exposure to nitrogen gas can result in some remnant adsorbed nitrogen volume, with a higher adsorption favourability at low temperature. A computational study[47] using Monte Carlo simulations reported nitrogen adsorption isotherms at 298 K from vacuum pressures (0.01 bar) exceeding ambient pressure up to 100 bar, showing a steady increase in volume of adsorbed nitrogen beyond ~1 bar up to ~ 50 bar, before dropping by ~25% on reaching 100 bar. Our sample was exposed to nitrogen in our HP-DSC between ambient pressure and ~1200 bar, over temperatures between 228 K and 343 K. Taking into account the general favourability for desorption at higher temperatures[46], and data from simulations[47] of nitrogen-loaded ZIF-4, some excess adsorbed nitrogen is likely getting desorbed at a certain pressure-threshold below p ~ 200 bar within this temperature range, and during sample transfer at ambient temperature and pressure."

Targeting specific transition temperatures with a known amount of adsorbed nitrogen can be achieved using dedicated gas adsorption equipment (volumetric or gravimetric sorption analysers eg. Quantachrome Autosorb iQ2 unit). This will be the subject of future studies and we thank the Referee for the inspiration, which we now capture in the revised manuscript –

"Precision loading of small amounts of nitrogen adsorbent in to ZIF-4 pores, along with careful tailored synthesis protocols to produce small homogenous particle sizes would be important for applications."

"To achieve this, our findings should motivate focus on controlled synthesis and guest-loading in ZIF-4(Zn) to target specific adsorption volumes and hence finely control barocaloric performance using guest molecules as a caloric vector."

3. In its current form, the manuscript provides little data, mechanistic understanding, or evidence explaining how nitrogen uptake impacts ambient pressure transition temperature performance, key to the author's claim of nitrogen tunable ZIF-4(Zn). Figure 3 offers a qualitative study relating nitrogen exposure to transition temperature and entropy but the changes in entropy are an indirect measure of the nitrogen uptake. How did the authors reach these labels of partially nitrogenated and nitrogenated? To understand how nitrogen uptake impacts transition behaviour, the data presented here likely need more than one point between the desolvated and fully nitrogenated form.

We have now included calorimetric data from two more specimens of ZIF-4 *Batch A* that were exposed to nitrogen for ~12 hours and 22 hours, and show the thermodynamic changes as a function of nitrogen exposure time. The 9-hour and 12-hour exposure sample data are quite similar, as are the 22-hour and 200-hour data. The new Figure 1(f) is reproduced below:

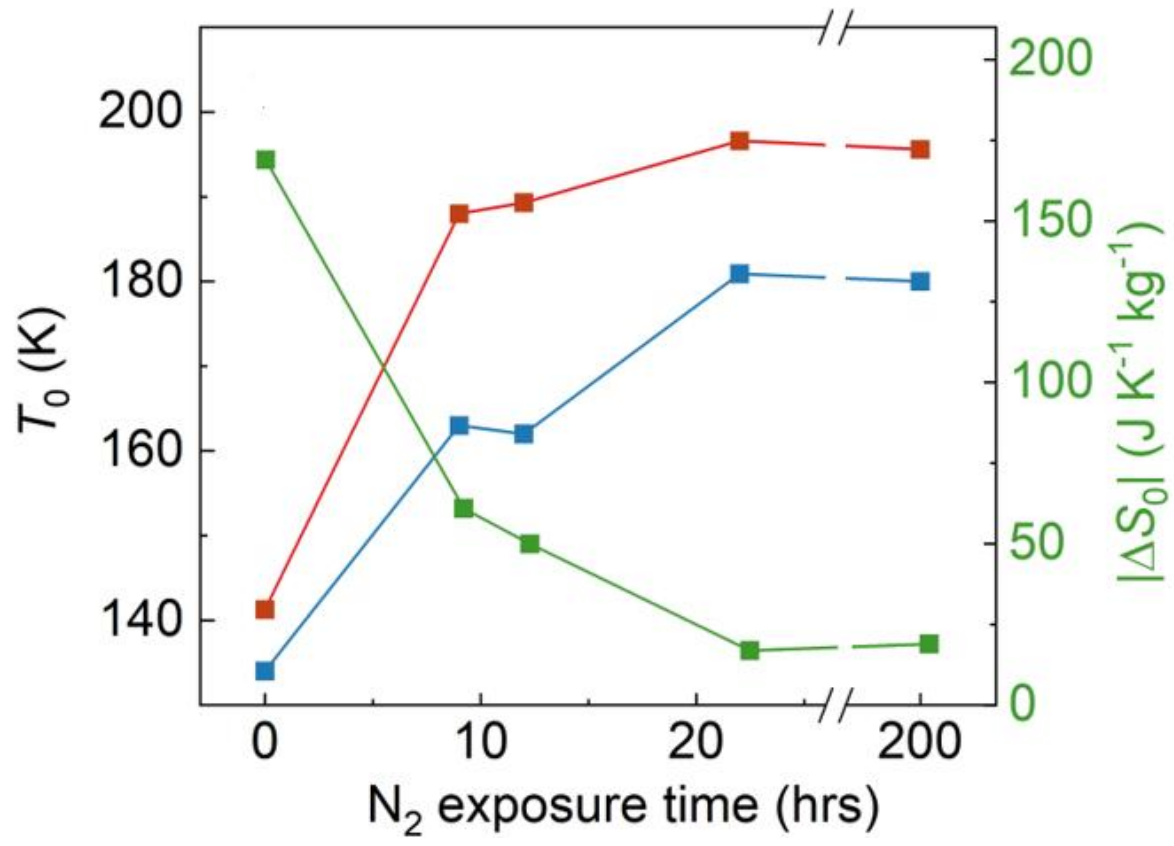

New Figure 1(f). Transition temperature and thermally driven entropy change for the first-order transition in ZIF-4(Zn) as a function of exposure time to N_2.

(We had previously assigned the term *nitrogenated* to samples that were exposed to nitrogen for 22 hours or more, which consistently displayed T_0 ~ 190 K, the highest transition temperature at atmospheric pressure, suggesting a potential saturation in adsorbed nitrogen. We have now added the following text:

"The similarity of T_0 for the 22 hour and 200 hour exposure samples suggest a potential saturation of nitrogen adsorbed within the pores, and we will refer to this structure as nitrogen-loaded ZIF-4(Zn)."

We have also now clarified our experimental procedure for this assessment in the Methods section –

"To determine the effect of nitrogen exposure on ZIF-4(Zn), two separate ~15 mg sample specimens of pristine, desolvated ZIF-4(Zn) from *Batch A* were loaded into the sample cell of the Setaram μDSC7, brought down to T = 233 K and exposed to nitrogen gas at set applied pressure p ~ 1000 bar for 9 hours and 12 hours. After the exposure, the temperature was brought back up to 293 K, the applied pressure released and the samples transferred out of the HP-DSC to small aluminium crucibles to be run in the bespoke calorimeter to evaluate the ambient-pressure calorimetric signal after nitrogen exposure."

4. In several places, the authors offered a normalization of their measured entropy to 'active' mass (in lines 67–68 for the desolvated structure and again in Figure 4 for the post-nitrogen exposed sample). The authors justify this practice given that irreversible phase transitions of this material result in some fraction of 'inactive mass' during phase transition cycling. However, the 'inactive mass' does not appear capable of regeneration, so normalizing the transition entropy based on 'active mass' seems misleading. While this analysis may suggest that the entropy change is higher than measured, this practice makes it challenging for new readers to understand the actual performance of a given material.

The presence of active and inactive material (mass) is common in the field of calorics, and accounting for the presence of inactive mass is good practice, e.g. in magnetocalorics [Moya *et al.*, Nature Materials **12**, 52 (2013)] and in electrocalorics [Nair *et al.*, Nature **575**, 468

(2019)]. The presence of inactive mass does not stop caloric materials from being used in cooling systems. For example, the electrocaloric materials reported by Nair *et al.* [Nature **575**, 468 (2019)] have been successfully used in several electrocaloric prototypes, e.g. Torelló *et al.*, Science **370**, 125 (2020), Wang *et al.*, Science **370**, 129 (2020) and Li *et al.*, Science **382**, 801 (2023).

The purpose of normalising $|\Delta S_0|$ by active mass is strictly thermodynamic, and is necessary to understand the actual barocaloric performance of the ZIF-4 system (e.g. when using thermodynamic relations such as the Clausius-Clapeyron). We are sorry to see that this information was not clearly explained in the manuscript, and for this we apologise. To rectify the matter, we now clarify this normalisation in the revised manuscript for

1. the desolvated structure:

"In order to estimate the pressure-induced shift in transition temperature, $\mathrm{d}T_0/\mathrm{d}p$, of this volume transition using the temperature-pressure Clausius-Clapeyron relation, we normalised $|\Delta S_0|$ by active mass".

2. the nitrogen-loaded structure:

"However, it has also been established from LT-XRD patterns that these $|\Delta S_0|$ values evaluated from integral areas of the calorimetric peaks from (and after) high-pressure measurements (Fig. 2a) are highly reduced due to mass-normalisation that includes inactive sample mass from ZIF-*zni* and large ZIF-4 crystals. Therefore, evaluation of the barocaloric effect in nitrogen-loaded ZIF-4(Zn) alone, associated with the crystallographic phase transition, requires a correction for the active sample mass. We note that coexistence of active and inactive material (mass) is common in e.g. magnetocalorics[48] and electrocalorics[49], and correction for active mass is standard practice."

5. Figure 1 appears to be the only DSC measurement provided for the desolvated ZIF. However, the authors then later provide isothermal entropy curves and adiabatic temperature changes for the desolvated ZIF in Figure 7. From the authors' discussion, this desolvated framework seems like it cannot be exposed to nitrogen without undergoing a transition to the much lower entropy-change material. In this case, how could the authors have constructed isothermal entropy curves that are determined from high-pressure DSC measurements? The underlying data for the red plots of isothermal entropy changes and adiabatic temperature changes in Figure 7 are essential to understand and support one of the central claims stated in the abstract: "For desolvated frameworks of ZIF-4(Zn), we report larger barocaloric effects of |ΔSit| ~ 210 J K−1 kg−1 and |ΔTad| ~ 60 K near T0 ~ 137 K driven using the same pressure change." If these plots are estimated based on other data sets (for example, the high-pressure DSC of the 'nitrogenated' ZIF), this should be clearly stated, and the relevance of such estimates would seem highly uncertain.

The barocaloric effect in desolvated ZIF-4 was estimated from thermodynamic analysis using

1. The 0-bar calorimetric measurement of desolvated ZIF-4
2. LT-XRD analysis of desolvated ZIF-4
3. The high-pressure calorimetric measurements of nitrogenated ZIF-4
4. The Clausius Clapeyron relation

The use of the high-pressure entropy function of the nitrogenated sample is justified given that we are driving the same crystallographic transition (we have included a new figure (shown below) showing comparison between the transition pressures reported in the literature for desolvated ZIF-4 and our transition pressures from our isothermal calorimetry, which clarifies this). The difference is that one structure has empty pores and the other is loaded with nitrogen. We account for this difference from a thermodynamic standpoint by (i) using our 0-bar calorimetry of desolvated ZIF-4 for T_0 at ambient pressure to obtain a $\Delta S(T)$ function at p = 0 bar; (ii) LT-XRD analysis to evaluate ΔV and active mass from quantitative phase analysis; (iii) the high-pressure entropy change function for nitrogen-loaded ZIF-4 given that applied pressure is driving the same particular volumetric transition of concern; and (iv) the Clausius-Clapeyron relation to evaluate the thermodynamic shift of the transition expected from the volume change in a crystal with no adsorbent. This calculation is outlined in the Methods section.

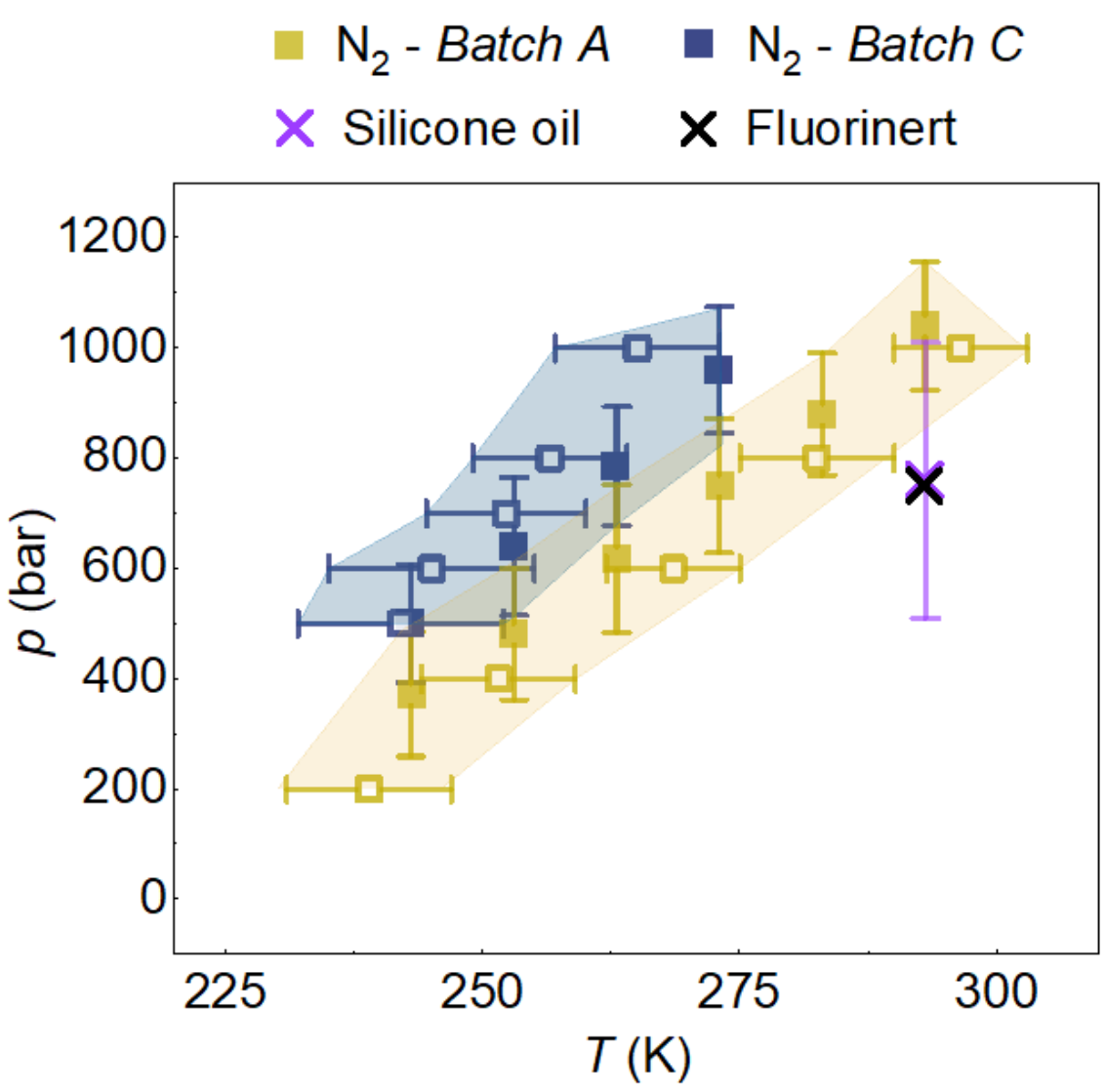

New Fig. 6f. Transition regimes in *T-p* space for nitrogen-loaded ZIF-4(Zn) (squares) from *Batch A* Trial 2 (yellow) and *Batch C* (blue) evaluated from isothermal (solid squares) and isobaric calorimetry (empty squares). Literature values of p_0 reported for driving the transition in desolvated ZIF-4(Zn) (crosses) using Fluorinert (black) and silicone oil (purple) are included for comparison. All bars indicate the widths of the transition. Shaded yellow, red and blue regions in **e** and **f** signify *T-p* regimes for the transition in *Batch A*, *Batch B* and *Batch C*, respectively.

We have also added the following text in the revised manuscript to explain this:

"For comparison, Fig. 6f shows the reported literature values of $p_0 \sim 750$ bar when driving this crystallographic transition in desolvated ZIF-4(Zn) using different PTFs [silicone oil[44] (AP 100) and Fluorinert[43] (FC-70)]. The transition onset using silicone oil as the PTF was reported

at ~500 bar, with completion occurring just above ~1000 bar. Our measurements of $p_0(T)$ for nitrogen-loaded ZIF-4(Zn) from isothermal measurements are in agreement with these reported values for desolvated ZIF-4(Zn), which indicates that it is the same crystallographic transition being driven."

Nevertheless, since our functions of caloric effects for desolvated ZIF-4 are based on thermodynamic evaluations, we have now changed our wording to "estimate" in the abstract and in the main text.

6. Beyond the technical issues described above, the manuscript formatting makes the text very challenging to follow. There are numerous times throughout the manuscript where the authors introduce a concept that they say they will explain later in the manuscript. While this occasionally is reasonable, the text here uses this tool much too often. The strategy of referencing future sections means that readers must jump around the manuscript frequently to understand non-standard concepts. The readability of the manuscript would need to be improved to be accessible to the broad readership of Nature Communications.

We have now brought the "Deviation from ideality" section into the section on "quasi-direct evaluation of the barocaloric effect", immediately after introducing the concept of desorption affecting the transition temperature.

We have also removed the reference to the later section on "Particle size" at the start, and have instead referenced the literature paper that suggested impact of particle size on the transition.

We have also changed the colour schemes in all figures according to the Editorial suggestions.

Minor Comments:

1. In multiple cases, the authors use the descriptor 'nitrogenated' to describe the framework with nitrogen gas present. This verbiage, nitrogenated, is often used to describe a covalent chemical transformation, whereas the effect here is adsorption and desorption of nitrogen gas. This wording should be amended in all cases to something related to nitrogen gas uptake.

We have changed all occurrences of 'nitrogenated' to 'nitrogen-loaded'.

2. Despite the comments for SEM particle sizing errors, could the author's include error bars within Figure 9?

We have added error bars to the data shown in Figure 9. This figure has now been moved to the Supplementary Information (Supplementary Figure 13).

3. Could the authors expand on their comments in lines 306-308 'Calorimetric data for Batches B and C under applied pressure showed multiple peaks shifting with pressure, indicating the dynamic impact of nitrogenation on the transition.' How does shifting of multiple peaks show the dynamic impact of the application of pressure?

What we meant was the dynamic impact of sorption/desorption causing the peaks to split and broaden as seen in isobaric calorimetry for *Batch C* (from isothermal and isobaric calorimetry, we establish that the calorimetric peaks we observe are for the same transition in concern, as shown in New Fig. 6f). We have now clarified this in the manuscript and removed the reference to *Batch B*, as there is a transition unaccounted for near the main transition temperature on both heating and cooling, and we didn't have enough sample left to test using isothermal calorimetry. We have now brought the calorimetric cycles of *Batches B* and *C* from the Supplementary Information to the main text (New Fig. 6c,d) and have added the following text :

"Fig. 6f shows transition regimes in *T-p* space for nitrogen-loaded ZIF-4(Zn) from *Batch A* and *Batch C* evaluated from isothermal and isobaric calorimetry. From corelation of transition temperatures T_0 and pressures p_0 in *T-p* space (Fig. 6f), and latent heats evaluated using isobaric and isothermal calorimetry, it can be established that the transitions observed from our direct and quasi-direct analysis of *Batch C* are identical. Therefore, the splitting transition peaks on cooling in each isobar in Fig. 6d likely indicate different degrees of nitrogen adsorption within the crystals."

4. Why does the DSC peak shape change at higher temperatures in Figure 8?

This behaviour is commonly seen in magnetocalorics, electrocalorics and barocalorics [Stern-Taulats *et al.*, *J. Appl. Phys.* **115**, 173907 (2014), Farenkov *et al. APL Mater.* **12**, 111113 (2024), Yuce *et al. Appl. Phys. Lett.* **101**, 071906 (2012)], when the applied field is large and the caloric material approaches the critical point.

5. Could the authors provide a timescale for the pressurization and depressurization in Figure 8?

The pressure ramps were $|dp|/dt$ = 13 bar min^{-1}, such that pressurisation took ~85 mins, and depressurisation took ~85 mins.

6. What are the thermodynamic parameters of Batch D and E? Particle sizes for E are used for comparison, but only the DSC traces are shown in Figures S14 and S15, with no analysis in the main text.

This is because Batches D and E showed flat baselines with no transition at all – we have now included this raw data in the Source Data file. We wanted to report samples that showed no transition, because we have found contradictory reports in the literature that claim that while some studies show the transition at 0 bar, their studies did not. We also found that some of our samples (*Batch A, B* and *C*) showed the transition, while *Batch D* and *E* did not. The most

likely explanation based on details from the studies in the literature and our own, is particle size. The recent study by Li *et al.* [Journal of Materials Science & Technology **264**, 82 (2026)] is in line with our statement on particle size – their samples are ~5.8 μm.

7. Line 42 – "low applied pressures below 1 kbar". The word low should be removed. Vapor-compression systems run in the 'low' pressure range 2-20 bar which is well below the ceiling of 1 kbar.

In the barocaloric community, applied pressures below 1 kbar are considered low. This is because 1 kbar can be routinely and inexpensively achieved using off-the-shelf hydraulic equipment, which is the equipment used in real-world applications (www.barocal.com). By contrast, 5 kbar are needed to drive barocaloric effects reversibly over a 25 K temperature range in prototypical neopentyl glycol [Lloveras *et al.*, Nature Communications **10**, 1083 (2019)]. In other areas of solid-state physics, high pressures are 1 GPa and above.

Nevertheless, we have changed "low applied pressure" to "relatively low applied pressure" in the manuscript.

We note that the comparison with vapour-compression is not relevant, as our solid refrigerants display small changes in volume when driven using pressure, such that there is no risk of explosion.

Referee #2:

We thank the referee for the positive and excellent feedback, which we have used to improve the manuscript. Purely for brevity, our response does not thank the referee for each of the individual technical comments, but the referee should rest assured that we really appreciate the input.

We thank the referee for the detailed feedback, which has inspired us to make major improvements. Purely for brevity, our response does not thank the referee for each of the individual technical comments, but the referee should rest assured that we really appreciate the input.

All changes (except figure changes) are shown using yellow highlights. These changes include changes due to feedback from all referees, changes where we found ways to improve the presentation, and changes that we made to comply with your Editorial advice to e.g. include a Data Availability section after the Methods section but before the References.

Figure changes are as follows:

- In Fig. 1(e), using the feedback of Referee 1, we have added calorimetry curves at 0 bar for samples after nitrogen exposure of 12 hours and 22 hours.
- In Fig. 1(f), using the feedback of Referee 1, we now show the changes in the thermodynamic parameters of the first-order phase transition as a function of nitrogen exposure time.
- In Fig. 4, using the feedback of Referee 1, we have removed the linear guide to the eye.
- In Fig. 6(f), using the feedback of Referee 1, we have added a graph showing comparison between transition pressures required to drive our ZIF-4 samples with those reported in the literature.
- Using the feedback of Referee 2, we have merged Figs 1-3, and Figs 8 and 10, in the original manuscript, and moved Fig. 9 to Supplementary Information. Also, we now show Figs S15(a) and (b) in the main manuscript as Fig. 6(c,d).
- All figure colour schemes have been improved for better readability.

The revised abstract now has 200 words, and the revised main text now has 4823 words, which is near the specified limit of 5000 words. The source data for calorimetry and scanning electron microscopy are included in a zipped folder labelled Source Data, along with x-ray diffraction data in the University of Cambridge repository Apollo.

Dr Dilshad and colleagues report giant but yet tunable barocaloric effects in the metal-organic framework (MOF) ZIF-4(Zn). Porosity is a key feature of MOF families. The authors noticed the effect of guest molecules in ZIF-4 and uncovered their function in response to pressure and the barocaloric effect. In detail, ZIF-4 presents a giant entropy change of 38 J K^{-1} kg^{-1} when N_2 is occupied in the pore. When the N_2 is removed from the pore, a colossal entropy change of 210 J K^{-1} kg^{-1} is obtained instead. This study highlights the potential of MOFs as promising barocaloric refrigerants and also a new cooling technology by tuning the amount of guest molecules.
In my opinion, this paper shows great novelty in presenting a colossal barocaloric effect in ZIF-4, while opening a way to manipulate the barocaloric effect in MOFs through their porous structural property. MOFs are a large family of materials with designable porosity. I believe that this work could potentially establish a playground for MOFs as environmentally benign solid-state refrigerants that can be tuned using guest molecules. Therefore, I would highly recommend this work for publishing in Nature Communications.

We thank the referee for highly recommending publication.

Also, there are some minor corrections for your consideration:

1. It would be nice to present a simple crystal structure of ZIF-4(Zn) in Fig. 1 or in the figures in the Supplementary Materials for readers to better understand ZIF-4(Zn) and its porosity.

We have now included a structure in the Supplementary Information (Supplementary Fig. 2).

2. It is interesting to know the structural stability or durability of ZIF-4 with and without N_2 gas under the pressure used in this work.

We performed quasi-direct and direct measurements in 3 separate samples, with >40 combined cycles in a single specimen.

Moreover, a recent study by Li *et al.* [Journal of Materials Science & Technology **264**, 82 (2026)] reports large barocaloric effects in unloaded ZIF-4(Zn) and shows stability with over 100 heating and cooling cycles, before stopping testing.

3. In line 49, the authors prepare activated ZIF by removing solvent from pores. How about the N_2 and O_2 from air—are they also removed?

No they were not - all samples were evacuated under dynamic vacuum only after synthesis. We did not specifically evacuate them prior to loading in the calorimeter. Therefore a small amount of atmospheric nitrogen and moisture may have been adsorbed during storage. This did not seem to have much effect for a few days after synthesis and evacuation, as shown by our

ambient pressure calorimetry peaks. However, we believe this contributed to the diminished caloric effect after 17 months. We have now added a statement in the revised manuscript to state this.

4. In line 261, the authors integrated the isothermal calorimetric peaks to get the isothermal heat. If I understand it correctly, one must have a reference value under ambient pressure and then normalize all the peak values from ambient pressure to high pressure. But this is not the case for a custom high-pressure DSC set-up. So it would be good to point out the set-up used here.

The method that the referee describes is normally used when the thermal sensor (thermocouple) is in contact with the pressure-transmitting media and therefore exposed to high pressures, such that no other type of calibration is possible. This was the method in e.g. Mañosa *et al.*, Nature Materials **9**, 478 (2010), and Lloveras *et al.*, Nature Communications **10**, 1083 (2019).

In our DSC, the thermal sensors (Peltier elements) are not in contact with the pressure-transmitting media and therefore remain at atmospheric pressures, such that standard calibration via dissipation of Joule heating is possible.

5. In the section "Evaluation of barocaloric effects" in Methods, the specific heats of LT and HT are essential for constructing the entropy change curves. Is there any difference in specific heat between nitrogenated and desolvated ZIF?

There is a small difference, but this difference is within the error of the measurements.

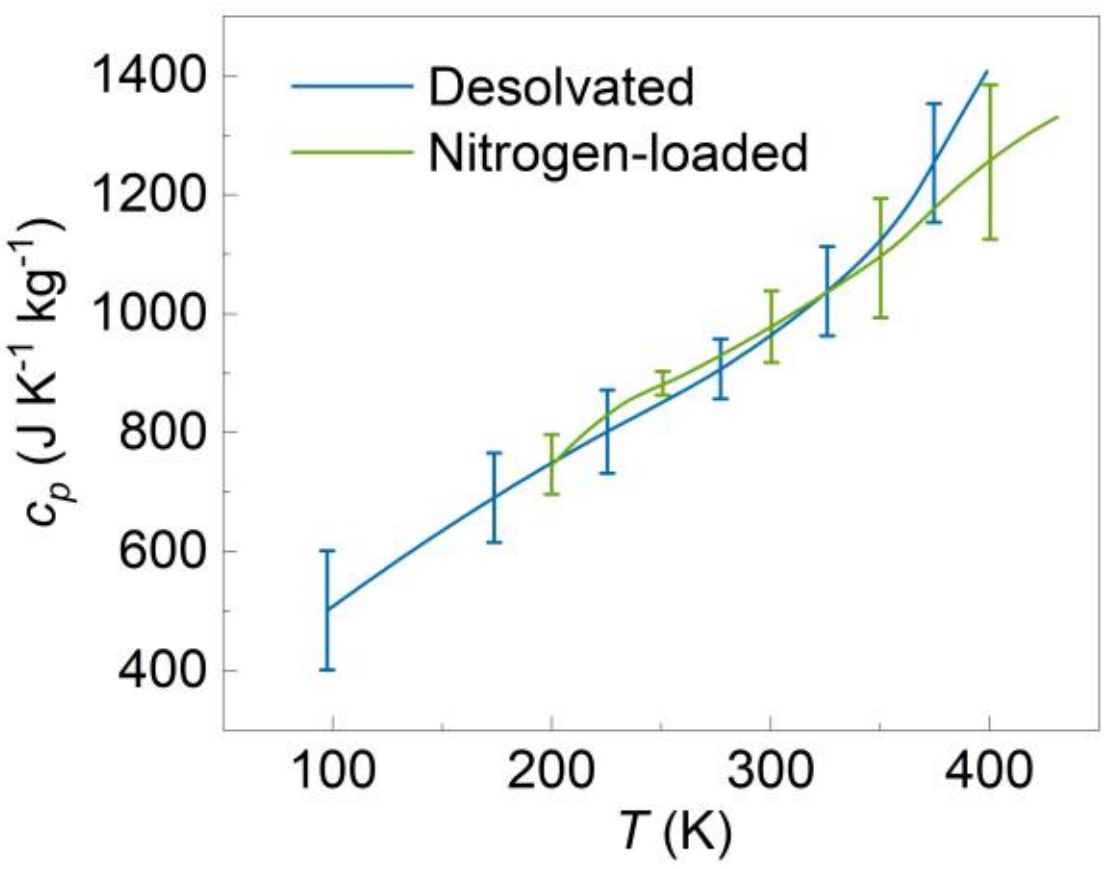

Suggestion for layout:

1. Please check the reference style in the main text. The authors may need to change "ref [xx]" or "[refs. xx]" directly to "[]".

We have now changed the reference style accordingly.

2. There are too many figures in the main text; please make sure this is in line with the construction requirements of Nature Communications. The authors may consider merging some figures.

We have now merged Figures 1-3, and Figures 8 and 10. We have brought some relevant figures from the Supplementary Information to the main manuscript in the new Figure 6. There are now 6 figures in total in line with Journal requirements.

3. It is very interesting to see the influence of particle size on the barocaloric response. I would suggest merging Figures 8 and 10 to make the storyline clean, and putting Figure 9 into the Supplementary Materials.

We have now merged Figures 8 and 10 and moved Figure 9 to the Supplementary Information.
